# Enrichment on steps, not genes, improves inference of differentially expressed pathways

**DOI:** 10.1371/journal.pcbi.1011968

**Published:** 2024-03-25

**Authors:** Nicholas Markarian, Kimberly M. Van Auken, Dustin Ebert, Paul W. Sternberg

**Affiliations:** 1 Division of Biology and Biological Engineering, California Institute of Technology, Pasadena, California, United States of America; 2 Keck School of Medicine, University of Southern California, Los Angeles, California, United States of America; 3 Division of Bioinformatics, Department of Population and Public Health Sciences, Keck School of Medicine, University of Southern California, Los Angeles, California, United States of America; Universite de Lausanne Faculte de biologie et medecine, SWITZERLAND

## Abstract

Enrichment analysis is frequently used in combination with differential expression data to investigate potential commonalities amongst lists of genes and generate hypotheses for further experiments. However, current enrichment analysis approaches on pathways ignore the functional relationships between genes in a pathway, particularly OR logic that occurs when a set of proteins can each individually perform the same step in a pathway. As a result, these approaches miss pathways with large or multiple sets because of an inflation of pathway size (when measured as the total gene count) relative to the number of steps. We address this problem by enriching on step-enabling entities in pathways. We treat sets of protein-coding genes as single entities, and we also weight sets to account for the number of genes in them using the multivariate Fisher’s noncentral hypergeometric distribution. We then show three examples of pathways that are recovered with this method and find that the results have significant proportions of pathways not found in gene list enrichment analysis.

## Author summary

Genome-scale experiments typically identify sets of genes which are primarily analyzed by enrichment analysis to identify relevant pathways that may be perturbed. Curated pathway models have rich structure that we believe can be exploited to get better results. Some pathway steps are enabled by sets of interchangeable genes which inflate the gene count of their respective pathways relative to the number of steps. We improve sensitivity towards these pathways in enrichment analysis by performing enrichment on steps. We then use this approach to identify pathways that would otherwise be missed in medically relevant datasets to gain new insights.

## Introduction

High-throughput experiments regularly output large lists of genes that vary in expression across conditions and cell types or are perturbed in disease states (e.g. 1,2). While these experiments can establish transcriptional signatures for cells or diseases, the interpretation of these lists of genes in the context of physiology or phenotype can prove difficult, even for domain experts, as the collective body of knowledge in the literature grows at an ever increasing rate [3]. Enrichment tools aim to aid that analysis by comparing annotations, such as disease, process, and pathway associations, associated with the outputted list of differentially expressed genes to those of sets of genes in databases that previous studies have linked to specific diseases, biological processes, and pathways [4–7].

While the enrichment analysis field has made many advances, it has treated pathway enrichment the same as enrichment on categorical terms without consideration for pathways’ inherent structure. In its simplest form, enrichment analysis searches for overrepresented annotations within lists of genes. It performs pairwise evaluations of the overlap between the list from a particular experiment and reference lists in knowledgebases to determine if any of those overlaps are greater than would be expected by chance. Early work searched for overrepresentation in categories such as diseases or particular Gene Ontology (GO) terms for cellular compartments, molecular functions, or biological processes [6,8,9]. As pathway databases such as Reactome and KEGG were developed and expanded [10,11], pathways were incorporated into enrichment analyses by applying the same algorithms and treating pathway membership as an annotation to form a list, although this eliminated causal relationships and pathway structure. Later work introduced more sophisticated statistical procedures to address open problems in enrichment such as utilizing fold changes in expression [12], leveraging relationships between annotation terms [7], or incorporating protein-protein interaction networks [13], but these methods still treated pathways the same as other reference gene sets, considering pathway membership as an annotation. Unlike the previously mentioned classifications for genes, such as the category of genes with products active in a specific organelle, biological pathways have structure (i.e., have causal, directional relationships between participating genes), and thus are not simply categories or lists, but this issue has not been addressed.

We utilized an aspect of pathway structure, sets, in our enrichment on pathways modeled in Gene Ontology Causal Activity Models (GO-CAMs) [14], and this enabled us to recover pertinent biological pathways that could otherwise be missed. GO-CAMs are a type of pathway model centered around GO molecular functions and use other ontology terms to provide relevant biological context. GO-CAMs are typically curated manually by members of the GO consortium [15], but an additional source of human GO-CAMs is computationally generated from conversion of pathways in Reactome [16], a popular pathway database also used to define gene lists for pathways in widely used enrichment analysis tools such as PANTHER and DAVID [4,7,10]. In examining causal flow in GO-CAMs, we realized that another relationship between genes annotated to pathways has been neglected in the conversion to lists but is becoming recognized in other analyses [17]: OR logic via interchangeability of gene products at certain steps in pathways. This interchangeability is represented explicitly in Reactome (and by extension, GO CAMs) as “sets,” defined as groups of proteins or protein complexes that are individually sufficient to perform the same step in a given pathway, and implicitly in KEGG, where they can be inferred by annotation of multiple Enzyme Commission numbers to a reaction, making our work broadly applicable. For example, the Reactome set “Glucokinase and Hexokinases” is comprised of glucokinase and hexokinases 1, 2, and 3. Any one of these proteins is sufficient to phosphorylate glucose, the first step in the glycolysis pathway. Furthermore, glucokinase is only expressed in the liver and pancreas and is not available to be up or downregulated by other cell types. Thus, sets can either be a consequence of annotation decisions, such as using one pathway diagram that may differ from cell type to cell type, or they can be a direct representation of biology, where multiple gene products may substitute for one another, albeit with potentially distinct reaction kinetics.

We can contrast sets with complexes in terms of logic. For example, microtubules are formed from tubulin *α*∙*β* dimers. Microtubule formation requires both tubulin *α* AND tubulin *β*. In contrast, phosphorylating glucose requires either glucokinase OR hexokinase 1 OR hexokinase 2 OR hexokinase 3. (In fact, there are actually 8 genes that encode α tubulins and 9 genes that encode β tubulins, many of which have cell or tissue specific expression, so sets can be found in the context of complexes as well [18]). Sets enable curators to avoid creating multiple, otherwise redundant instances of pathways when different gene products may perform the same step in different cells or within the same cell; a single instance of a pathway model is created, and the set indicates the variability at that step. Ideally, enrichment analysis would acknowledge this variability and have some degree of robustness to the decision to annotate additional genes that can enable a pathway.

However, widely used enrichment tools such as those at PANTHER and Reactome do not account for these sets, nor does any other tool of which we are aware. This can be problematic, because sets inflate the count of all genes annotated to a pathway when they are expanded to create a gene list for enrichment, but they do not increase the number of steps. For example, the BMP signaling pathway has 7 receptors, each individually sufficient to facilitate signaling, and they are expressed in many tissues at varying levels [19]. There are many other steps in this pathway, but for argument’s sake, suppose there were only two other steps, one enabled by a complex of two gene products and the other enabled by one gene product, for a total of 10 genes annotated to the pathway. If a cell upregulated expression of one member of the set of receptors and the single gene product for the last step, this scenario will be treated as 2 of 10 genes in the pathway, even though 2 of 3 steps are affected. Furthermore, complexes are treated the same as sets even though the logical relationship between their members differs. Increased expression of just one member of the proteosome complex likely does not mean increased proteasome activity, but increased expression of a member of a set of enzyme activators, receptors, or enzymes may be impactful. Due to the inflation of *n*, the gene count of the pathway, the pathway may not be captured by the enrichment analysis. Researchers using enrichment tools usually seek to uncover which pathways are more active in different conditions, a question that is more directly dependent on the proportion of steps in a pathway that are up and down regulated than on the proportion of genes annotated to a pathway, given that some of those genes can act in each other’s stead.

This study implements enrichment on Gene Ontology Causal Activity Models (GO-CAMs) [14] and explores the impact of “sets,” a feature in pathway models neglected in current enrichment tools, seeking to integrate it into analysis. We discover that some very large gene sets greatly inflate the gene count of the pathways in which they are members if sets are treated the same as complexes, impeding the pathways from being captured by enrichment analysis tools. We propose accounting for this by performing enrichment analysis on the pathway steps rather than directly on the genes themselves. Using a one-tailed hypergeometric test while treating sets as single entities, we showcase three examples of enriched pathways and then evaluate results on datasets from six studies [20–25]. We show that while the enrichment results largely overlap with those yielded by enriching directly on the list of genes, a significant proportion of results are unique. Lastly, we consider how the assumptions of the null hypothesis change when treating sets as single entities and introduce enrichment analysis on pathway steps via the multivariate Fisher’s noncentral hypergeometric distribution to weight sets according to the number of genes, in line with the traditional assumption that each gene is chosen with uniform probability.

## Results

### Formulating a step-focused null hypothesis for enrichment by including sets

**Fig 1:**
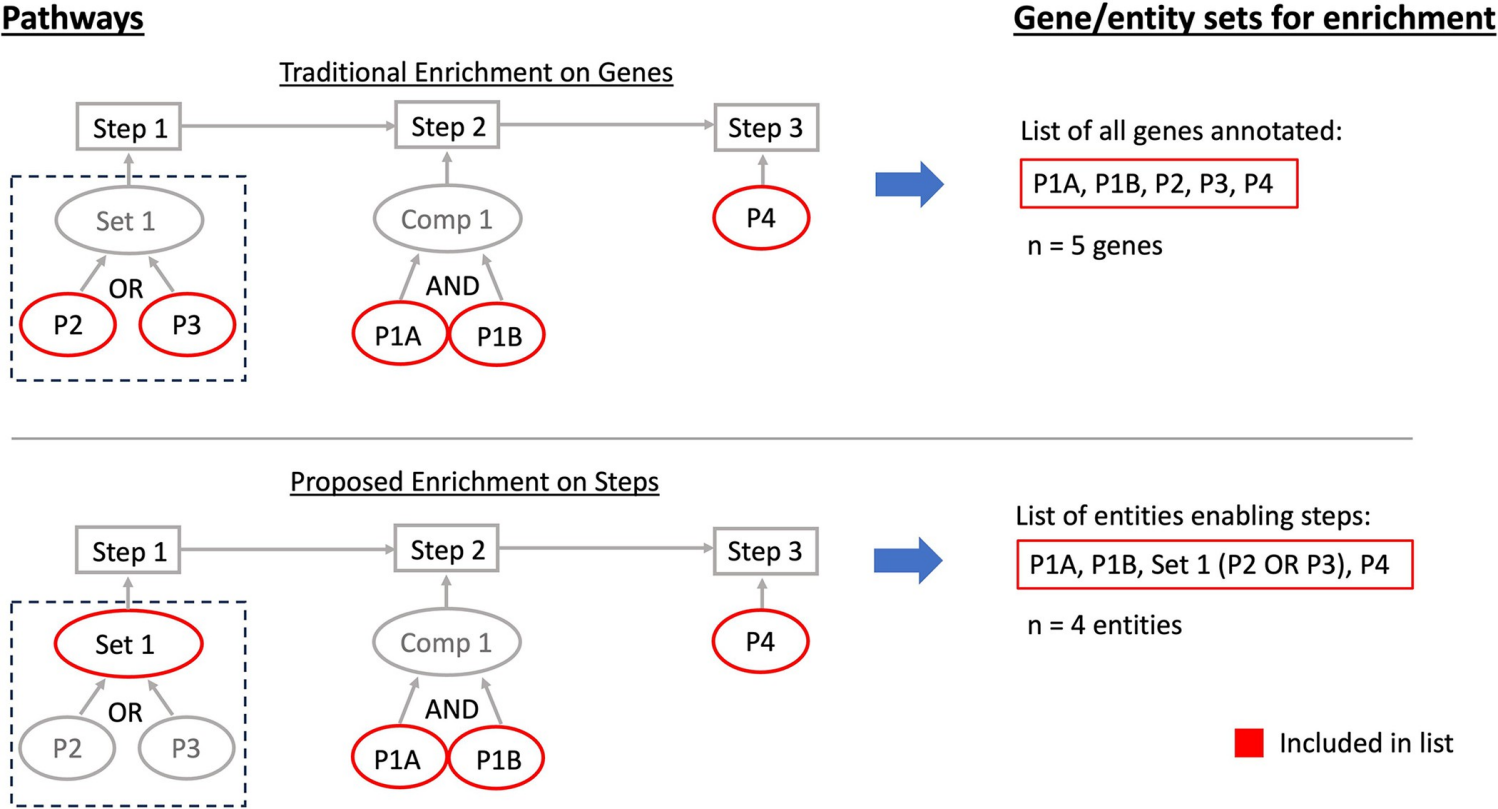
Enriching directly on genes inflates the number of pathway elements relative to the number of steps. The pathway model (in blue) consists of 3 steps, enabled by a complex, a set, and a protein respectively. Traditional enrichment extracts the list of all genes associated with a pathway, treating complexes and sets equivalently, even though the logical interpretation of a complex is a joining of its members through an AND relation while members of a set are linked by OR. Enrichment on steps accounts for sets while creating the lists, where *Set 1* acts as a placeholder for *“Protein 1 OR Protein 2”*, and *Complex 1* is treated as *“Protein 3A AND Protein 3B”*. The pathway is enabled by “*Set 1 AND Complex 1 AND Protein 4*,*”* which is equivalent to “*(P1 OR P2) AND (P3A AND P3B) AND P4*.*”* Hence, the list we enrich on is “*Set 1*, *P3A*, *P3B*, *P4*,” where *Set 1* is *(P2 OR P3)*. Importantly, the size of the list used in enrichment is equal to the minimum number of genes required to enable the pathway in our step-centric enrichment but not in traditional enrichment on gene lists, which uses the total gene count.

Traditionally, enrichment analysis with the hypergeometric test asks the question “What is the probability that *k* or more out of *n* genes associated with the pathway are in a list of length *N*, where those *N* genes are sampled from a background of size *M*?” We want to propose a question focused on the steps that form a pathway instead. Defining entities as: 1) the proteins, 2) protein subunits of complexes, 3) and sets of proteins that perform steps in a pathway, we ask “Given the steps in the pathway and the entities that enable them, what is the probability that the *k* or more out of *n* entities required to enable those steps are in a list of length *N*, where those *N* entities are sampled from a background of size *M*?” Both null hypotheses assume that each gene or entity is sampled independently and with equal probability. We don’t formally state the question as “What is the probability that *k* or more out of *n* steps in a pathway are sampled,” because complexes are split into their protein subunits, but that is the underlying idea. In a pathway with no complexes, the questions are equivalent, and we want to represent a scenario where cells regulate pathways by selecting steps to regulate without replacement. We illustrate the comparison in the gene lists used by the two methods in Fig 1 and detail the procedures for enrichment in the next section.

### Pathway enrichment procedure

**Fig 2:**
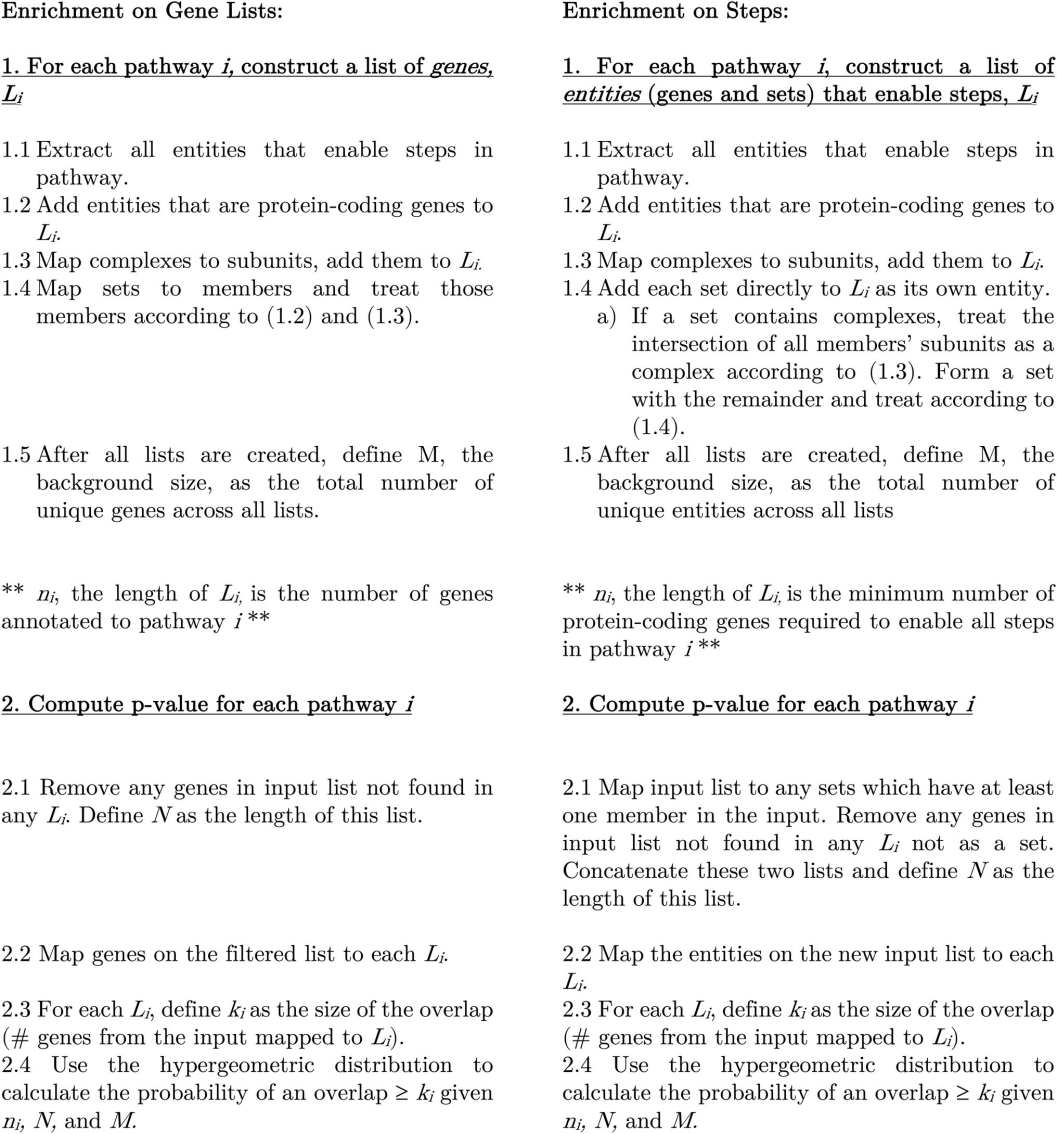
Step by step comparison of enrichment algorithms. Multiple testing correction is not shown here but is done with the Benjamini-Hochberg procedure.

Enriching on steps, as shown above, requires mapping the input list of genes from an experiment to the list of step-enabling entities that those genes belong to. This list consists of 1) any sets that have at least one member in the input and 2) any genes in the input that are the sole genes to enable a step in a pathway (not part of a set). Enrichment can be performed using the one-tailed hypergeometric test with this modified input list and the step-enabling list of entities for pathway *i*, *L*_*i*_. This is also known as a one-tailed Fisher’s exact test [26]. The key result is that *n*_*i*_, the length of list *L*_*i*_, is the minimum number of genes required to enable all steps of the pathway. Traditionally, *n*_*i*_ is the total number of genes associated with a pathway, which could greatly exceed the number of steps in the pathway due to large sets. A comparison of the algorithms is shown in Fig 2.

Complexes pose a design challenge with enriching on steps, because it is unclear what it would mean to alter expression of one of the members of a protein complex but not the others. This depends on whether a particular complex is assembled upon translation or later through protein interactions, as well as knowledge of which proteins are the limiting factor due to stoichiometry and/or assembly kinetics. In addition, some annotated complexes in pathway databases are transiently formed during signal transduction, such as IL7R-JAK-STAT, a complex in Reactome [10]. We decided to treat complexes the same way as they have been previously: each complex is mapped to its protein members, and those proteins are considered part of the list for the pathway, just as each member of a complex is traditionally added to the list of genes for the pathway (e.g. 4). If a protein complex is necessary to perform a step in a pathway, we consider their protein members to be necessary as well, acknowledging the limitation that this allows for partial contribution to enrichment when in some cases, it should biologically be all-or-nothing.

Sets of complexes usually have one or more common subunits and differ only in one subunit, so we create a new complex out of the common subunits (the subunit intersection across all the complexes), and then a new set out of the remainder (Fig 2). That new complex is then mapped to its subunit members, and each is treated as an entity. Except in the cases where the set of complexes is a heterogenous group or has multiple specific subunits, this faithfully represents sets of complexes in a manner consistent with our representation of complexes and of sets of proteins. For example, Prolyl 4-hydroxylase is a complex with 2 P4HB1 beta subunits and 2 identical alpha subunits from P4HA1, P4HA2, or P4HA3. This is represented as a set of complexes (2 P4HA1: 2 P4HB1 OR 2 P4HA2: 2 P4HB1 OR 2 P4HA3: 2 P4HB1), but we represent it with P4HB1 AND (P4HA1 OR P4HA2 OR P4HA3). We recursively apply the above logic to reduce these to proteins and sets.

### Comparing parameter changes in gene list and step-centric enrichment

These changes affect the hypergeometric test primarily by reducing *n*, the size of the pathway against which the overlap, *k*, is compared to. *M*, the size of the background list is also reduced, because ~2300 genes only appear in pathways as part of sets and thus are not unique entities. All else equal, the reduction of *n* lowers the p-value, while the reduction of *M* increases it. We constructed lists of genes for each pathway via the standard gene list method and compared these to the entity lists produced by our method for each pathway. While the change in *n* is pathway-specific, the median reduction per pathway from the gene-list method to ours is 6%, with a 75^th^ percentile reduction of 40%, indicating that most models are unaffected by the change, but a minority are significantly impacted (Table 1). The change in *M* is a reduction from 5386 genes annotated across all pathways to 3983 entities (genes and sets of genes) annotated across all pathways. (Of the 5386 genes, only 3086 are the sole entities that enable at least one step, meaning they appear outside of sets, while 1400 are only annotated as part of sets. There are a total of 915 sets). *N* can change as well, but the magnitude and direction of change are dependent on the input list. Lastly, *k* can be reduced because we only allow for each set to count once towards the overlap, even if more than one gene in the set is on the input list. This reduction is a consequence of enriching on entities and treating each set as one entity. We discuss this issue further in the section on limitations.

**Table 1:** Absolute and relative reductions in pathway sizes (n) from gene enrichment to step enrichment.

PATHWAY SIZE CHANGE (gene list length vs entity list length)	Absolute Reduction (n_gene list_ - n _entity list_)	Relative Reduction (n_gene list_ - n _entity list_)/ n_gene list_
**50**^**th**^ **percentile**	1	3%
**75**^**th**^ **percentile**	4	40%
**95**^**th**^ **percentile**	19	76%
**Max**	255	98%

### Case studies of pathways missed by gene list enrichment analysis

We sought specific use cases of published RNAseq or quantitative proteomics datasets to compare the gene-list method and our step-centric method using two criteria: 1) single cell sequencing or bulk sequencing on only one cell type and 2) that the datasets were generated from different organ systems and different pathologies. We wanted to compare one cell type or state versus another, as our study is motivated by accounting for the sufficiency of members of sets for enabling steps in pathways, and pooling data from multiple cell types or conditions could undermine this and lead to spurious results. For example, a list of mutated genes implicated in gastric cancers do not characterize a single cell state, as these mutations do not all coexist in one cell but are aggregated from many patients’ tumors. We also wanted diversity in our datasets as the GO-CAM pathway database does not yet represent all processes in the cell, and annotations (and therefore enrichment) may be relatively scant for some organ systems or processes. We evaluated enrichment on six datasets, one proteomic, representing cell line response to p97 inhibitors [21], and five transcriptomic: changes in platelets during SARS-CoV-2 infection [20], dilated and arrhythmogenic cardiomyopathy [22], nonalcoholic steatohepatitis [24], astrocytes in various regions of aging mouse brains [23], and activation of macrophages in vitro [25]. These datasets were from the first six papers we found that met our criteria and had readily available differential expression data, as we wanted to provide an unbiased assessment in our intended use case. First, we present case studies from three of these datasets (platelets in SARS-CoV-2 infection, p97 inhibitors, and dilated and arrhythmogenic cardiomyopathy) and then we aggregate results from all six to characterize the divergence between enrichment methods.

#### Platelets in SARS-CoV-2 infection may produce collagen, and VLCFA synthesis is reduced

**Fig 3:**
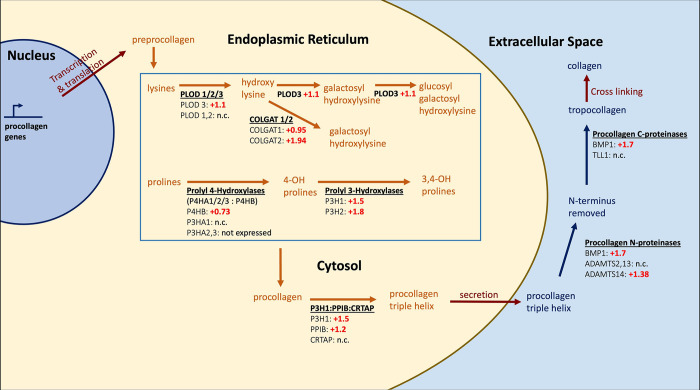
Nearly all steps in the collagen synthetic pathway have at least one gene that is upregulated two-fold in platelets during SARS-CoV-2 infection [20]. Diagram derived from GO-CAM *Collagen Biosynthesis and Modifying Enzymes* (http://model.geneontology.org/R-HSA-1650814) and edited for clarity. Log fold changes shown [20]. Log_2_ fold change of ADAMTS14 of +1.38 is not statistically significant. N.c. stands for “no change.” Sets are denoted with text titles (such as “Procollagen N-proteinases”) or forward slashes (such as “P4HA1/2/3”). Complexes are denoted with colons.

COVID-19 patients suffer from increased rates of thrombotic events, and Manne et al., 2020 sought to understand whether platelets in these patients are more prone to aggregation and, if so, how [20]. They collected platelets from the blood of patients hospitalized with COVID-19, performed bulk RNAseq, and compared them to platelet transcriptomes of healthy donors. While platelets themselves do not have nuclei and cannot synthesize mRNA, they retain mRNA from their megakaryocyte precursors and can synthesize proteins. One striking result we uncovered is the collagen biosynthetic pathway, which has 10 entities of 12 upregulated in SARS-CoV-2 infection (Fig 3), but there are 65 members in the gene list for this pathway (unadjusted p-value of 0.087 for 8/65 genes in our implementation; not reported as a result by PANTHER for 9/67 genes; unadjusted p-value of 0.22 for 9/76 entities in the Reactome Analysis tool due to slight annotation differences [4,10]). GSEA analysis utilizing the log_2_ fold changes yielded an FDR adjusted p-value of 0.52 [12,27]. In this case GO CAM model size is clearly inflated by the set of ‘4-Hydroxyproline collagen propeptides’, which represents 44 procollagen genes. See Discussion for further discussion of the potential significance of this and support in literature, particularly from Kyselova et al. 28, who provided the first biochemical evidence that platelets can be a source of collagen in another disease, Polycystic Ovarian Syndrome. While collagen I levels in COVID-19 patients would need to be assayed to confirm this, and if true, its significance or lack thereof to increased platelet activity in COVID-19 would need to be studied, collagen I is a known activator of platelets, and accounting for sets allowed us to generate a hypothesis that would have been missed by the standard gene list method.

In addition, we found that the pathway “Synthesis of Very Long Chain Fatty Acyl Co-A” (http://model.geneontology.org/R-HSA-75876) is downregulated (6/12 step enabling entities) while this is missed by gene-list enrichment because of pathway size inflation (unadjusted p-value of 0.067 for 5/24 genes in our implementation; not reported as significant for 5/24 genes in PANTHER; with 5/51 entities in the Reactome Analysis tool due to annotation differences) [4,10]. GSEA analysis utilizing the log_2_ fold changes yielded an FDR adjusted p-value of 0.93 [12,27]. This putative downregulation is confirmed in another study of lipids in platelets during SARS-CoV-2 infection, where a specific, class-wide decrease of very long chain fatty acids was observed (see Fig 3B of [29]).

#### P97 inhibitors may alter PI3K signaling

Wang et al. [21] performed quantitative proteomics on cultured cell lines exposed to p97 inhibitors, proteome inhibitors, and DMSO. We analyzed their list of proteins with altered abundance after shRNA knockdown of p97 and found that the gene list method missed *PI5P*, *PP2A and IER3 Regulate PI3K/AKT Signaling* (http://model.geneontology.org/R-HSA-6811558), which in our step centric method had a p-value of 0.000002 with *k* = 8 and *n* = 13. Via the gene list method, the pathway has a p-value of 0.218 with *k* = 8 and *n* = 115. Comparing this to the Reactome analysis tool, the result is similar (p-value of 0.67 with *k* = 7 and *n* = 118); the change in *k* is an artifact of annotation. We also analyzed the same data with DAVID [7], which had an adjusted p-value of 0.9, and PANTHER [4], which did not list it among results with FDR ≤ 0.05. Analysis with GSEA did not report this specific pathway among results but had lower FDR adjusted p-values of 0.27 and 0.55 for *Constitutive Signaling by Aberrant PI3K in* Cancer and *Negative Regulation of the PI3K AKT* network.

**Fig 4:**
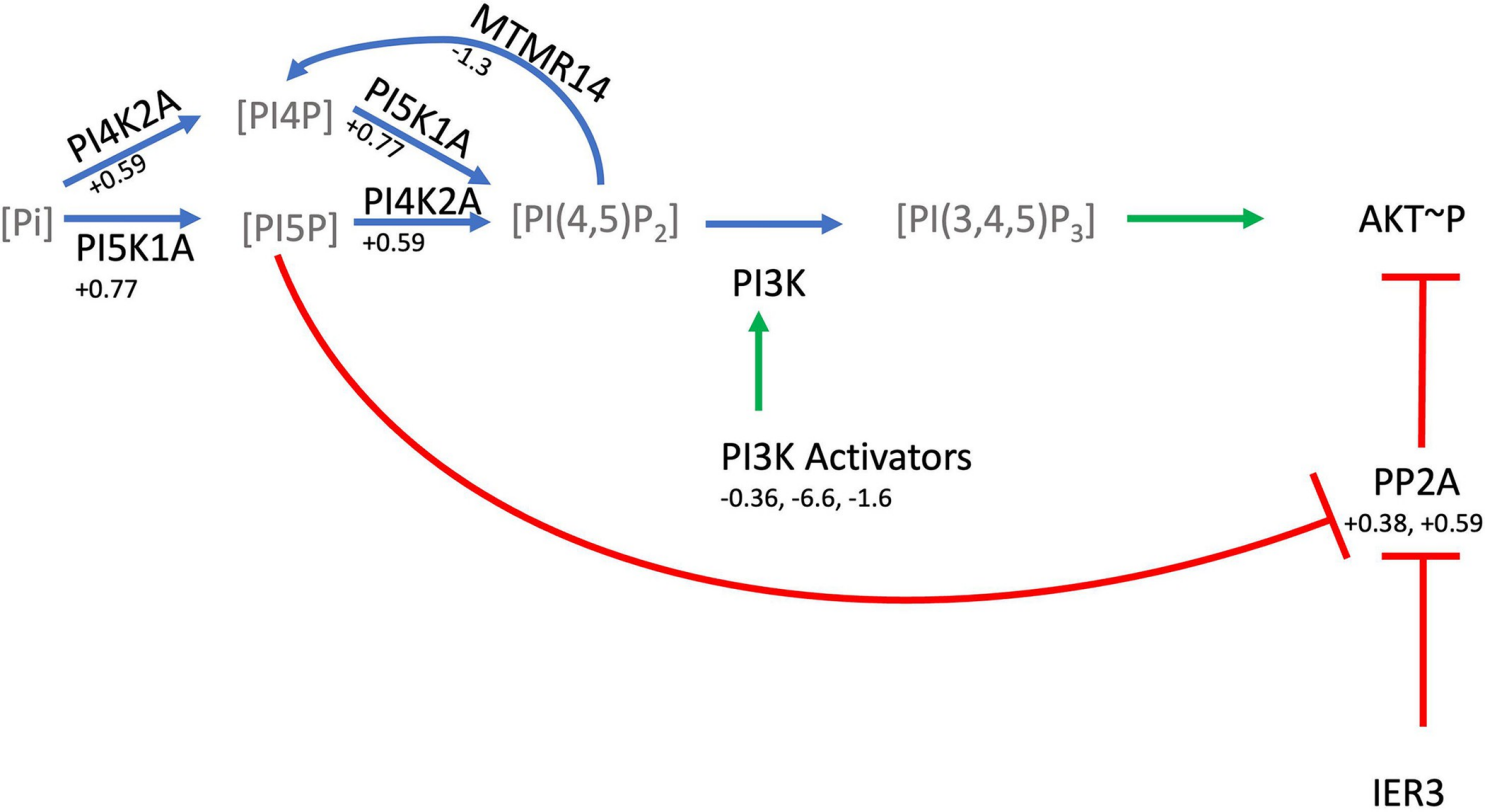
Regulation of PI3K signaling has multiple steps with perturbed protein expression. Pathway diagram derived from *PI5P*, *PP2A and IER3 Regulate PI3K/AKT Signaling* (R-I-6811558) with positive regulatory steps shown in green, negative regulatory steps shown in red, and phosphate addition/removal shown in blue. Log fold changes in proteins as measured by label-free proteomics in p97 shRNA knockdown versus control shown in small print [21]. Multiple fold changes may be listed due to multiple changes within sets or complexes.

Overlay of the fold changes of these proteins on the pathway is shown in Fig 4. A similar result is obtained from protein differential expression data with two p97 inhibitors also tested in the same study. While it is not apparent whether AKT signaling activity is up- or downregulated as a result, as PI3K seems to be downregulated but PIP2 concentration may be increased, the aim of enrichment analysis is hypothesis generation, and it is plausible that this signaling pathway may be meaningfully affected. While we found no literature specifically regarding PI3K/AKT signaling and p97 inhibitors, the involvement in PI3K/AKT signaling in cancers through enhancing cell survival and proliferation is well documented [30].

#### Dilated and arrhythmogenic cardiomyopathy may involve dysregulated complement

Lastly, the *Regulation of Complement Cascade* pathway (http://model.geneontology.org/R-HSA-977606) was significantly enriched in several of the datasets we examined from Reichart et al. [22] who performed single nucleus RNAseq on hearts from 61 patients with dilated or arrhythmogenic cardiomyopathy. We examined left ventricle arterial smooth muscle (SMC), fibroblasts (FB), and cardiomyoctes (CM) datasets from their supplemental data, and we observed that *Regulation of Complement Cascade* was enriched for several of the genotypes for both FBs and CMs. The genes and their roles in the complement cascade are shown below in Table 2.

**Table 2:** Log fold changes of genes involved in regulation of the complement pathway in the left ventricle [22] correspond to increased expression of pro-complement activation genes and decreased expression of inhibitors.

		Fibroblasts logFC					Arterial Smooth Muscle logFC					Cardiomyocyte LogFC				
Genotype		LMNA	PKP2	RBM20	TTn	PVneg	LMNA	PKP2	RBM20	TTn	PVneg	LMNA	PKP2	RBM20	TTn	PVneg
Number of patients		12	6	8	12	8	12	6	8	12	8	12	6	8	12	8
Gene	Role															
MASP1	Activates MASP2 in lectin pathway, cleaves C3	+1.8	+2.4	+1.1	+1.7											
CFD	Rate-limiting step of alt pathway	-1.8				-1.8	-2.8	-1.3	-1.3	-2.3	-1.7	-2.8			-2.4	
C1R	Part of classical pathway	-1.3	-2.1			-1.2	-1.1		-1.5	-1.4	-1.0	-2.3			-2.4	
C3	Initiates alt pathway and is part of classical + lectin pathway	-1.5		-2.0		-1.4	-1.8	-1.5	-1.5	-2.1	-1.6	-1.9		-2.5	-2.1	
ELANE	Activates C5	-2.4	-2.9	-1.8		-2.5										
CFH	Inhibits alt pathway						+1.0		+1.2		+1.5					
C1-INH	Inhibits classical pathway	-1.1			-1.1	-1.1	-1.0				-.9	-2.2				-2.3

Green shades indicates that the gene product promotes complement activation, while magenta corresponds to inhibition. Log_2_ fold changes are not shown if adjusted p-value > 0.05.

Surprisingly, the transcriptomic changes seem to suggest downregulation of several components this pathway, which is known to be upregulated in several cardiovascular diseases. See Discussion for further comments on this result in the context of published literature. While the downregulation of complement pathway genes is surprising, its involvement in dilated cardiomyopathy is another example of a plausible hypothesis recovered by our enrichment method. The GO-CAM model for complement activation has 11 step-enabling entities but the gene list has 117 members again due to set inflation (unadjusted p-value of 0.27 for 7/117 genes in fibroblasts in the LMNA genotype in our implementation; not reported as a significant result by PANTHER for 8/120 genes; unadjusted p-value of 0.70 for 9/156 entities in the Reactome Analysis tool) [4,10].

#### Gene and step enrichment analyses yield non-overlapping results

**Fig 5:**
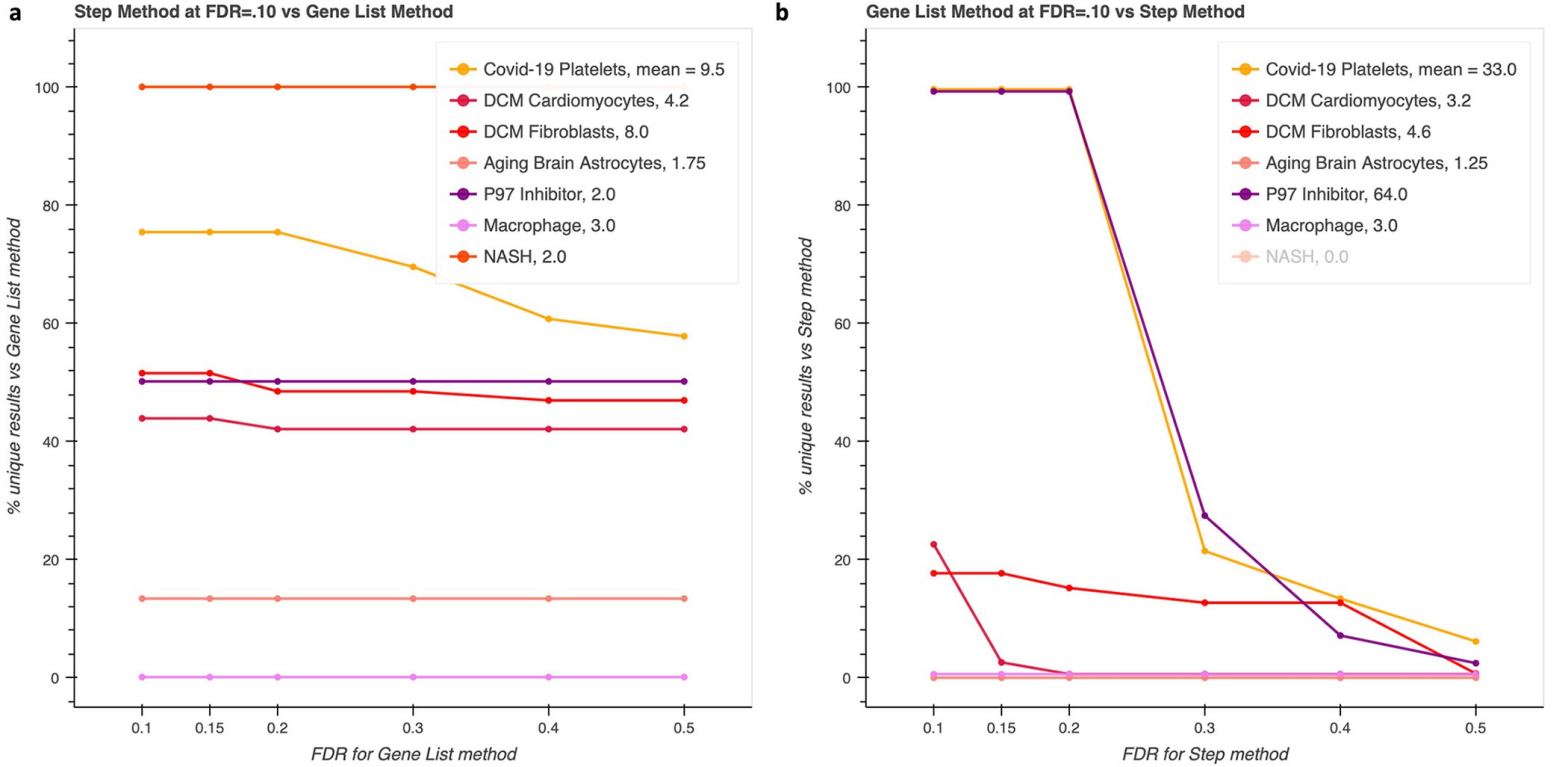
Comparing the percentage of unique (non-overlapping) results for test datasets. Step-centric enrichment shows increased sensitivity to pathway that are not recovered even with high false discovery rates (**a**), while traditional, gene list enrichment shows overall increased sensitivity, likely systematic due to a larger background size that is recovered by increasing the FDR for the step-centric method (**b**).

Next, we sought to understand the degree of divergence between the results across the datasets from those six papers in our analyses. Enrichment analysis does not produce probabilities that pathways are differentially regulated, so researchers need to examine pathway results by hand to decide to investigate further. Having already shown specific examples of plausibly differentially regulated pathways, we wanted to know if the unique results from our method could simply be produced by running standard enrichment analysis with a higher false discovery rate (FDR). If the missed pathways could be recaptured with a reasonable FDR, this approach could work, but if the FDR required is too great, then the number of false positives would be too high and there would be too many results for researchers to assess. Therefore, in addition to comparing the percentages of unique results at FDR = .10, we iteratively lowered the FDR threshold for the gene list method while keeping the FDR fixed at 0.1 for our method and vice versa (Fig 5). Our method generally produced higher percentages of unique results, but the standard method produced many more results in some cases, such as the platelet and p97 inhibitor datasets. At least two phenomena could contribute to this: the gene list method counts all genes while ours does not count multiple genes that belong to the same set, and the number of entities across all pathways in our database is ~4000 while there are ~5300 genes. This background size decrease of 25% can affect p-values by an order of magnitude or more, which can also interact with the Benjamini-Hochberg procedure and allow many results to be considered significant.

We aggregated the counts across the datasets and saw similar proportions of unique results versus total results at FDR = 0.1, but at FDR = 0.4, a much higher proportion of results are unique in our step-centric method (Table 3). Looking at the curves for the p97 inhibitor dataset and the platelet dataset, we see that most of these unique results were contributed by just those two datasets at 0.1, and there is a steep drop when the FDR is raised from 0.2 to 0.3, indicating that there was a systematic loss of sensitivity (such as due to the background size) rather than a targeted one for specific pathways (as is the case when very large sets inflate pathway sizes for gene list enrichment). We also include the same analysis but at FDR = 0.05 in S2 Fig, which is qualitatively the same.

**Table 3:** Comparing the number of unique (non-overlapping) results summed across test datasets.

	Gene List Method at FDR =					
	0.10	0.15	0.20	0.30	0.40	Total Number of Results
Step, FDR = 0.10	53	53	50	48	44	94
	**Step Method at FDR =**					
	0.10	0.15	0.20	0.30	0.40	Total Number of Results
**Gene List, FDR = 0.10**	136	134	132	46	23	177

The traditional gene list method produces more results at FDR = 0.1 (177 vs 94), but they can be recovered by raising the FDR on the step-centric method. Raising the FDR for the gene list method does not recover unique results from the step-centric method.

### The multivariate Fisher’s noncentral hypergeometric distribution

In gene list enrichment, the null hypothesis specifies that each gene has an equal probability of being sampled, while the method we proposed assumes that each entity has an equal probability of being sampled, which is equivalent to assuming each step in the pathway has an equal probability of perturbation if there are no complexes. To account for the number of genes in sets, we weighted the probability of sampling sets according to the number of genes within them using the multivariate Fisher’s noncentral hypergeometric distribution and the BiasedUrn package [31,32]. For example, a set of five genes is assigned a weight of 5, while an entity of just one gene has a weight of 1, corresponding to the relative odds of selecting these two entities with a single draw of a gene from the genome.

The hypergeometric distribution describes sampling balls from an urn (or genes from a genome) without replacement, where each ball has an equal probability of being drawn and the process continues until N balls have been drawn from the urn. The multivariate Fisher’s noncentral hypergeometric distribution is a generalization that allows different types of balls to be sampled with different probabilities. See Methods for further description.

**Fig 6:**
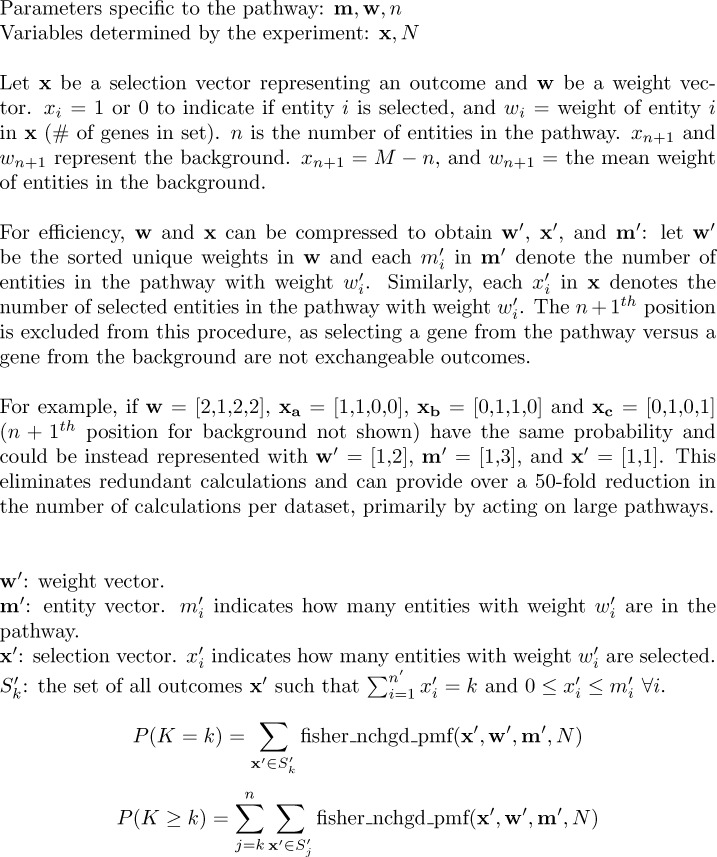
Computing a p-value. We use the multivariate Fisher’s noncentral hypergeometric distribution to obtain probabilities for outcomes and sum to obtain our probability mass function for the event P(K = k). We then sum over K ≥ k to obtain a p-value. fisher_nchgd_pmf is calculated using BiasedUrn [31]. Sorting and compressing the pathway vectors can greatly reduce the size of $S_{k}^{\prime }$. Across our test datasets, the median reduction of the number of calls per dataset to BiasedUrn by sorting and compressing the pathway vectors is 65 fold (the median of $(\sum_{pathways}\sum_{j=k}^{n}\left|S_{j}\right|)/(\sum_{pathways}\sum_{j=k}^{n}\left|S_{j}^{\prime }\right|)$).

To obtain a probability mass function for the distribution of our test statistic, *k*, we took advantage of its discrete nature and enumerated all possible outcomes where the number of entities sampled equals exactly *k*. We then computed the probability of each outcome where exactly *k* entities are sampled with the BiasedUrn package and sum to obtain the total probability of sampling exactly *k* entities. We repeated this for every valid value greater than *k*, and we sum to obtain a p-value. This procedure is detailed in Fig 6. It should be noted that sets may share members, which is discussed further in Limitations.

Alternately, we can simulate the traditional null hypothesis of independently selecting genes uniformly at random while using our proposed test statistic to avoid this problem of dependence while still including the notion of steps. See Methods for further discussion of drawbacks of simulation and our justification for using the multivariate Fisher’s noncentral hypergeometric distribution.

### Comparing results with Fisher’s noncentral hypergeometric distribution

**Fig 7:**
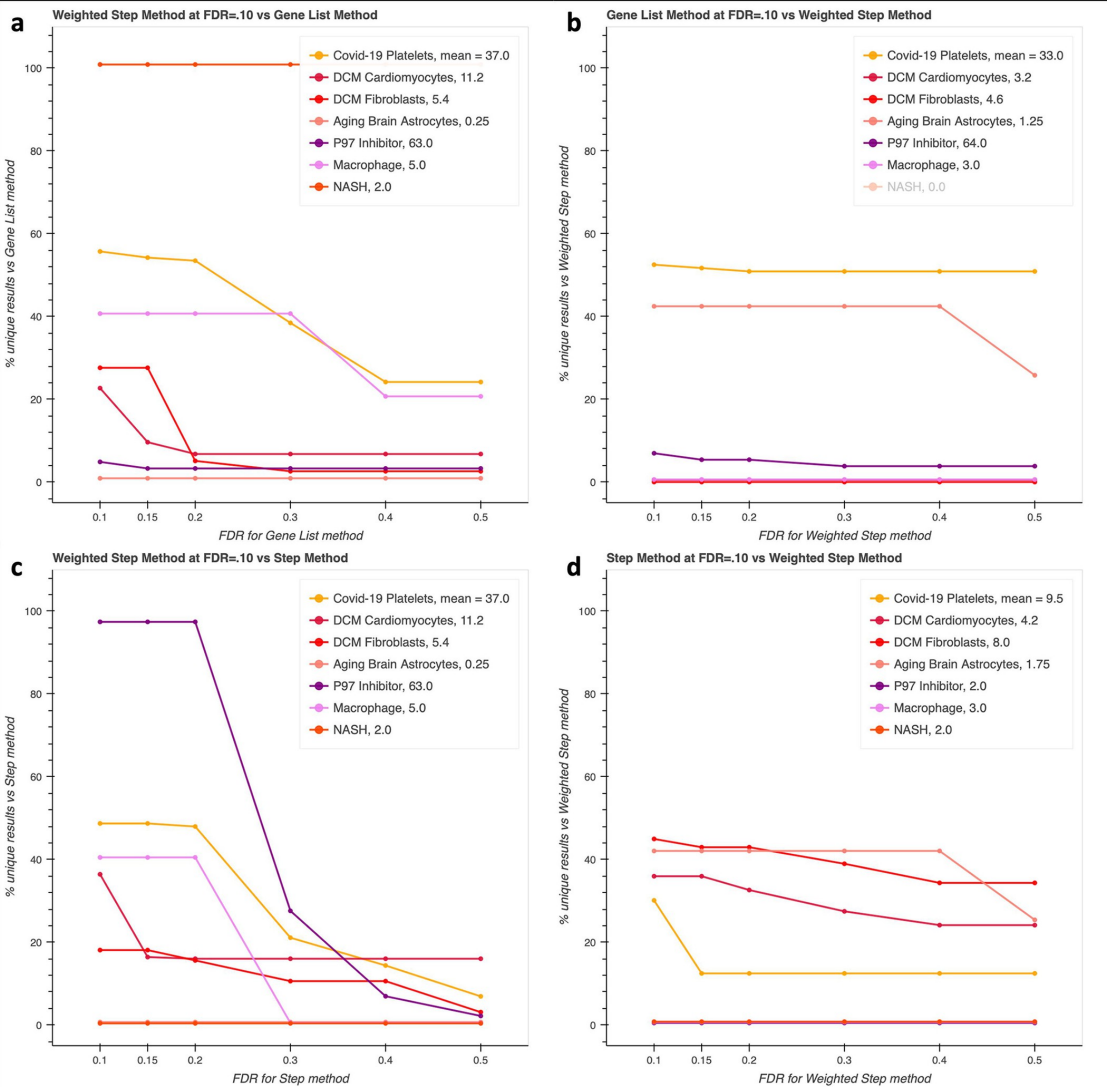
Pairwise comparisons with the multivariate Fisher’s noncentral hypergeometric distribution. Comparison of the results of weighted enrichment and the standard gene list method shows that the weighted method provides unique results (a) while having generally good agreement with the gene list method (b) compared to the unweighted step method (Fig 5B). Comparison between the weighted and unweighted methods shows that the weighted method produces more results than the unweighted step method (c) while still having relatively good capture rates of results from the unweighted method (d).

We next performed pairwise comparison on our test datasets between weighted enrichment done with Fisher’s noncentral hypergeometric distribution and the gene list method, as well as the step-centric method (Fig 7, Table 4). The weighted step enrichment and standard gene list methods had varying proportions of unique results, but they had generally high agreement, while the weighted step method also captured many of the enrichment results unique to the unweighted step method (compare Figs 5A with 7D). Fig 7B also shows that our weighted enrichment method recaptured many of the results that the standard gene method produced but the unweighted step method missed. 4 of 6 test datasets had less than 10% of results at FDR = 0.1 that are unique to the standard method. Note that one of the remaining two was Aging Brain Astrocytes, which had a denominator of 1.25 enriched pathways. We included it for transparency, but it has little value in interpreting. We also include the same analysis but at FDR = 0.05 in S3 Fig, which is qualitatively the same.

**Table 4:** Comparing the number of unique (non-overlapping) results summed across test datasets between the gene list, step, and weighted step methods.

	Gene List Method at FDR =					
	0.10	0.15	0.20	0.30	0.40	Total Number of Results
Weighted Step, FDR = 0.10	65	30	20	16	13	228
	**Weighted Step Method at FDR =**					
	0.10	0.15	0.20	0.30	0.40	Total Number of Results
**Gene List, FDR = 0.10**	14	12	11	10	10	177
	**Step Method at FDR =**					
	0.10	0.15	0.20	0.30	0.40	Total Number of Results
**Weighted Step, FDR = 0.10**	176	174	171	87	65	228
	**Weighted Step Method at FDR =**					
	0.10	0.15	0.20	0.30	0.40	Total Number of Results
**Step, FDR = 0.10**	42	35	34	31	27	94

The gene list and weighted step method have less overall divergence than the unweighted step method and the gene list method.

One might expect our unweighted step method’s results to be supersets of our weighted method’s results because all else equal, increasing the weight of sets in the pathway would increase p-values. However, p-values from the unweighted method are increased by the decreased background probability mass compared to the gene list method as the number of genes in the background exceeds the number of entities. This systematically decreases sensitivity, which is ameliorated in the weighted step method because entities in the background are weighted as well. Weighted step-centric enrichment and unweighted step-centric enrichment showed more divergence in results at typical FDRs but with gradual recapture as the FDR drops for the weighted method (Fig 7; Table 4). Lastly, we note that of the case studies shown earlier, the collagen synthesis pathway and PI3K regulation were still enriched with our weighted method, and the complement pathway was enriched in some genotypes with the weighted method but was past the cutoff for others.

## Discussion

We introduce enrichment analysis on steps in pathways and integrate the notion of “sets,” which we have not seen elsewhere in analysis tools but is commonly used in pathway databases. Our motivation is to perform an enrichment analysis that better reflects the biology of pathways and distinguishes between protein complexes and sets. We show that this can lead to significantly different results from enrichment with greater sensitivity towards pathways suffering from gene count inflation due to sets, which is critical for researchers looking to use enrichment analysis to plan follow up experiments. We do this by reanalyzing data from several papers- both characterizing the number of differential results in aggregate and discussing specific examples of pathways that are plausibly differentially active. Some of these examples have previously been shown in the literature, such as decreased synthesis of very long chain fatty acids by platelets during SARS-CoV-2 infection, and some are novel, such as collagen production by platelets during SARS-CoV-2 infection. We then utilize Fisher’s noncentral hypergeometric distribution to weight sets according to the number of genes within them to partially preserve an assumption in the null hypothesis of gene list enrichment analysis.

We compare the results obtained with the hypergeometric distribution between our step-centric method that uses a list of step-enabling entities and the standard method using a gene list, and we discover that up to half or more of results from each method with FDR = .1 can be unique. Furthermore, increasing the FDR for the opposing method only partially recaptures those non-overlapping results. Note that we did not evaluate enrichment results only produced by the standard gene-list method, because it is already widely used and accepted. We believe that results unique to standard enrichment analysis are not necessarily irrelevant, as there may be cases where many members of a set are upregulated in a biologically meaningful manner, which our method may miss. Also, the larger background size of the traditional gene-list method systematically decreases p-values, which could lead to reporting many more results as significant, as for the P97 dataset when comparing our unweighted step enrichment procedure with the traditional gene-list method in Fig 5B. Likewise, we found that the results of our case study pathways were not significantly enriched in the popular GSEA algorithm, but we did not evaluate the credibility of results unique to GSEA as it is also well accepted [12].

One-way, pairwise comparisons with weighted enrichment analysis on steps using the multivariate Fisher’s noncentral hypergeometric distribution showed it had the least divergence from the standard method but still provided unique results. Unique results from this method could be evaluated alongside results from standard gene-list enrichment. However, it unclear if weighting is always necessary. We use enrichment to generate viable hypotheses that can be evaluated based on literature and investigated further as opposed to providing accurate p-values with which to reject a null hypothesis that we already know is incorrect, as cells do not select genes independently with uniform probability due to intricacies of gene regulation that are unrelated to regulation of pathway activities. More importantly, sets sometimes indicate tissue-specific expression, in which case neither weighting nor the standard gene list method is biologically meaningful, because cells of that type do not actually have access to sampling all members of the set. Even when sets do not indicate gene-expression switches between tissues, weighing sets by their gene content might not be appropriate; the collagen pathway was primarily inflated by a set corresponding to 44 procollagen genes, yet these encode different types of collagen proteins and only two need to be expressed, let alone differentially expressed, to produce collagen. What is necessary, however, is deciding whether this method is appropriate for a given dataset. Our core idea is that an individual cell (or population of cells employing the same regulatory strategy to respond to a condition) is unlikely to need to significantly perturb expression of every known member of a set in order to regulate a step in a pathway. Some members may not be expressed and therefore cannot be downregulated, are lowly expressed for other functions but are not relied upon by that cell type for that pathway, or are not “accessible” to upregulate because that cell does not express those genes under any normal conditions. Given this idea, we believe it is appropriate to use our method on single cell data and bulk data corresponding to a single cell type, while using our method on a heterogenous source (i.e. mutation data pooled from many patients’ tumors) could lead to spurious results. It may be possible to use our method on bulk-RNA seq from a whole tissue, but care should be taken to interpret each result based on whether most cells in the sample likely employed the same regulatory strategy for the pathways of interest. If a dataset meets the criteria above, we believe using our method alongside standard enrichment analysis is beneficial, but if not, we recommend the standard method.

Assuming a given dataset is an appropriate choice for our enrichment methods to do a comparison, p-values can differ by orders of magnitude between the various algorithms, but even within the same method, p-values are sensitive to relatively small changes in background size and changes to cutoffs for generating differentially expressed gene lists from the experiments themselves. As mentioned above, the p-values themselves are not the focus of enrichment analysis tools. Enrichment analysis aims to provide researchers with hypotheses to follow up on based on the results of their experiments. As a hypothesis generating tool, ideal enrichment analysis would have a high sensitivity and enable us to uncover novel biology. So long as there are not so many results that it becomes impractical for a researcher to consider each one critically when deciding to investigate further, it is perhaps better to err on the side of false positives than false negatives. This bias motivates us to formulate an enrichment question more representative of the biology at hand.

### Discussion of pathways in case studies

#### Collagen I production by platelets

Collagen is known to contribute to platelet aggregation and clot formation, but platelets have not historically been considered sources of collagen. However, there is recent support in the literature for this possibility. Collagen modifying enzymes have been shown to be expressed in platelets, although their activity against mature collagen triple helices in the extracellular space was not observed, suggesting that platelets do not modify extracellular collagen triple helices produced by other cells [33]. Recently, collagen I synthesis in activated platelets has been observed [28] in platelets derived from patients with polycystic ovarian syndrome (PCOS), which is known to be associated with increased platelet aggregation. These platelets were shown to contain higher levels of hydroxyproline than platelets from healthy donors, COL1A1 mRNA was detected, and collagen was detected at higher levels in platelets from PCOS patients. To demonstrate that the detected collagen originated from within the platelets themselves as opposed to contamination, the authors showed that collagen levels increased when platelets were activated with thrombin and coated the surfaces of the platelets, while in the absence of activation, collagen was detected only after membrane permeabilization with Triton X-100. They note that procollagen mRNA was not significantly different between the platelets of healthy donors and the PCOS patients, yet collagen levels were different, speculating that this may be due to increased activity of enzymes in the collagen synthesis and modification pathway.

Manne et al. [20] did not assay collagen levels, and mRNA levels were not increased for collagen I, yet mRNA levels for enzymes at every step of the pathway were increased, consistent with the findings of Kyselova et al. [28] that collagen levels and markers of collagen production were increased despite no increase in collagen mRNA.

#### Dilated and arrhythmogenic cardiomyopathy

The role of complement in dilated cardiomyopathy is unclear, but complement activation has been implicated in various cardiovascular and cardiac diseases, and complement activation has been shown specifically to play a role in congestive heart failure [34,35]. Endogenous production of complement components following myocardial infarction has been shown [36], and C3 and CFD have been shown to play a role in cardiac remodeling in right heart failure in mice, where C3 and CFD deletion ameliorated heart failure [37]. It should be noted, however, that the remodeling in right heart failure due to pulmonary hypertension typically results in hypertrophy, not dilation.

Curiously, the changes in gene expression in Reichart et al. [22] all suggest downregulation of the complement cascade at the initial steps of the alternative (CFD, CFH, C3), classical (C1R), and lectin (MASP1) pathways as well as downstream (C3, ELANE), except for increased MASP1 in fibroblasts and consistently decreased C1 Inhibitor in the LMNA and Pathogenic Variant negative (PVneg) genotype for all three cell types. While survivorship bias is a possibility (i.e. cells that did not downregulate complement activation were destroyed), complement has roles beyond cell lysis; intracellular complement activation and local complement secretion by cells is beginning to be understood as having important roles for regulating cell activities, including in endothelial cells and fibroblasts [38]. Ito et al. [37], who studied RV heart failure, also observed that complement system genes are increased in healthy controls in the right versus left ventricle, and deletion of C3 did not ameliorate LV failure due to aortic constriction in mice [37], raising the possibility that complement pathway plays different roles in the left and right ventricles. Its downregulation may play a protective role in dilated cardiomyopathy, explaining why C3 deletion had no effect on LV in Ito et al., or it might be involved in the pathogenesis of dilated cardiomyopathy through dysregulation of currently unknown cellular functions. Proteomics of plasma have shown that decreased C3 levels are associated with poorer survival in congestive heart failure [39]. Alternatively, it is possible that complement genes are downregulated due to medications; however, primary treatment for DCM includes angiotensin-converting enzyme inhibitors and beta blockers, neither of which have been shown to decrease complement.

### Limitations

#### Sets count once towards overlap

In the hypergeometric test (or Fisher’s exact test), the actual fold change of a gene is not accounted for. Genes are added to the list if they pass some cutoff criteria. In keeping with that idea, we treat selection of two members of a set the same as selection of one member. If their activities contribute additively towards the total activity of the step, then this is analogous. However, there are examples of interactive behavior between sets, even a decrease in activity when multiple members of a set are upregulated. In BMP signaling, there are many ligands that can interact with many receptors, and in some cases, increased concentration of multiple ligands can actually decrease BMP signaling due to binding differences in affinities and reaction kinetics [19]. Ideally, the interactions between members of sets would be characterized and accounted for. Furthermore, sets sometimes denote genes with tissue-specific expression, as in glycolysis. Not all members of a set are available for given cell types to up or downregulate, so upregulation of just one or two members of the set might actually constitute upregulation of all members of the set that are actually expressed in that cell. Additionally, in the case of downregulation in response to a treatment, only one member of a set may be expressed prior to treatment, and again, not all members of the set are available to be selected for regulation. We currently do not distinguish between these cases and err on the side of leniency.

#### Positive dependence in the weighted distribution

We note that this model suffers from the problem of overlaps between sets. This creates a relatively weak dependence between sets and a strong correlation between genes as solo entities and any sets that contain them. This violates the assumption of independence in the distribution. Typically, genes will not appear in more than one step within a single pathway; they are more likely to appear in multiple entities across pathways due to artifacts of annotation or genuine biology, but they are in the background relative to the pathway being tested. In some cases, some enzymes can catalyze multiple steps in pathways, and it is possible to “double count” genes in this manner if an enzyme can uniquely catalyze one step but is one of several in another step, as seen in the collagen synthesis example.

## Methods

### The multivariate Fisher’s noncentral hypergeometric distribution

#### Fisher’s distribution is more appropriate than Wallenius’s for enrichment

Fisher’s noncentral hypergeometric distribution and Wallenius’s noncentral hypergeometric distribution both describe sampling processes where the balls are not drawn with equal probability; they are weighted [31,32]. However, Fisher modeled a process in which all balls were sampled independently, and the total number of balls drawn is itself a random variable (i.e., all at once), while Wallenius modeled a process in which balls were sampled one by one until the desired number of balls were drawn. In that scenario, the order of drawing the balls matters- drawing a ball with very high weight first and removing it from the urn increases the chance of drawing the remaining balls, whereas drawing a ball with low weight first does not substantially affect the probability of drawing the heavy ball next. To maintain independence, Fisher’s distribution treats the number of balls drawn in the experiment as a random variable itself, and then once the experiment has been performed and *N* is known, the multivariate distribution of balls drawn of each type can be conditioned on *N*. This aligns with the process of differential expression experiments, where we do not know the number of differentially expressed genes prior to the experiment, and there is no reason why a cell would only perturb a fixed number of genes. Therefore, we use Fisher’s noncentral hypergeometric distribution to model outcomes of the experiment (specific arrangements of gene perturbations) and sum the probabilities of those outcomes to obtain p-values for our events (the number of perturbed genes in the pathway).

#### Fisher’s distribution over simulation the traditional null hypothesis with a new test statistic

Let *k* be the number of entities with at least one gene member selected. A cell randomly selects genes independently with uniform probability. *N* genes are selected without replacement from *M* total. Assuming no entities share overlapping genes within a pathway, this shares the same generative process as the traditional, gene-list method, but it has a different test statistic. Repeated sampling of an entity does not count towards *k*. Assuming a gene-first generative model, simulating uniform probability, independent gene selection will give accurate p-values with respect to that null hypothesis without problems of dependence. However, this suffers from two drawbacks. First, we believe it is more biologically relevant to have a generative model where a cell selects steps to regulate without replacement and the genes merely reflect that. Second, simulation is slow, as multiple testing correction necessitates that we have accurate estimates of p-values several orders of magnitude smaller than 0.05. While this can be pre-computed for many values of *N* and then interpolated, it would need to be recomputed for custom backgrounds that a user may specify for their experiment, and simulation on the fly would be intractable.

#### Representing the background to select from

In the Results, we discuss *k*, the overlap and detail our procedure for calculating p-values with weighted entities in Fig 6. Here, we discuss the parameters *M* and *N* for the hypergeometric distribution and how they factor into our use of the multivariate Fisher’s noncentral hypergeometric distribution. There is no *M* for this distribution, although it exists implicitly. The multivariate Fisher’s noncentral hypergeometric distribution is parameterized by ***m***, ***w***, ***x***, and *N*. Let there be *c* colors of balls in the urn. ***m*** and ***w*** are vectors of length *c*, respectively, where *m*_*i*_ is the number of balls of color *i* and *w*_***i***_ denotes the weight for balls of color *i*. ***x*** corresponds to an outcome in the sample space. It is a vector of observed counts for the sample, and *x*_*i*_ is the number of balls sampled of color *i*. *N* is the total number of balls sampled.

In our problem, let there be *n* entities in the pathway. We can group them according to their weights to produce a vector ***m*** for our pathway, where *m*_*i*_ indicates the number of entities with weight *w*_*i*_, which is either 1 for proteins or is the cardinality of the set represented by element *i*. ***x*** takes the same form, and $\sum_{i=1}^{len(x)}\;x_{i}=k$ is the number of entities selected from the pathway in the outcome represented by ***x***. Each element *x*_*i*_ is subject to the constraint *x*_*i*_≤*m*_*i*_ (i.e. if there are only 3 sets in the pathway with 5 genes in them, we cannot observe an event ***x*** where we have 4 entities of weight 5).

However, these vectors only describe the pathway and lack a description for the background against which entities are selected, previously represented by *M*. We added an (*n+*1)^th^ element to each vector to represent the entities outside of the pathway, where *w*_*n*+1_ is the mean weight for the remaining entities in the background , and *m*_*n*+1_ = *M*−*n*. An exact representation would add more than 1 element to represent the pathway; after constructing ***m***_***pathway***_ and ***w***_***pathway***_, the same procedure could be used to construct ***m***_***background***_ and ***w***_***background***_ vectors for the background (representing each entity not in the pathway and their individual weights), and ***x***_***background***_ would be constrained by summing to *N*−*k*. We did not do this because representing every entity in the background produces a much larger sample space and would be not computationally feasible. Instead, we approximated the background with entities of the mean weight (rounding to 2 decimal places) to preserve the odds of entities within the pathway vs not in the pathway. Approximating the background at a less coarse resolution with two groups of entities by sorting the background entities by weight, splitting at the median into upper and lower halves, and approximating each group with its mean weight incurred a two order of magnitude increase in runtime while making a negligible difference in p-values. For the nonalcoholic steatohepatitis dataset [24], the two significantly enriched pathways were the same, and their p-values decreased very slightly from 2.68×10^−7^ to 2.71×10^−7^ and 1.29×10^−4^ to 1.30×10^−4^; for the platelets dataset [20], the 67 enriched pathways were the same and their p-values were reduced by a median of 11% with 2.5th and 97.5^th^ percentile reductions of 1% and 24%). Therefore, we approximated the entire background as one group with its mean weight.

Implementation was done with python v3.10.9, R v4.2.2, rpy-2 v3.5.9, SciPy v1.9.3, and BiasedUrn v2.0.10. A full description of the Conda environment is available at our GitHub repository in environment.yml.

### Pathway data collection

GO-CAM models used in this study were from the 2022-07-01 release (doi:10.5281/zenodo.6799722). All GO-CAM models in production state were filtered to select those with at least three molecular functions (steps) linked by two causal edges (142 pathway models). We did this because these GO-CAMs are more likely to represent a process or pathway corresponding to a GO Biological Process than other, partial models, which may reflect curation practice for entering annotations into the GO. Then, GO-CAM models imported from Reactome, which are not yet officially released in GO-CAM form, were selected as well (1087 pathway models). These were generated from Reactome using the methods described in Good et al. [16]. As a note, GO curators do not currently curate sets, but they are allowed in the GO-CAMs that are created by direct conversion from Reactome. Entities enabling the molecular functions in these models were extracted using the “enabled by” relation (RO:0002333). All pathways were annotated as human or mouse; genes in mouse pathways were mapped to human orthologs using SimpleMine (v5.0.0) at http://mangolassi.caltech.edu/~azurebrd/cgi-bin/forms/agr_simplemine.cgi. Reactome entities were mapped to UniProt accession IDs using the Reacto ontology (v1.0) at http://www.geneontology.org/ontology/extensions/reacto.owl and all protein members of complexes and sets were retained while ions and non-protein chemical entities were discarded.

Some complexes and sets contain subsets and/or subcomplexes within them. Generally, these can be recursively simplified by taking complexes to mean an AND joining of their members and sets to entail an OR relationship (Figs 2, 1.4a; S1). 100 random examples of Reactome entities were checked manually after recursive conversion to entities with UniProt accession numbers. 2 were represented logically inconsistently and failed in the following way: let Set 1 be a set of 3 complexes C1, C2, and C3. C1 = P1 & Pa; C2 = P1 & Pb; C3 = P1 & Pc & Pd. Then S1 = P1 & {Pa or Pb or Pc or Pd}. An input of [P1, Pc] would count as the entirety of S1 being perturbed.

### Test data sources

See Table 5 below.

**Table 5:** Test datasets.

Dataset	Source	Paper
Colon cancer cell line response to p97 shRNA	Supp 1: shRNA DE	Wang et al., 2022 [21]
Platelets in Covid-19	Supp 3	Manne et al., 2020 [20]
Left Ventricle Cardiomyocytes	Supp 7, 8: all genotypes and PVneg	Reichart et al., 2022 [22]
Left Ventricle Fibroblasts	Supp 16, 17: all genotypes and PVneg	Reichart et al., 2022 [22]
Arterial Smooth Muscle	Supp 23, 24: all genotypes and PV neg	Reichart et al., 2022 [22]
Astrocytes in Aging Mouse Brain	Supp Table 5, CB and HTH	Boisvert et al., 2018 [23]
Macrophage activation in vitro	Table S1	Orecchioni et al., 2021 [25]
Nonalcoholic steatohepatitis	Table S2	Govaere et al., 2023 [24]

Gene lists were filtered for adjusted p-value < 0.05 and log fold change > 1 or < -1 unless done otherwise in their respective papers. Gene lists were split by up and downregulated genes for Manne et al. [20] and Boisvert et al. [23] and by genotype in Reichart et al. [22] and brain region in Boisvert et al. [23].

### Enrichment with PANTHER, REACTOME, GSEA, and DAVID

Differentially expressed genes or protein were analyzed with PANTHER’s Gene List Analysis tool by selecting “statistical overrepresentation test,” “Reactome pathways,” “Homo sapiens genes,” “Fisher’s Exact,” and “Calculate False Discovery Rate.” The same list of proteins were queried at DAVID by selecting “Functional Annotation Tool,” and “REACTOME_PATHWAY” and at Reactome by selecting “Analysis Tools” on the home page and the default “Project to Human” option on step 2. GSEAPreranked was performed with GenePattern using the reported log fold changes, “c2.cp.reactome.v2023.2.Hs.symbols.gmt” for the gene sets database, the default 1000 permutations, and “No_Collapse” [12,27]. GSEA was not performed on the dilated and arrythmogenic cardiomyopathy dataset as Reichart et al., reported log fold changes only for significantly differentially expressed genes [22].

## Supporting information

S1 Fig Simplifying a set of complexes. A set of complexes with nested sets is recursively simplified into a complex of two simple sets. All sets and complexes are processed in this manner to remove nesting.
(TIFF)

S2 Fig Comparing the percentage of unique (non-overlapping) results for test datasets at FDR = 0.05. Compared to Fig 5, which performed the comparison at FDR = 0.1, the results are qualitatively similar.
(TIFF)

S3 Fig Comparing the percentage of unique (non-overlapping) results for test datasets at FDR = 0.05. Compared to Fig 7, which performed the comparison at FDR = 0.1, the results are qualitatively similar.
(TIFF)

S1 File Documentation and example. (PDF)

S2 File Supplemental Text. Enrichment analysis results with FDR = 0.1 used to produce Figs 5 and 7.
(PDF)
