# Supplementary figures and images for: Enrichment on steps, not genes, improves inference of differentially expressed pathways
Source: PLoS Comput Biol. 2024 Mar 25;20(3):e1011968. doi: 10.1371/journal.pcbi.1011968 (PMC10994554; doi:10.1371/journal.pcbi.1011968)

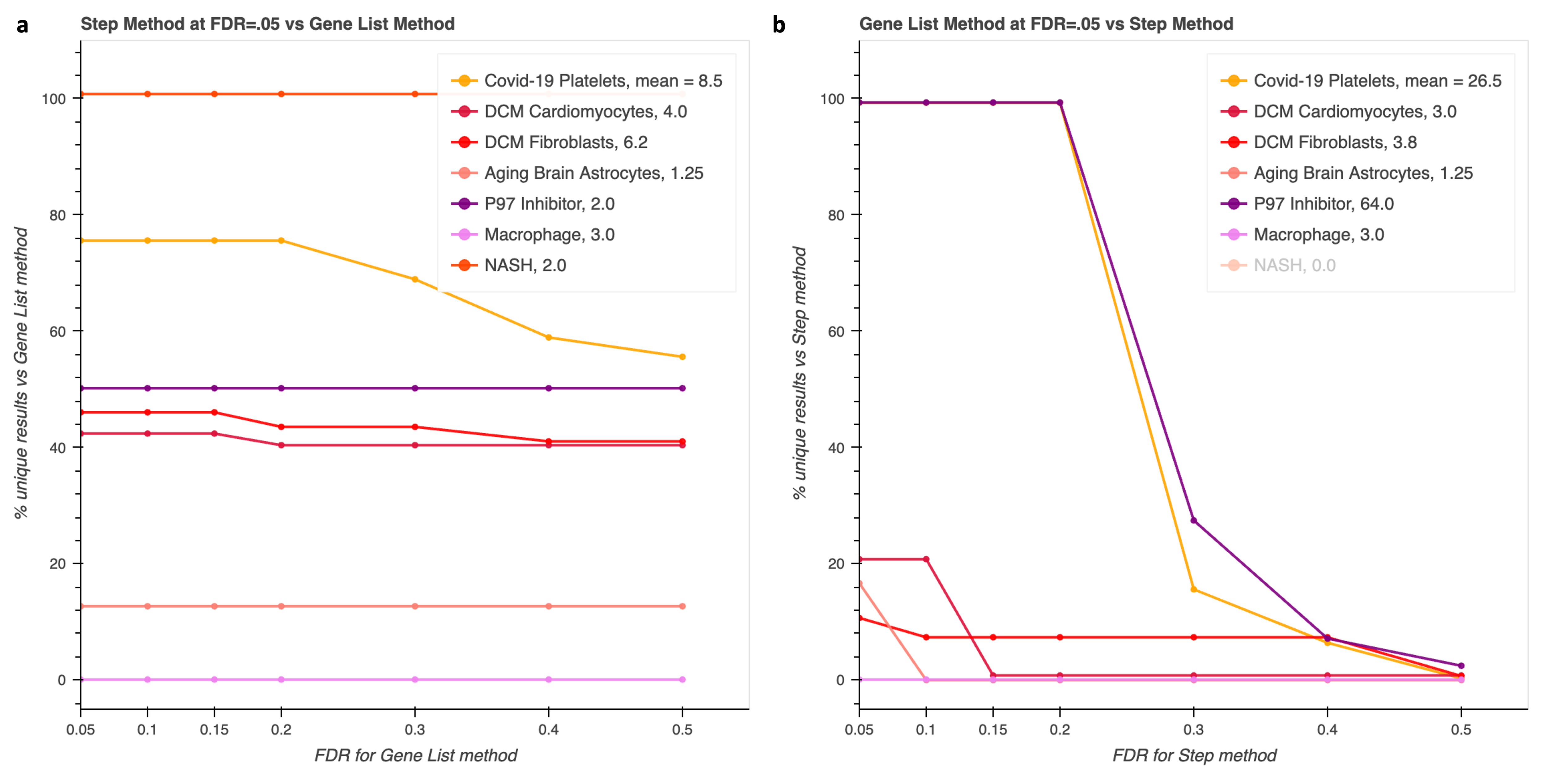

Supplement: S2 Fig — Compared to Fig 5, which performed the comparison at FDR = 0.1, the results are qualitatively similar. (TIFF) [file pcbi.1011968.s002.tiff]

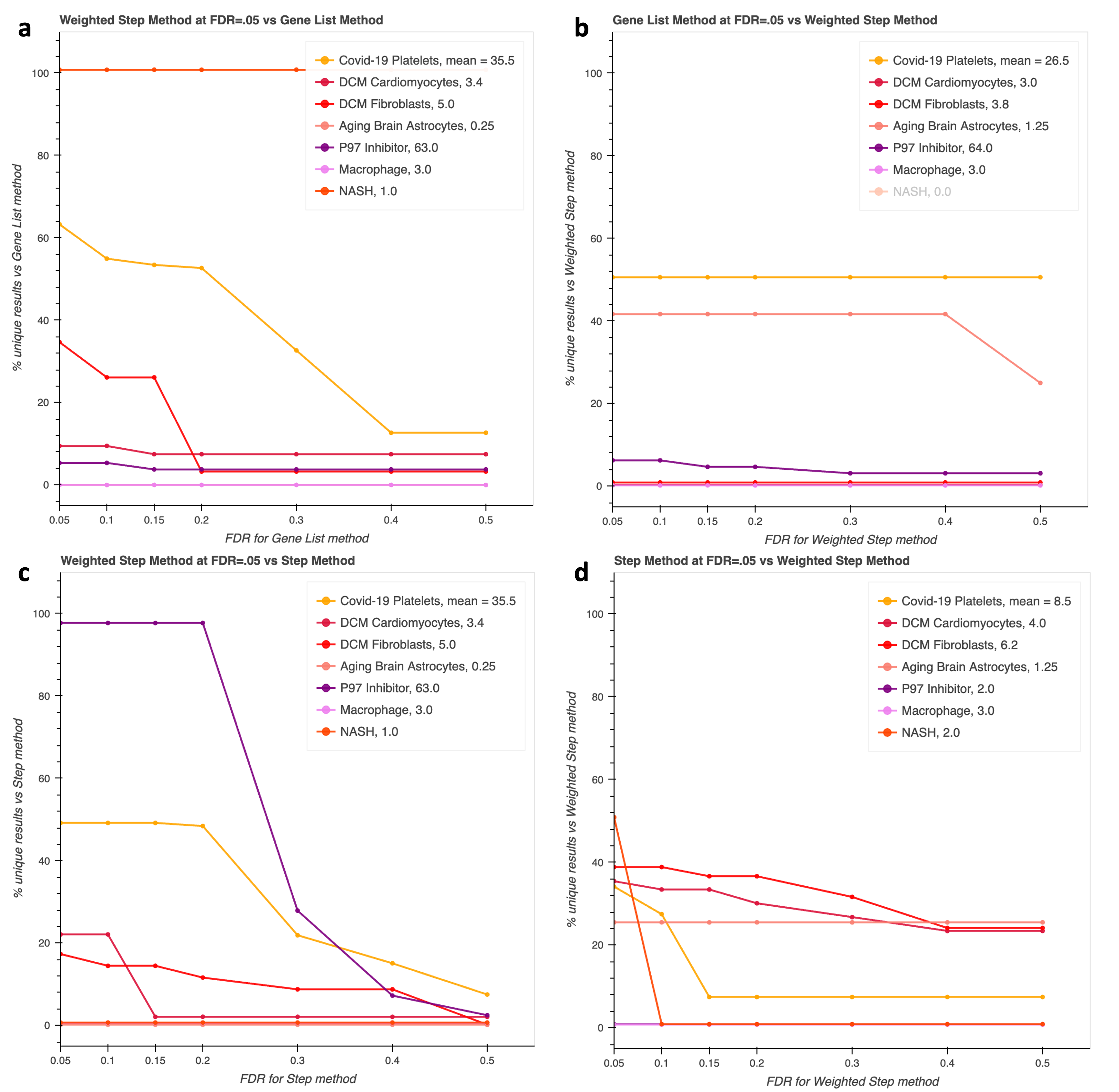

Supplement: S3 Fig — Compared to Fig 7, which performed the comparison at FDR = 0.1, the results are qualitatively similar. (TIFF) [file pcbi.1011968.s003.tiff]
