# Supplementary material for: Enrichment on steps, not genes, improves inference of differentially expressed pathways
Source: PLoS Comput Biol. 2024 Mar 25;20(3):e1011968. doi: 10.1371/journal.pcbi.1011968 (PMC10994554; doi:10.1371/journal.pcbi.1011968)
Supplement: S1 File — (PDF) [file pcbi.1011968.s004.pdf]

In [1]:
```
import pandas as pd
import numpy as np
import sys
import os
sys.path.append('../dev')

import utils
import enrich

pd.options.display.max_colwidth = 300
pd.set_option('display.max_rows', None)

!date
```

```
Wed Feb 14 12:27:46 PST 2024
```

Documentation and Example

To do an enrichment of your own, you can substitute your file name and path into the following line:

In [2]:
```
file_name = 'platelets_up.csv'
file_path = '../test_data/processed/'
```

Alternatively, you can specify both the path and the file name as in: file_name = '../test_data/processed/platelets_up.csv' *but then file_path must be omitted as an argument (or you can set* file_path = '' *)*

The file must be a csv with one gene or protein ID per line.

Permitted IDs types are:

- UniProtKB accession numbers https://www.uniprot.org/help/accession_numbers
- HGNC symbols (the names not the IDs. ie GAPDH not HGNC:4141 https://www.genenames.org/data/gene-symbol-report/#!/hgnc_id/HGNC:4141)
- MGI gene names (mouse IDs will be cross referenced with human IDs)

For other ID types, you can convert them here: http://mangolassi.caltech.edu/~azurebrd/cgi-bin/forms/agr_simplemine.cgi

Below is the function **enrich_wrapper()**, which should be called to perform enrichment given a file name, gene ID type, enrichment method, and false discovery rate. The first and second arguments, 'filename' and 'id_type', are required. The rest are optional, but 'method' specifies the enrichment method to be used (standard hypergeometric, unweighted step enrichment, or weighted step enrichment).

def enrich_wrapper(filename, id_type, method = 'set', return_all = False, FDR=.05,fpath= '../test_data', display_gene_symbol = True)

to specify enrichment method: default 'set'

- method = 'set' indicates unweighted, step-centric hypergeometric enrichment analysis. Sets and genes are weighted equally with a weight of 1.
- method = 'ncHGT' indicates weighted, step-centric enrichment analysis using Fisher's noncentral hypergeometric distribution and the BiasedUrn package.
- method = 'standard' indicates gene-list hypergeometric enrichment analysis. We implemented it here as results can vary from one tool to another based on the backend database of pathways used.

return_all: default False

- if false, only returns the dataframe displaying results.
- if true: returns (gene_list, filtered_out_genes, filtered_list, setID2members_input_uni, setID2members_input, df_display). User may want to know which of their input genes were filtered out as well as how the IDs were mapped, as uniprot IDs can sometimes map to more than one HGNC gene symbol

display_gene_symbol: default True

- if true, display HGNC symbols on output regardless of input ID type
- if false, display output using the same ID type as the input

Example: Platelets in SARS-CoV-2

Below, we will run enrichment analysis using all three methods at false discovery rates of 0.05. Then we will repeat the first enrichment but with a false discovery rate of 0.1.

Kanth manne et al., 2020

https://ashpublications.org/blood/article/136/11/1317/461106/Platelet-gene-expression-and-function-in-patients

RNAseq in platelets from patients with covid-19 vs healthy donors. There were 6 ICU and 4 nonICU patients.

Enrichment analsis with the unweighted set method and FDR = 0.05

In [3]:
```
results_set = enrich.enrich_wrapper(file_name,'Gene Symbol',method='set',FDR = 0.05,fpath = file_path)
results_set
```

```
100%|██████████████████████████████| 482/482 [00:00<00:00, 15814.31it/s]
Analysis run on 423 entities from 365 out of 1172 input genes
```

Out[3]:

	title	pval (uncorrected)	# entities in list	#entities in model	shared entities in gocam	url
0	Collagen biosynthesis and modifying enzymes - Reactome	8.974459e-09	10	12	[PPIB, P3H1, PLOD3, P4HB, set:Prolyl 3-hydroxylases, set:COLGALT1,COLGALT2, set:4-Hyp collagen propeptides, set:Procollagen N-proteinases, set:Procollagen C-proteinases, set:Lysyl hydroxylases]	http://model.geneontology.org/R-HSA-1650814
1	Synthesis of PE - Reactome	4.973450e-05	8	15	[PTDSS2, PCYT2, PISD, PHOSPHO1, set:LPIN, set:CHK/ETNK, set:PNPLA2/3, set:AGPAT]	http://model.geneontology.org/R-HSA-1483213

Repeat with standard enrichment:

In [4]:
```
results_standard = enrich.enrich_wrapper(file_name,'Gene Symbol',method='standard',FDR = 0.05,fpath = file_path)
results_standard
```

```
100%|██████████████████████████████| 482/482 [00:00<00:00, 17972.34it/s]
```

Out[4]:

	title	pval (uncorrected)	# entities in list	#entities in model	shared entities in gocam	url
0	ER-Phagosome pathway - Reactome	0.000045	13	52	[SEC61A1, PSMD13, PSMA5, PSMD11, SEC61B, PSMA7, PSME2, PSMD4, PSMB1, PSMB7, PSMD8, PSMB5, PSMB6]	http://model.geneontology.org/R-HSA-1236974
1	Hedgehog ligand biogenesis - Reactome	0.000045	13	52	[PSMD13, PSMA5, PSMD11, PSMA7, PSME2, PSMD4, PSMB1, PSMB7, PSMD8, PSMB5, P4HB, PSMB6, SYVN1]	http://model.geneontology.org/R-HSA-5358346
2	Regulation of APC/C activators between G1/S and early anaphase - Reactome	0.000069	13	54	[PSMD13, PSMA5, PSMD11, PSMA7, PSME2, PSMD4, PSMB1, CDC25B, PSMB7, CDK1, PSMD8, PSMB5, PSMB6]	http://model.geneontology.org/R-HSA-176408
3	Conversion from APC/C:Cdc20 to APC/C:Cdh1 in late anaphase - Reactome	0.000111	12	49	[PSMD13, PSMA5, PSMD11, PSMA7, PSME2, PSMD4, PSMB1, PSMB7, CDK1, PSMD8, PSMB5, PSMB6]	http://model.geneontology.org/R-HSA-176407
4	Neddylation - Reactome	0.000113	14	64	[UBE2M, PSMD13, PSMA5, PSMD11, PSMA7, PSME2, PSMD4, PSMB1, CUL9, PSMB7, UCHL3, PSMD8, PSMB5, PSMB6]	http://model.geneontology.org/R-HSA-8951664
5	KEAP1-NFE2L2 pathway - Reactome	0.000126	13	57	[PSMD13, PSMA5, PSMD11, PSMA7, PSME2, PSMD4, PSMB1, PSMB7, CSNK2B, PSMD8, PSMB5, PSMB6, PRDX2]	http://model.geneontology.org/R-HSA-9755511
6	SCF(Skp2)-mediated degradation of p27/p21 - Reactome	0.000136	12	50	[PSMD13, PSMA5, PSMD11, PSMA7, PSME2, PSMD4, PSMB1, PSMB7, PSMD8, PSMB5, PSMB6, CKS1B]	http://model.geneontology.org/R-HSA-187577
7	The role of GTSE1 in G2/M progression after G2 checkpoint - Reactome	0.000136	12	50	[PSMD13, PSMA5, PSMD11, PSMA7, PSME2, PSMD4, PSMB1, CDC25B, PSMB7, PSMD8, PSMB5, PSMB6]	http://model.geneontology.org/R-HSA-8852276
8	SCF-beta-TrCP mediated degradation of Emi1 - Reactome	0.000167	12	51	[PSMD13, PSMA5, PSMD11, PSMA7, PSME2, PSMD4, PSMB1, PSMB7, CDK1, PSMD8, PSMB5, PSMB6]	http://model.geneontology.org/R-HSA-174113
9	GSK3B and BTRC:CUL1-mediated-degradation of NFE2L2 - Reactome	0.000175	11	44	[PSMD13, PSMA5, PSMD11, PSMA7, PSME2, PSMD4, PSMB1, PSMB7, PSMD8, PSMB5, PSMB6]	http://model.geneontology.org/R-HSA-9762114
10	Degradation of beta-catenin by the destruction complex - Reactome	0.000183	13	59	[PSMD13, PSMA5, PSMD11, PPP2R5B, PSMA7, PSME2, PSMD4, PSMB1, PSMB7, PPP2R1B, PSMD8, PSMB5, PSMB6]	http://model.geneontology.org/R-HSA-195253
11	Cross-presentation of soluble exogenous antigens (endosomes) - Reactome	0.000217	11	45	[PSMD13, PSMA5, PSMD11, PSMA7, PSME2, PSMD4, PSMB1, PSMB7, PSMD8, PSMB5, PSMB6]	http://model.geneontology.org/R-HSA-1236978
12	AUF1 (hnRNP D0) binds and destabilizes mRNA - Reactome	0.000217	11	45	[PSMD13, PSMA5, PSMD11, PSMA7, PSME2, PSMD4, PSMB1, PSMB7, PSMD8, PSMB5, PSMB6]	http://model.geneontology.org/R-HSA-450408
13	APC/C:Cdc20 mediated degradation of mitotic proteins - Reactome	0.000217	11	45	[PSMD13, PSMA5, PSMD11, PSMA7, PSME2, PSMD4, PSMB1, PSMB7, PSMD8, PSMB5, PSMB6]	http://model.geneontology.org/R-HSA-176409
14	Regulation of activated PAK-2p34 by proteasome mediated degradation - Reactome	0.000268	11	46	[PSMD13, PSMA5, PSMD11, PSMA7, PSME2, PSMD4, PSMB1, PSMB7, PSMD8, PSMB5, PSMB6]	http://model.geneontology.org/R-HSA-211733
15	Ubiquitin-dependent degradation of Cyclin D - Reactome	0.000268	11	46	[PSMD13, PSMA5, PSMD11, PSMA7, PSME2, PSMD4, PSMB1, PSMB7, PSMD8, PSMB5, PSMB6]	http://model.geneontology.org/R-HSA-75815
16	Regulation of RUNX3 expression and activity - Reactome	0.000268	11	46	[PSMD13, PSMA5, PSMD11, PSMA7, PSME2, PSMD4, PSMB1, PSMB7, PSMD8, PSMB5, PSMB6]	http://model.geneontology.org/R-HSA-8941858
17	Antigen Presentation: Folding, assembly and peptide loading of class I MHC - Reactome	0.000297	12	54	[PSMD13, PSMA5, PSMD11, PSMA7, PSME2, PSMD4, PSMB1, PSMB7, PSMD8, PSMB5, PSMB6, ERAP2]	http://model.geneontology.org/R-HSA-983170
18	Degradation of DVL - Reactome	0.000328	11	47	[PSMD13, PSMA5, PSMD11, PSMA7, PSME2, PSMD4, PSMB1, PSMB7, PSMD8, PSMB5, PSMB6]	http://model.geneontology.org/R-HSA-4641258
19	Autodegradation of the E3 ubiquitin ligase COP1 - Reactome	0.000328	11	47	[PSMD13, PSMA5, PSMD11, PSMA7, PSME2, PSMD4, PSMB1, PSMB7, PSMD8, PSMB5, PSMB6]	http://model.geneontology.org/R-HSA-349425
20	Asymmetric localization of PCP proteins - Reactome	0.000328	11	47	[PSMD13, PSMA5, PSMD11, PSMA7, PSME2, PSMD4, PSMB1, PSMB7, PSMD8, PSMB5, PSMB6]	http://model.geneontology.org/R-HSA-4608870
21	TNFR2 non-canonical NF-kB pathway - Reactome	0.000328	11	47	[PSMD13, PSMA5, PSMD11, PSMA7, PSME2, PSMD4, PSMB1, PSMB7, PSMD8, PSMB5, PSMB6]	http://model.geneontology.org/R-HSA-5668541
22	Synthesis of PE - Reactome	0.000338	9	33	[PTDSS2, LPIN1, PCYT2, PNPLA2, PISD, AGPAT2, CHKB, AGPAT4, PHOSPHO1]	http://model.geneontology.org/R-HSA-1483213
23	Heme biosynthesis - Reactome	0.000348	6	15	[ALAS2, UROS, COX10, HMBS, CPOX, PPOX]	http://model.geneontology.org/R-HSA-189451
24	G2/M Checkpoints - Reactome	0.000399	11	48	[PSMD13, PSMA5, PSMD11, PSMA7, PSME2, PSMD4, PSMB1, PSMB7, PSMD8, PSMB5, PSMB6]	http://model.geneontology.org/R-HSA-69481
25	Degradation of AXIN - Reactome	0.000483	11	49	[PSMD13, PSMA5, PSMD11, PSMA7, PSME2, PSMD4, PSMB1, PSMB7, PSMD8, PSMB5, PSMB6]	http://model.geneontology.org/R-HSA-4641257
26	Oxygen-dependent proline hydroxylation of Hypoxia-inducible Factor Alpha - Reactome	0.000483	11	49	[PSMD13, PSMA5, PSMD11, PSMA7, PSME2, PSMD4, PSMB1, PSMB7, PSMD8, PSMB5, PSMB6]	http://model.geneontology.org/R-HSA-1234176
27	RUNX1 regulates transcription of genes involved in differentiation of HSCs - Reactome	0.000483	11	49	[PSMD13, PSMA5, PSMD11, PSMA7, PSME2, PSMD4, PSMB1, PSMB7, PSMD8, PSMB5, PSMB6]	http://model.geneontology.org/R-HSA-8939236
28	Ubiquitin Mediated Degradation of Phosphorylated Cdc25A - Reactome	0.000483	11	49	[PSMD13, PSMA5, PSMD11, PSMA7, PSME2, PSMD4, PSMB1, PSMB7, PSMD8, PSMB5, PSMB6]	http://model.geneontology.org/R-HSA-69601
29	Cyclin A:Cdk2-associated events at S phase entry - Reactome	0.000505	12	57	[PSMD13, PSMA5, PSMD11, PSMA7, PSME2, PSMD4, PSMB1, CDC25B, PSMB7, PSMD8, PSMB5, PSMB6]	http://model.geneontology.org/R-HSA-69656
30	Respiratory electron transport - Reactome	0.000546	16	91	[NDUFS8, ACADVL, COX5B, NDUFS5, NDUFA8, NDUFB7, ETFB, NDUFB8, NDUFV2, COX5A, COX7C, NDUFV1, NDUFA11, NDUFA13, ACADS, UQCRC1]	http://model.geneontology.org/R-HSA-611105
31	FBXL7 down-regulates AURKA during mitotic entry and in early mitosis - Reactome	0.000580	11	50	[PSMD13, PSMA5, PSMD11, PSMA7, PSME2, PSMD4, PSMB1, PSMB7, PSMD8, PSMB5, PSMB6]	http://model.geneontology.org/R-HSA-8854050
32	Hedgehog 'on' state - Reactome	0.000580	11	50	[PSMD13, PSMA5, PSMD11, PSMA7, PSME2, PSMD4, PSMB1, PSMB7, PSMD8, PSMB5, PSMB6]	http://model.geneontology.org/R-HSA-5632684
33	NIK-->noncanonical NF-kB signaling - Reactome	0.000694	11	51	[PSMD13, PSMA5, PSMD11, PSMA7, PSME2, PSMD4, PSMB1, PSMB7, PSMD8, PSMB5, PSMB6]	http://model.geneontology.org/R-HSA-5676590
34	Negative regulation of NOTCH4 signaling - Reactome	0.000694	11	51	[PSMD13, PSMA5, PSMD11, PSMA7, PSME2, PSMD4, PSMB1, PSMB7, PSMD8, PSMB5, PSMB6]	http://model.geneontology.org/R-HSA-9604323
35	Regulation of expression of SLITs and ROBOs - Reactome	0.000694	11	51	[PSMD13, PSMA5, PSMD11, PSMA7, PSME2, PSMD4, PSMB1, PSMB7, PSMD8, PSMB5, PSMB6]	http://model.geneontology.org/R-HSA-9010553
36	Dectin-1 mediated noncanonical NF-kB signaling - Reactome	0.000694	11	51	[PSMD13, PSMA5, PSMD11, PSMA7, PSME2, PSMD4, PSMB1, PSMB7, PSMD8, PSMB5, PSMB6]	http://model.geneontology.org/R-HSA-5607761
37	Cyclin E associated events during G1/S transition - Reactome	0.000703	12	59	[PSMD13, PSMA5, PSMD11, CDK4, PSMA7, PSME2, PSMD4, PSMB1, PSMB7, PSMD8, PSMB5, PSMB6]	http://model.geneontology.org/R-HSA-69202
38	Orc1 removal from chromatin - Reactome	0.000825	11	52	[PSMD13, PSMA5, PSMD11, PSMA7, PSME2, PSMD4, PSMB1, PSMB7, PSMD8, PSMB5, PSMB6]	http://model.geneontology.org/R-HSA-68949
39	Degradation of GLI1 by the proteasome - Reactome	0.000977	11	53	[PSMD13, PSMA5, PSMD11, PSMA7, PSME2, PSMD4, PSMB1, PSMB7, PSMD8, PSMB5, PSMB6]	http://model.geneontology.org/R-HSA-5610780
40	GLI3 is processed to GLI3R by the proteasome - Reactome	0.001151	11	54	[PSMD13, PSMA5, PSMD11, PSMA7, PSME2, PSMD4, PSMB1, PSMB7, PSMD8, PSMB5, PSMB6]	http://model.geneontology.org/R-HSA-5610785
41	Regulation of RUNX2 expression and activity - Reactome	0.001151	11	54	[PSMD13, PSMA5, PSMD11, PSMA7, PSME2, PSMD4, PSMB1, PSMB7, PSMD8, PSMB5, PSMB6]	http://model.geneontology.org/R-HSA-8939902
42	Degradation of GLI2 by the proteasome - Reactome	0.001151	11	54	[PSMD13, PSMA5, PSMD11, PSMA7, PSME2, PSMD4, PSMB1, PSMB7, PSMD8, PSMB5, PSMB6]	http://model.geneontology.org/R-HSA-5610783
43	Hedgehog 'off' state - Reactome	0.001349	11	55	[PSMD13, PSMA5, PSMD11, PSMA7, PSME2, PSMD4, PSMB1, PSMB7, PSMD8, PSMB5, PSMB6]	http://model.geneontology.org/R-HSA-5610787
44	Regulation of PTEN stability and activity - Reactome	0.001495	12	64	[PSMD13, PSMA5, PSMD11, PSMA7, PSME2, PSMD4, PSMB1, PSMB7, CSNK2B, PSMD8, PSMB5, PSMB6]	http://model.geneontology.org/R-HSA-8948751
45	CLEC7A (Dectin-1) signaling - Reactome	0.001575	11	56	[PSMD13, PSMA5, PSMD11, PSMA7, PSME2, PSMD4, PSMB1, PSMB7, PSMD8, PSMB5, PSMB6]	http://model.geneontology.org/R-HSA-5607764
46	FCERI mediated NF-kB activation - Reactome	0.001575	11	56	[PSMD13, PSMA5, PSMD11, PSMA7, PSME2, PSMD4, PSMB1, PSMB7, PSMD8, PSMB5, PSMB6]	http://model.geneontology.org/R-HSA-2871837
47	The proton buffering model - Reactome	0.001795	13	74	[NDUFS8, COX5B, NDUFS5, NDUFA8, NDUFB7, NDUFB8, NDUFV2, COX5A, COX7C, NDUFV1, NDUFA11, NDUFA13, UQCRC1]	http://model.geneontology.org/R-HSA-167827
48	The fatty acid cycling model - Reactome	0.001795	13	74	[NDUFS8, COX5B, NDUFS5, NDUFA8, NDUFB7, NDUFB8, NDUFV2, COX5A, COX7C, NDUFV1, NDUFA11, NDUFA13, UQCRC1]	http://model.geneontology.org/R-HSA-167826

Repeat with weighted step enrichment:

In [5]:
```
results_weighted = enrich.enrich_wrapper(file_name,'Gene Symbol',method='ncHGT',FDR = 0.05,fpath = file_path)
results_weighted
```

```
100%|██████████████████████████████| 482/482 [01:19<00:00,  6.09it/s]
Analysis run on 423 entities from 365 out of 1172 input genes
```

Out[5]:

	title	pval (uncorrected)	# entities in list	#entities in model	shared entities in gocam	url
0	Collagen biosynthesis and modifying enzymes - Reactome	4.680930e-07	10	12	[PPIB, P3H1, PLOD3, P4HB, set:Prolyl 3-hydroxylases, set:COLGALT1,COLGALT2, set:4-Hyp collagen propeptides, set:Procollagen N-proteinases, set:Procollagen C-proteinases, set:Lysyl hydroxylases]	http://model.geneontology.org/R-HSA-1650814
1	Hedgehog ligand biogenesis - Reactome	4.690733e-06	13	50	[PSMD13, PSMA5, PSMD11, PSMA7, PSME2, PSMD4, PSMB1, PSMB7, PSMD8, PSMB5, P4HB, PSMB6, SYVN1]	http://model.geneontology.org/R-HSA-5358346
2	ER-Phagosome pathway - Reactome	5.123754e-06	13	51	[PSMD13, PSMA5, PSMD11, SEC61B, PSMA7, PSME2, PSMD4, PSMB1, PSMB7, PSMD8, PSMB5, PSMB6, set:SEC61 alpha]	http://model.geneontology.org/R-HSA-1236974
3	Regulation of APC/C activators between G1/S and early anaphase - Reactome	7.226495e-06	13	52	[PSMD13, PSMA5, PSMD11, PSMA7, PSME2, PSMD4, PSMB1, PSMB7, CDK1, PSMD8, PSMB5, PSMB6, set:CDC25]	http://model.geneontology.org/R-HSA-176408
4	Neddylation - Reactome	9.256658e-06	14	62	[UBE2M, PSMD13, PSMA5, PSMD11, PSMA7, PSME2, PSMD4, PSMB1, CUL9, PSMB7, PSMD8, PSMB5, PSMB6, set:UCHL3,SENP8]	http://model.geneontology.org/R-HSA-8951664
5	KEAP1-NFE2L2 pathway - Reactome	1.540320e-05	13	55	[PSMD13, PSMA5, PSMD11, PSMA7, PSME2, PSMD4, PSMB1, PSMB7, CSNK2B, PSMD8, PSMB5, PSMB6, set:PRDX1,2,5]	http://model.geneontology.org/R-HSA-9755511
6	The role of GTSE1 in G2/M progression after G2 checkpoint - Reactome	1.767235e-05	12	50	[PSMD13, PSMA5, PSMD11, PSMA7, PSME2, PSMD4, PSMB1, CDC25B, PSMB7, PSMD8, PSMB5, PSMB6]	http://model.geneontology.org/R-HSA-8852276
7	Conversion from APC/C:Cdc20 to APC/C:Cdh1 in late anaphase - Reactome	1.767235e-05	12	49	[PSMD13, PSMA5, PSMD11, PSMA7, PSME2, PSMD4, PSMB1, PSMB7, CDK1, PSMD8, PSMB5, PSMB6]	http://model.geneontology.org/R-HSA-176407
8	Degradation of beta-catenin by the destruction complex - Reactome	2.044777e-05	13	54	[PSMD13, PSMA5, PSMD11, PSMA7, PSME2, PSMD4, PSMB1, PSMB7, PSMD8, PSMB5, PSMB6, set:PP2A-subunit A, set:PP2A regulatory subunit B56]	http://model.geneontology.org/R-HSA-195253
9	SCF(Skp2)-mediated degradation of p27/p21 - Reactome	2.069350e-05	12	49	[PSMD13, PSMA5, PSMD11, PSMA7, PSME2, PSMD4, PSMB1, PSMB7, PSMD8, PSMB5, PSMB6, CKS1B]	http://model.geneontology.org/R-HSA-187577
10	Antigen processing: Ubiquitination & Proteasome degradation - Reactome	2.225907e-05	14	50	[PSMD13, PSMA5, PSMD11, PSMA7, PSME2, PSMD4, PSMB1, PSMB7, PSMD8, PSMB5, PSMB6, set:Activating enzymes E1, set:E2 Ubiquitin-conjugating enzyme, set:E3 ligases in proteasomal degradation]	http://model.geneontology.org/R-HSA-983168
11	SCF-beta-TrCP mediated degradation of Emi1 - Reactome	2.651483e-05	12	51	[PSMD13, PSMA5, PSMD11, PSMA7, PSME2, PSMD4, PSMB1, PSMB7, CDK1, PSMD8, PSMB5, PSMB6]	http://model.geneontology.org/R-HSA-174113
12	Respiratory electron transport - Reactome	3.130019e-05	16	91	[NDUFS8, ACADVL, COX5B, NDUFS5, NDUFA8, NDUFB7, ETFB, NDUFB8, NDUFV2, COX5A, COX7C, NDUFV1, NDUFA11, NDUFA13, ACADS, UQCRC1]	http://model.geneontology.org/R-HSA-611105
13	Antigen Presentation: Folding, assembly and peptide loading of class I MHC - Reactome	3.672378e-05	12	50	[PSMD13, PSMA5, PSMD11, PSMA7, PSME2, PSMD4, PSMB1, PSMB7, PSMD8, PSMB5, PSMB6, set:ERAP1/2]	http://model.geneontology.org/R-HSA-983170
14	GSK3B and BTRC:CUL1-mediated-degradation of NFE2L2 - Reactome	3.794569e-05	11	44	[PSMD13, PSMA5, PSMD11, PSMA7, PSME2, PSMD4, PSMB1, PSMB7, PSMD8, PSMB5, PSMB6]	http://model.geneontology.org/R-HSA-9762114
15	APC/C:Cdc20 mediated degradation of mitotic proteins - Reactome	4.675146e-05	11	45	[PSMD13, PSMA5, PSMD11, PSMA7, PSME2, PSMD4, PSMB1, PSMB7, PSMD8, PSMB5, PSMB6]	http://model.geneontology.org/R-HSA-176409
16	Cross-presentation of soluble exogenous antigens (endosomes) - Reactome	4.675146e-05	11	45	[PSMD13, PSMA5, PSMD11, PSMA7, PSME2, PSMD4, PSMB1, PSMB7, PSMD8, PSMB5, PSMB6]	http://model.geneontology.org/R-HSA-1236978
17	AUF1 (hnRNP D0) binds and destabilizes mRNA - Reactome	4.675146e-05	11	45	[PSMD13, PSMA5, PSMD11, PSMA7, PSME2, PSMD4, PSMB1, PSMB7, PSMD8, PSMB5, PSMB6]	http://model.geneontology.org/R-HSA-450408
18	Regulation of RUNX3 expression and activity - Reactome	5.465938e-05	11	45	[PSMD13, PSMA5, PSMD11, PSMA7, PSME2, PSMD4, PSMB1, PSMB7, PSMD8, PSMB5, PSMB6]	http://model.geneontology.org/R-HSA-8941858
19	Regulation of activated PAK-2p34 by proteasome mediated degradation - Reactome	5.724543e-05	11	46	[PSMD13, PSMA5, PSMD11, PSMA7, PSME2, PSMD4, PSMB1, PSMB7, PSMD8, PSMB5, PSMB6]	http://model.geneontology.org/R-HSA-211733
20	Ubiquitin-dependent degradation of Cyclin D - Reactome	5.724543e-05	11	46	[PSMD13, PSMA5, PSMD11, PSMA7, PSME2, PSMD4, PSMB1, PSMB7, PSMD8, PSMB5, PSMB6]	http://model.geneontology.org/R-HSA-75815
21	Cyclin A:Cdk2-associated events at S phase entry - Reactome	6.418233e-05	12	52	[PSMD13, PSMA5, PSMD11, PSMA7, PSME2, PSMD4, PSMB1, PSMB7, PSMD8, PSMB5, PSMB6, set:Cdc25 A/B]	http://model.geneontology.org/R-HSA-69656
22	Asymmetric localization of PCP proteins - Reactome	6.666665e-05	11	46	[PSMD13, PSMA5, PSMD11, PSMA7, PSME2, PSMD4, PSMB1, PSMB7, PSMD8, PSMB5, PSMB6]	http://model.geneontology.org/R-HSA-4608870
23	Degradation of DVL - Reactome	6.968340e-05	11	47	[PSMD13, PSMA5, PSMD11, PSMA7, PSME2, PSMD4, PSMB1, PSMB7, PSMD8, PSMB5, PSMB6]	http://model.geneontology.org/R-HSA-4641258
24	Autodegradation of the E3 ubiquitin ligase COP1 - Reactome	6.968340e-05	11	47	[PSMD13, PSMA5, PSMD11, PSMA7, PSME2, PSMD4, PSMB1, PSMB7, PSMD8, PSMB5, PSMB6]	http://model.geneontology.org/R-HSA-349425
25	G2/M Checkpoints - Reactome	6.968340e-05	11	48	[PSMD13, PSMA5, PSMD11, PSMA7, PSME2, PSMD4, PSMB1, PSMB7, PSMD8, PSMB5, PSMB6]	http://model.geneontology.org/R-HSA-69481
26	Cyclin E associated events during G1/S transition - Reactome	7.453099e-05	12	51	[PSMD13, PSMA5, PSMD11, PSMA7, PSME2, PSMD4, PSMB1, PSMB7, PSMD8, PSMB5, PSMB6, set:p-T172-CDK4,p-T177-CDK6:CCND:CDKN1A,CDKN1B,(CDKN1C); p-T172-CDK4,p-T177-CDK6:CCND; (p-T172-CDK4,p-T177-CDK6:CCND:p-Y88-CDKN1B)]	http://model.geneontology.org/R-HSA-69202
27	TNFR2 non-canonical NF-kB pathway - Reactome	9.357132e-05	11	47	[PSMD13, PSMA5, PSMD11, PSMA7, PSME2, PSMD4, PSMB1, PSMB7, PSMD8, PSMB5, PSMB6]	http://model.geneontology.org/R-HSA-5668541
28	Degradation of AXIN - Reactome	9.750828e-05	11	48	[PSMD13, PSMA5, PSMD11, PSMA7, PSME2, PSMD4, PSMB1, PSMB7, PSMD8, PSMB5, PSMB6]	http://model.geneontology.org/R-HSA-4641257
29	Ubiquitin Mediated Degradation of Phosphorylated Cdc25A - Reactome	1.015570e-04	11	49	[PSMD13, PSMA5, PSMD11, PSMA7, PSME2, PSMD4, PSMB1, PSMB7, PSMD8, PSMB5, PSMB6]	http://model.geneontology.org/R-HSA-69601
30	RUNX1 regulates transcription of genes involved in differentiation of HSCs - Reactome	1.015570e-04	11	49	[PSMD13, PSMA5, PSMD11, PSMA7, PSME2, PSMD4, PSMB1, PSMB7, PSMD8, PSMB5, PSMB6]	http://model.geneontology.org/R-HSA-8939236
31	Oxygen-dependent proline hydroxylation of Hypoxia-inducible Factor Alpha - Reactome	1.124556e-04	11	48	[PSMD13, PSMA5, PSMD11, PSMA7, PSME2, PSMD4, PSMB1, PSMB7, PSMD8, PSMB5, PSMB6]	http://model.geneontology.org/R-HSA-1234176
32	Hedgehog 'on' state - Reactome	1.169920e-04	11	49	[PSMD13, PSMA5, PSMD11, PSMA7, PSME2, PSMD4, PSMB1, PSMB7, PSMD8, PSMB5, PSMB6]	http://model.geneontology.org/R-HSA-5632684
33	Hedgehog 'off' state - Reactome	1.190213e-04	11	46	[PSMD13, PSMA5, PSMD11, PSMA7, PSME2, PSMD4, PSMB1, PSMB7, PSMD8, PSMB5, PSMB6]	http://model.geneontology.org/R-HSA-5610787
34	FBXL7 down-regulates AURKA during mitotic entry and in early mitosis - Reactome	1.216519e-04	11	50	[PSMD13, PSMA5, PSMD11, PSMA7, PSME2, PSMD4, PSMB1, PSMB7, PSMD8, PSMB5, PSMB6]	http://model.geneontology.org/R-HSA-8854050
35	Synthesis of PE - Reactome	1.235147e-04	8	15	[PTDSS2, PCYT2, PISD, PHOSPHO1, set:LPIN, set:CHK/ETNK, set:PNPLA2/3, set:AGPAT]	http://model.geneontology.org/R-HSA-1483213
36	Negative regulation of NOTCH4 signaling - Reactome	1.344659e-04	11	49	[PSMD13, PSMA5, PSMD11, PSMA7, PSME2, PSMD4, PSMB1, PSMB7, PSMD8, PSMB5, PSMB6]	http://model.geneontology.org/R-HSA-9604323
37	Dectin-1 mediated noncanonical NF-kB signaling - Reactome	1.396715e-04	11	50	[PSMD13, PSMA5, PSMD11, PSMA7, PSME2, PSMD4, PSMB1, PSMB7, PSMD8, PSMB5, PSMB6]	http://model.geneontology.org/R-HSA-5607761
38	NIK-->noncanonical NF-kB signaling - Reactome	1.396715e-04	11	50	[PSMD13, PSMA5, PSMD11, PSMA7, PSME2, PSMD4, PSMB1, PSMB7, PSMD8, PSMB5, PSMB6]	http://model.geneontology.org/R-HSA-5676590
39	Regulation of expression of SLITs and ROBOs - Reactome	1.450130e-04	11	51	[PSMD13, PSMA5, PSMD11, PSMA7, PSME2, PSMD4, PSMB1, PSMB7, PSMD8, PSMB5, PSMB6]	http://model.geneontology.org/R-HSA-9010553
40	Orc1 removal from chromatin - Reactome	1.659552e-04	11	51	[PSMD13, PSMA5, PSMD11, PSMA7, PSME2, PSMD4, PSMB1, PSMB7, PSMD8, PSMB5, PSMB6]	http://model.geneontology.org/R-HSA-68949
41	Regulation of PTEN stability and activity - Reactome	1.722838e-04	12	56	[PSMD13, PSMA5, PSMD11, PSMA7, PSME2, PSMD4, PSMB1, PSMB7, CSNK2B, PSMD8, PSMB5, PSMB6]	http://model.geneontology.org/R-HSA-8948751
42	The proton buffering model - Reactome	1.826341e-04	13	70	[NDUFS8, COX5B, NDUFS5, NDUFA8, NDUFB7, NDUFB8, NDUFV2, COX5A, COX7C, NDUFV1, NDUFA11, NDUFA13, UQCRC1]	http://model.geneontology.org/R-HSA-167827
43	The fatty acid cycling model - Reactome	1.826341e-04	13	70	[NDUFS8, COX5B, NDUFS5, NDUFA8, NDUFB7, NDUFB8, NDUFV2, COX5A, COX7C, NDUFV1, NDUFA11, NDUFA13, UQCRC1]	http://model.geneontology.org/R-HSA-167826
44	Degradation of GLI1 by the proteasome - Reactome	1.850326e-04	11	51	[PSMD13, PSMA5, PSMD11, PSMA7, PSME2, PSMD4, PSMB1, PSMB7, PSMD8, PSMB5, PSMB6]	http://model.geneontology.org/R-HSA-5610780
45	Degradation of GLI2 by the proteasome - Reactome	2.183641e-04	11	52	[PSMD13, PSMA5, PSMD11, PSMA7, PSME2, PSMD4, PSMB1, PSMB7, PSMD8, PSMB5, PSMB6]	http://model.geneontology.org/R-HSA-5610783
46	GLI3 is processed to GLI3R by the proteasome - Reactome	2.183641e-04	11	52	[PSMD13, PSMA5, PSMD11, PSMA7, PSME2, PSMD4, PSMB1, PSMB7, PSMD8, PSMB5, PSMB6]	http://model.geneontology.org/R-HSA-5610785
47	Regulation of RUNX2 expression and activity - Reactome	2.390173e-04	11	54	[PSMD13, PSMA5, PSMD11, PSMA7, PSME2, PSMD4, PSMB1, PSMB7, PSMD8, PSMB5, PSMB6]	http://model.geneontology.org/R-HSA-8939902
48	Regulation of RAS by GAPs - Reactome	2.638305e-04	11	48	[PSMD13, PSMA5, PSMD11, PSMA7, PSME2, PSMD4, PSMB1, PSMB7, PSMD8, PSMB5, PSMB6]	http://model.geneontology.org/R-HSA-5658442
49	FCERI mediated NF-kB activation - Reactome	3.068243e-04	11	54	[PSMD13, PSMA5, PSMD11, PSMA7, PSME2, PSMD4, PSMB1, PSMB7, PSMD8, PSMB5, PSMB6]	http://model.geneontology.org/R-HSA-2871837
50	CLEC7A (Dectin-1) signaling - Reactome	3.166147e-04	11	55	[PSMD13, PSMA5, PSMD11, PSMA7, PSME2, PSMD4, PSMB1, PSMB7, PSMD8, PSMB5, PSMB6]	http://model.geneontology.org/R-HSA-5607764
51	Formation of ATP by chemiosmotic coupling - Reactome	3.580515e-04	14	88	[ATP5F1D, NDUFS8, COX5B, NDUFS5, NDUFA8, NDUFB7, NDUFB8, NDUFV2, COX5A, COX7C, NDUFV1, NDUFA11, NDUFA13, UQCRC1]	http://model.geneontology.org/R-HSA-163210
52	Cyclin A/B1/B2 associated events during G2/M transition - Reactome	3.789603e-04	7	17	[LCMT1, CDC25B, CDK1, PPP2R2A, set:CRS kinase, set:PP2A-subunit A, set:CDC25]	http://model.geneontology.org/R-HSA-69273
53	Activation of NF-kappaB in B cells - Reactome	3.828590e-04	11	54	[PSMD13, PSMA5, PSMD11, PSMA7, PSME2, PSMD4, PSMB1, PSMB7, PSMD8, PSMB5, PSMB6]	http://model.geneontology.org/R-HSA-1169091
54	Heme biosynthesis - Reactome	4.785104e-04	6	14	[UROS, COX10, HMBS, CPOX, PPOX, set:ALAS1,2]	http://model.geneontology.org/R-HSA-189451
55	Autodegradation of Cdh1 by Cdh1:APC/C - Reactome	5.072445e-04	11	59	[PSMD13, PSMA5, PSMD11, PSMA7, PSME2, PSMD4, PSMB1, PSMB7, PSMD8, PSMB5, PSMB6]	http://model.geneontology.org/R-HSA-174084
56	MAPK6/MAPK4 signaling - Reactome	5.695231e-04	12	62	[PSMD13, PSMA5, PSMD11, PSMA7, PSME2, PSMD4, PSMB1, PSMB7, CDK1, PSMD8, PSMB5, PSMB6]	http://model.geneontology.org/R-HSA-5687128
57	Assembly of the pre-replicative complex - Reactome	5.833446e-04	11	60	[PSMD13, PSMA5, PSMD11, PSMA7, PSME2, PSMD4, PSMB1, PSMB7, PSMD8, PSMB5, PSMB6]	http://model.geneontology.org/R-HSA-68867
58	ABC-family proteins mediated transport - Reactome	6.243729e-04	11	54	[PSMD13, PSMA5, PSMD11, PSMA7, PSME2, PSMD4, PSMB1, PSMB7, PSMD8, PSMB5, PSMB6]	http://model.geneontology.org/R-HSA-382556
59	APC/C:Cdc20 mediated degradation of Securin - Reactome	7.640748e-04	11	62	[PSMD13, PSMA5, PSMD11, PSMA7, PSME2, PSMD4, PSMB1, PSMB7, PSMD8, PSMB5, PSMB6]	http://model.geneontology.org/R-HSA-174154
60	Cdc20:Phospho-APC/C mediated degradation of Cyclin A - Reactome	7.640748e-04	11	62	[PSMD13, PSMA5, PSMD11, PSMA7, PSME2, PSMD4, PSMB1, PSMB7, PSMD8, PSMB5, PSMB6]	http://model.geneontology.org/R-HSA-174184
61	APC/C:Cdh1 mediated degradation of Cdc20 and other APC/C:Cdh1 targeted proteins in late mitosis/early G1 - Reactome	7.640748e-04	11	62	[PSMD13, PSMA5, PSMD11, PSMA7, PSME2, PSMD4, PSMB1, PSMB7, PSMD8, PSMB5, PSMB6]	http://model.geneontology.org/R-HSA-174178
62	Separation of Sister Chromatids - Reactome	8.704360e-04	11	63	[PSMD13, PSMA5, PSMD11, PSMA7, PSME2, PSMD4, PSMB1, PSMB7, PSMD8, PSMB5, PSMB6]	http://model.geneontology.org/R-HSA-2467813
63	CDK-mediated phosphorylation and removal of Cdc6 - Reactome	1.441100e-03	11	65	[PSMD13, PSMA5, PSMD11, PSMA7, PSME2, PSMD4, PSMB1, PSMB7, PSMD8, PSMB5, PSMB6]	http://model.geneontology.org/R-HSA-69017
64	Interleukin-1 signaling - Reactome	1.727041e-03	11	64	[PSMD13, PSMA5, PSMD11, PSMA7, PSME2, PSMD4, PSMB1, PSMB7, PSMD8, PSMB5, PSMB6]	http://model.geneontology.org/R-HSA-9020702
65	Downstream TCR signaling - Reactome	1.813878e-03	14	74	[PSMD13, PSMA5, PSMD11, PSMA7, PSME2, PSMD4, PSMB1, PSMB7, PSMD8, PSMB5, PSMB6, CD3D, set:PIK3C(1), set:Activator:PI3K]	http://model.geneontology.org/R-HSA-202424

Repeat but with FDR = 0.1 instead of FDR = 0.05:

In [6]:
```
results_set = enrich.enrich_wrapper('platelets_down.csv','Gene Symbol',method='ncHGT',FDR = 0.5,fpath = file_path)
results_set
```

```
100%|██████████████████████████████| 436/436 [02:12<00:00,  3.28it/s]
Analysis run on 309 entities from 267 out of 1088 input genes
```

Out[6]:

	title	pval (uncorrected)	# entities in list	#entities in model	shared entities in gocam	url
0	Role of phospholipids in phagocytosis - Reactome	0.000010	11	22	[set:PLCdegh, set:PLC-beta, set:G-protein gamma subunit, set:PL(C)D4:3xCa2+, set:PLC-beta 1/2/3, set:PLCbz, set:G-protein alpha (q/11/14/15), set:PI3K-regulatory subunit, set:G(q) alpha 11,14,15,Q, set:PLD, set:PLC beta1,2,3]	http://model.geneontology.org/R-HSA-2029485
1	DAG and IP3 signaling - Reactome	0.000019	10	18	[set:PLCdegh, set:PLC-beta, set:G-protein gamma subunit, set:PL(C)D4:3xCa2+, set:PLC-beta 1/2/3, set:PLCbz, set:G-protein alpha (q/11/14/15), set:PI3K-regulatory subunit, set:G(q) alpha 11,14,15,Q, set:PLC beta1,2,3]	http://model.geneontology.org/R-HSA-1489509
2	VEGFR2 mediated cell proliferation - Reactome	0.000046	11	27	[KDR, set:PLCdegh, set:PLC-beta, set:G-protein gamma subunit, set:PL(C)D4:3xCa2+, set:PLC-beta 1/2/3, set:PLCbz, set:G-protein alpha (q/11/14/15), set:PI3K-regulatory subunit, set:G(q) alpha 11,14,15,Q, set:PLC beta1,2,3]	http://model.geneontology.org/R-HSA-5218921
3	PLC beta mediated events - Reactome	0.000050	9	15	[set:PLCdegh, set:PLC-beta, set:G-protein gamma subunit, set:PL(C)D4:3xCa2+, set:PLC-beta 1/2/3, set:PLCbz, set:G-protein alpha (q/11/14/15), set:G(q) alpha 11,14,15,Q, set:PLC beta1,2,3]	http://model.geneontology.org/R-HSA-112043
4	Antigen activates B Cell Receptor (BCR) leading to generation of second messengers - Reactome	0.000071	11	25	[PIK3R1, set:PLCdegh, set:PLC-beta, set:G-protein gamma subunit, set:PL(C)D4:3xCa2+, set:PLC-beta 1/2/3, set:PLCbz, set:G-protein alpha (q/11/14/15), set:LYN, p-SYK, set:G(q) alpha 11,14,15,Q, set:PLC beta1,2,3]	http://model.geneontology.org/R-HSA-983695
5	CLEC7A (Dectin-1) induces NFAT activation - Reactome	0.000403	9	22	[set:PLCdegh, set:PLC-beta, set:G-protein gamma subunit, set:PL(C)D4:3xCa2+, set:PLC-beta 1/2/3, set:PLCbz, set:G-protein alpha (q/11/14/15), set:G(q) alpha 11,14,15,Q, set:PLC beta1,2,3]	http://model.geneontology.org/R-HSA-5607763
6	Regulation of insulin secretion - Reactome	0.000444	9	22	[set:PLCdegh, set:PLC-beta, set:G-protein gamma subunit, set:PL(C)D4:3xCa2+, set:PLC-beta 1/2/3, set:PLCbz, set:G-protein alpha (q/11/14/15), set:G(q) alpha 11,14,15,Q, set:PLC beta1,2,3]	http://model.geneontology.org/R-HSA-422356
7	Synthesis of IP2, IP, and Ins in the cytosol - Reactome	0.000664	7	17	[set:PLCdegh, set:INPP5(3)/ITPK1, set:PL(C)D4:3xCa2+, set:PLCbz, set:IMPA1/2, set:Inositol polyphosphate 5-phosphatase, set:INPP5(4)]	http://model.geneontology.org/R-HSA-1855183
8	G alpha (12/13) signalling events - Reactome	0.000792	8	13	[GNA13, set:Ligand:GPCR complexes that activate Gi, set:ARHGEF11,ARHGEF12, set:G-protein G12/G13 (inactive), set:G-protein gamma subunit, set:GEFs, set:G-protein alpha (12/13):GTP, set:G alpha (i)]	http://model.geneontology.org/R-HSA-416482
9	Ca2+ pathway - Reactome	0.001121	9	27	[set:PLCdegh, set:PLC-beta, set:G-protein gamma subunit, set:PL(C)D4:3xCa2+, set:PLC-beta 1/2/3, set:PLCbz, set:G-protein alpha (q/11/14/15), set:G(q) alpha 11,14,15,Q, set:PLC beta1,2,3]	http://model.geneontology.org/R-HSA-4086398
10	Synthesis of very long-chain fatty acyl-CoAs - Reactome	0.001138	6	12	[ELOVL7, set:ELOVL3,6,7, set:ACSBG1,2, set:HSD17B3,12, set:PTPLs, set:ACSL3,4]	http://model.geneontology.org/R-HSA-75876
11	FCERI mediated Ca+2 mobilization - Reactome	0.001206	9	26	[set:PLCdegh, set:PLC-beta, set:G-protein gamma subunit, set:PL(C)D4:3xCa2+, set:PLC-beta 1/2/3, set:PLCbz, set:G-protein alpha (q/11/14/15), set:G(q) alpha 11,14,15,Q, set:PLC beta1,2,3]	http://model.geneontology.org/R-HSA-2871809
12	VEGFR2 mediated vascular permeability - Reactome	0.001440	8	20	[PIK3R1, PIK3CG, set:PAK1,2,3 dimer, set:p-S,T-PAK1,2,3, set:G-protein gamma subunit, set:PI3K-regulatory subunit, set:Activator:PI3K, set:PI3K alpha, beta, gamma]	http://model.geneontology.org/R-HSA-5218920
