# Supplementary material for: Enrichment on steps, not genes, improves inference of differentially expressed pathways
Source: PLoS Comput Biol. 2024 Mar 25;20(3):e1011968. doi: 10.1371/journal.pcbi.1011968 (PMC10994554; doi:10.1371/journal.pcbi.1011968)
Supplement: S2 File — Enrichment analysis results with FDR = 0.1 used to produce Figs 5 and 7. (PDF) [file pcbi.1011968.s005.pdf]

```
In [1]: import pandas as pd
        import numpy as np
        import sys
        sys.path.append('../dev')

        import utils
        import enrich_ as enrich

        pd.options.display.max_colwidth = 300
        pd.set_option('display.max_rows', None)

        !date
```

```
Tue Mar 12 17:01:45 PDT 2024
```

Below are all the enrichment results at FDR 0.1 used to produce Figures 5 and 7.

p97 Inhibitors

Wang et al., 2022

https://www.sciencedirect.com/science/article/pii/S2451945621004827

shRNA knockdown of p97 in a colon cancer cell line.

```
In [2]: p97_step = enrich.enrich_wrapper('P97.csv','uniprot',FDR = 0.1, method='set', fpath = '../test_data/processed',table_number = 1)
        p97_step
```

```
100%|██████████████████████████████| 480/480 [00:00<00:00, 16996.47it/s]
Analysis run on 338 entities from 270 out of 766 input genes
```

Out[2]: **Table SAA: P97 combined up/downregulated proteins, unweighted set method, FDR = 0.1.**

	title	pval (uncorrected)	# entities in list	#entities in model	shared entities in gocam	url
0	PI5P, PP2A and IER3 Regulate PI3K/AKT Signaling - Reactome	0.000002	8	13	['set:PP2A-subunit A', 'set:PIP5K1A/B', 'set:PI4K2A/2B', 'set:SYNJ/MTM(1)', 'set:Activated SRC,LCK,EGFR,INSR', 'set:Activator:PI3K', 'set:PP2A-catalytic subunit C', 'set:PIP5K1A-C']	http://model.geneontology.org/R-HSA-6811558
1	Unwinding of DNA - Reactome	0.000002	6	7	['MCM3', 'MCM6', 'MCM4', 'MCM2', 'MCM7', 'MCM5']	http://model.geneontology.org/R-HSA-176974

```
In [3]: p97_standard = enrich.enrich_wrapper('P97.csv','uniprot', FDR = 0.1, method='standard', fpath = '../test_data/processed', table_number = 2)
        p97_standard
```

```
100%|██████████████████████████████| 480/480 [00:00<00:00, 17953.95it/s]
```

Out[3]: **Table SAB: P97 combined up/downregulated proteins, standard method, FDR = 0.1.**

	title	pval (uncorrected)	# entities in list	#entities in model	shared entities in gocam	url
0	Unwinding of DNA - Reactome	0.000000	6	7	['MCM3', 'MCM6', 'MCM4', 'MCM2', 'MCM7', 'MCM5']	http://model.geneontology.org/R-HSA-176974
1	SCF(Skp2)-mediated degradation of p27/p21 - Reactome	0.000001	13	50	['PSMD2', 'PSMC4', 'PSMC5', 'PSMC1', 'PSMD11', 'PSMC2', 'PSMD13', 'PSMD14', 'CDK2', 'CDKN1A', 'PSMF1', 'CDKN1B', 'CKS1B']	http://model.geneontology.org/R-HSA-187577
2	Autodegradation of Cdh1 by Cdh1:APC/C - Reactome	0.000005	13	59	['PSMD2', 'PSMC4', 'PSMC5', 'PSMC1', 'PSMD11', 'PSMC2', 'PSMD13', 'PSMD14', 'PSMF1', 'UBE2S', 'UBE2C', 'UBE2E1', 'ANAPC2']	http://model.geneontology.org/R-HSA-174084
3	CDK-mediated phosphorylation and removal of Cdc6 - Reactome	0.000005	14	68	['PSMD2', 'PSMC4', 'PSMC5', 'PSMC1', 'PSMD11', 'PSMC2', 'PSMD13', 'PSMD14', 'CDK2', 'PSMF1', 'UBE2S', 'UBE2C', 'UBE2E1', 'ANAPC2']	http://model.geneontology.org/R-HSA-69017
4	Assembly of the pre-replicative complex - Reactome	0.000006	13	60	['PSMD2', 'PSMC4', 'PSMC5', 'PSMC1', 'PSMD11', 'PSMC2', 'PSMD13', 'PSMD14', 'PSMF1', 'UBE2S', 'UBE2C', 'UBE2E1', 'ANAPC2']	http://model.geneontology.org/R-HSA-68867
5	APC/C:Cdh1 mediated degradation of Cdc20 and other APC/C:Cdh1 targeted proteins in late mitosis/early G1 - Reactome	0.000009	13	62	['PSMD2', 'PSMC4', 'PSMC5', 'PSMC1', 'PSMD11', 'PSMC2', 'PSMD13', 'PSMD14', 'PSMF1', 'UBE2S', 'UBE2C', 'UBE2E1', 'ANAPC2']	http://model.geneontology.org/R-HSA-174178
6	APC/C:Cdc20 mediated degradation of Securin - Reactome	0.000009	13	62	['PSMD2', 'PSMC4', 'PSMC5', 'PSMC1', 'PSMD11', 'PSMC2', 'PSMD13', 'PSMD14', 'PSMF1', 'UBE2S', 'UBE2C', 'UBE2E1', 'ANAPC2']	http://model.geneontology.org/R-HSA-174154
7	Cdc20:Phospho-APC/C mediated degradation of Cyclin A - Reactome	0.000009	13	62	['PSMD2', 'PSMC4', 'PSMC5', 'PSMC1', 'PSMD11', 'PSMC2', 'PSMD13', 'PSMD14', 'PSMF1', 'UBE2S', 'UBE2C', 'UBE2E1', 'ANAPC2']	http://model.geneontology.org/R-HSA-174184
8	Separation of Sister Chromatids - Reactome	0.000010	13	63	['PSMD2', 'PSMC4', 'PSMC5', 'PSMC1', 'PSMD11', 'PSMC2', 'PSMD13', 'PSMD14', 'PSMF1', 'UBE2S', 'UBE2C', 'UBE2E1', 'ANAPC2']	http://model.geneontology.org/R-HSA-2467813
9	Antigen processing: Ubiquitination & Proteasome degradation - Reactome	0.000017	33	305	['HUWE1', 'HECTD1', 'PSMD2', 'TRIP12', 'PSMC4', 'PSMC5', 'PSMC1', 'PSMD11', 'PSMC2', 'PSMD13', 'UBE3A', 'PSMD14', 'FBXO22', 'UBE2J1', 'PSMF1', 'UBE2S', 'UBE2D2', 'UBE2E2', 'UBE2L6', 'UBE2C', 'RNF7', 'UBE2H', 'UBE2A', 'UBE2E1', 'UBE2R2', 'KEAP1', 'ANAPC2', 'UBE2J2', 'CDC20', 'UBE2W', 'UBAC1', 'ASB6', 'KLHL25']	http://model.geneontology.org/R-HSA-983168
10	Cyclin A:Cdk2-associated events at S phase entry - Reactome	0.000018	12	57	['PSMD2', 'PSMC4', 'PSMC5', 'PSMC1', 'PSMD11', 'PSMC2', 'PSMD13', 'PSMD14', 'CDK2', 'CDKN1A', 'PSMF1', 'CDKN1B']	http://model.geneontology.org/R-HSA-69656
11	Conversion from APC/C:Cdc20 to APC/C:Cdh1 in late anaphase - Reactome	0.000022	11	49	['PSMD2', 'PSMC4', 'PSMC5', 'PSMC1', 'PSMD11', 'PSMC2', 'PSMD13', 'CDK1', 'PSMD14', 'CDK2', 'PSMF1']	http://model.geneontology.org/R-HSA-176407
12	The role of GTSE1 in G2/M progression after G2 checkpoint - Reactome	0.000027	11	50	['PSMD2', 'PSMC4', 'PSMC5', 'PSMC1', 'PSMD11', 'PSMC2', 'PSMD13', 'PSMD14', 'PSMF1', 'PLK1', 'TP53']	http://model.geneontology.org/R-HSA-8852276
13	SCF-beta-TrCP mediated degradation of Emi1 - Reactome	0.000032	11	51	['PSMD2', 'PSMC4', 'PSMC5', 'PSMC1', 'PSMD11', 'PSMC2', 'PSMD13', 'CDK1', 'PSMD14', 'PSMF1', 'PLK1']	http://model.geneontology.org/R-HSA-174113
14	ER-Phagosome pathway - Reactome	0.000039	11	52	['PSMD2', 'PSMC4', 'PSMC5', 'PSMC1', 'PSMD11', 'PSMC2', 'PSMD13', 'PSMD14', 'PSMF1', 'SEC61A1', 'SEC61G']	http://model.geneontology.org/R-HSA-1236974
15	Regulation of APC/C activators between G1/S and early anaphase - Reactome	0.000057	11	54	['PSMD2', 'PSMC4', 'PSMC5', 'PSMC1', 'PSMD11', 'PSMC2', 'PSMD13', 'CDK1', 'PSMD14', 'CDK2', 'PSMF1']	http://model.geneontology.org/R-HSA-176408
16	G2/M Checkpoints - Reactome	0.000102	10	48	['PSMD2', 'PSMC4', 'PSMC5', 'PSMC1', 'PSMD11', 'PSMC2', 'PSMD13', 'PSMD14', 'PSMF1', 'TP53']	http://model.geneontology.org/R-HSA-69481
17	Activation of NF-kappaB in B cells - Reactome	0.000114	11	58	['PSMD2', 'PSMC4', 'PSMC5', 'PSMC1', 'PSMD11', 'PSMC2', 'PSMD13', 'CHUK', 'PSMD14', 'PSMF1', 'MAP3K7']	http://model.geneontology.org/R-HSA-1169091
18	Cyclin E associated events during G1/S transition - Reactome	0.000133	11	59	['PSMD2', 'PSMC4', 'PSMC5', 'PSMC1', 'PSMD11', 'PSMC2', 'PSMD13', 'PSMD14', 'CDKN1A', 'PSMF1', 'CDKN1B']	http://model.geneontology.org/R-HSA-69202
19	Degradation of beta-catenin by the destruction complex - Reactome	0.000133	11	59	['PSMD2', 'PSMC4', 'PSMC5', 'PSMC1', 'PSMD11', 'PSMC2', 'PSMD13', 'PPP2R1B', 'PPP2CB', 'PSMD14', 'PSMF1']	http://model.geneontology.org/R-HSA-195253
20	NIK-->noncanonical NF-kB signaling - Reactome	0.000174	10	51	['PSMD2', 'PSMC4', 'PSMC5', 'PSMC1', 'PSMD11', 'PSMC2', 'PSMD13', 'CHUK', 'PSMD14', 'PSMF1']	http://model.geneontology.org/R-HSA-5676590
21	Regulation of expression of SLITs and ROBOs - Reactome	0.000174	10	51	['PSMD2', 'PSMC4', 'PSMC5', 'PSMC1', 'PSMD11', 'PSMC2', 'PSMD13', 'PSMD14', 'PSMF1', 'USP33']	http://model.geneontology.org/R-HSA-9010553
22	Dectin-1 mediated noncanonical NF-kB signaling - Reactome	0.000174	10	51	['PSMD2', 'PSMC4', 'PSMC5', 'PSMC1', 'PSMD11', 'PSMC2', 'PSMD13', 'CHUK', 'PSMD14', 'PSMF1']	http://model.geneontology.org/R-HSA-5607761
23	Hedgehog ligand biogenesis - Reactome	0.000206	10	52	['VCP', 'PSMD2', 'PSMC4', 'PSMC5', 'PSMC1', 'PSMD11', 'PSMC2', 'PSMD13', 'PSMD14', 'PSMF1']	http://model.geneontology.org/R-HSA-5358346
24	Orc1 removal from chromatin - Reactome	0.000206	10	52	['PSMD2', 'PSMC4', 'PSMC5', 'PSMC1', 'PSMD11', 'PSMC2', 'PSMD13', 'PSMD14', 'CDK2', 'PSMF1']	http://model.geneontology.org/R-HSA-68949
25	Resolution of Sister Chromatid Cohesion - Reactome	0.000226	15	106	['PPP2R1B', 'PPP2CB', 'CDK1', 'SEC13', 'TAOK1', 'KIF2C', 'MAD2L1', 'BUB1B', 'SPC24', 'DSN1', 'PLK1', 'NDE1', 'CDC20', 'INCENP', 'NSL1']	http://model.geneontology.org/R-HSA-2500257
26	GSK3B and BTRC:CUL1-mediated-degradation of NFE2L2 - Reactome	0.000264	9	44	['PSMD2', 'PSMC4', 'PSMC5', 'PSMC1', 'PSMD11', 'PSMC2', 'PSMD13', 'PSMD14', 'PSMF1']	http://model.geneontology.org/R-HSA-9762114
27	AUF1 (hnRNP D0) binds and destabilizes mRNA - Reactome	0.000315	9	45	['PSMD2', 'PSMC4', 'PSMC5', 'PSMC1', 'PSMD11', 'PSMC2', 'PSMD13', 'PSMD14', 'PSMF1']	http://model.geneontology.org/R-HSA-450408
28	APC/C:Cdc20 mediated degradation of mitotic proteins - Reactome	0.000315	9	45	['PSMD2', 'PSMC4', 'PSMC5', 'PSMC1', 'PSMD11', 'PSMC2', 'PSMD13', 'PSMD14', 'PSMF1']	http://model.geneontology.org/R-HSA-176409
29	Cross-presentation of soluble exogenous antigens (endosomes) - Reactome	0.000315	9	45	['PSMD2', 'PSMC4', 'PSMC5', 'PSMC1', 'PSMD11', 'PSMC2', 'PSMD13', 'PSMD14', 'PSMF1']	http://model.geneontology.org/R-HSA-1236978
30	Regulation of RUNX3 expression and activity - Reactome	0.000375	9	46	['PSMD2', 'PSMC4', 'PSMC5', 'PSMC1', 'PSMD11', 'PSMC2', 'PSMD13', 'PSMD14', 'PSMF1']	http://model.geneontology.org/R-HSA-8941858
31	Ubiquitin-dependent degradation of Cyclin D - Reactome	0.000375	9	46	['PSMD2', 'PSMC4', 'PSMC5', 'PSMC1', 'PSMD11', 'PSMC2', 'PSMD13', 'PSMD14', 'PSMF1']	http://model.geneontology.org/R-HSA-75815
32	Regulation of activated PAK-2p34 by proteasome mediated degradation - Reactome	0.000375	9	46	['PSMD2', 'PSMC4', 'PSMC5', 'PSMC1', 'PSMD11', 'PSMC2', 'PSMD13', 'PSMD14', 'PSMF1']	http://model.geneontology.org/R-HSA-211733
33	FCERI mediated NF-kB activation - Reactome	0.000389	10	56	['PSMD2', 'PSMC4', 'PSMC5', 'PSMC1', 'PSMD11', 'PSMC2', 'PSMD13', 'PSMD14', 'PSMF1', 'MAP3K7']	http://model.geneontology.org/R-HSA-2871837
34	CLEC7A (Dectin-1) signaling - Reactome	0.000389	10	56	['PSMD2', 'PSMC4', 'PSMC5', 'PSMC1', 'PSMD11', 'PSMC2', 'PSMD13', 'PSMD14', 'PSMF1', 'MAP3K7']	http://model.geneontology.org/R-HSA-5607764
35	Asymmetric localization of PCP proteins - Reactome	0.000444	9	47	['PSMD2', 'PSMC4', 'PSMC5', 'PSMC1', 'PSMD11', 'PSMC2', 'PSMD13', 'PSMD14', 'PSMF1']	http://model.geneontology.org/R-HSA-4608870
36	TNFR2 non-canonical NF-kB pathway - Reactome	0.000444	9	47	['PSMD2', 'PSMC4', 'PSMC5', 'PSMC1', 'PSMD11', 'PSMC2', 'PSMD13', 'PSMD14', 'PSMF1']	http://model.geneontology.org/R-HSA-5668541
37	Autodegradation of the E3 ubiquitin ligase COP1 - Reactome	0.000444	9	47	['PSMD2', 'PSMC4', 'PSMC5', 'PSMC1', 'PSMD11', 'PSMC2', 'PSMD13', 'PSMD14', 'PSMF1']	http://model.geneontology.org/R-HSA-349425
38	Degradation of DVL - Reactome	0.000444	9	47	['PSMD2', 'PSMC4', 'PSMC5', 'PSMC1', 'PSMD11', 'PSMC2', 'PSMD13', 'PSMD14', 'PSMF1']	http://model.geneontology.org/R-HSA-4641258
39	KEAP1-NFE2L2 pathway - Reactome	0.000451	10	57	['PSMD2', 'PSMC4', 'PSMC5', 'PSMC1', 'PSMD11', 'PSMC2', 'PSMD13', 'CSNK2A1', 'PSMD14', 'PSMF1']	http://model.geneontology.org/R-HSA-9755511
40	RUNX1 regulates transcription of genes involved in differentiation of HSCs - Reactome	0.000613	9	49	['PSMD2', 'PSMC4', 'PSMC5', 'PSMC1', 'PSMD11', 'PSMC2', 'PSMD13', 'PSMD14', 'PSMF1']	http://model.geneontology.org/R-HSA-8939236
41	Degradation of AXIN - Reactome	0.000613	9	49	['PSMD2', 'PSMC4', 'PSMC5', 'PSMC1', 'PSMD11', 'PSMC2', 'PSMD13', 'PSMD14', 'PSMF1']	http://model.geneontology.org/R-HSA-4641257
42	Oxygen-dependent proline hydroxylation of Hypoxia-inducible Factor Alpha - Reactome	0.000613	9	49	['PSMD2', 'PSMC4', 'PSMC5', 'PSMC1', 'PSMD11', 'PSMC2', 'PSMD13', 'PSMD14', 'PSMF1']	http://model.geneontology.org/R-HSA-1234176
43	Ubiquitin Mediated Degradation of Phosphorylated Cdc25A - Reactome	0.000613	9	49	['PSMD2', 'PSMC4', 'PSMC5', 'PSMC1', 'PSMD11', 'PSMC2', 'PSMD13', 'PSMD14', 'PSMF1']	http://model.geneontology.org/R-HSA-69601
44	FBXL7 down-regulates AURKA during mitotic entry and in early mitosis - Reactome	0.000715	9	50	['PSMD2', 'PSMC4', 'PSMC5', 'PSMC1', 'PSMD11', 'PSMC2', 'PSMD13', 'PSMD14', 'PSMF1']	http://model.geneontology.org/R-HSA-8854050
45	Hedgehog 'on' state - Reactome	0.000715	9	50	['PSMD2', 'PSMC4', 'PSMC5', 'PSMC1', 'PSMD11', 'PSMC2', 'PSMD13', 'PSMD14', 'PSMF1']	http://model.geneontology.org/R-HSA-5632684
46	Negative regulation of NOTCH4 signaling - Reactome	0.000831	9	51	['PSMD2', 'PSMC4', 'PSMC5', 'PSMC1', 'PSMD11', 'PSMC2', 'PSMD13', 'PSMD14', 'PSMF1']	http://model.geneontology.org/R-HSA-9604323
47	Senescence-Associated Secretory Phenotype (SASP) - Reactome	0.000840	7	32	['CDK2', 'CDKN1A', 'UBE2S', 'UBE2C', 'CDKN1B', 'UBE2E1', 'ANAPC2']	http://model.geneontology.org/R-HSA-2559582
48	Regulation of RAS by GAPs - Reactome	0.001024	10	63	['PSMD2', 'PSMC4', 'PSMC5', 'PSMC1', 'PSMD11', 'PSMC2', 'PSMD13', 'PSMD14', 'NRAS', 'PSMF1']	http://model.geneontology.org/R-HSA-5658442
49	Degradation of GLI1 by the proteasome - Reactome	0.001109	9	53	['PSMD2', 'PSMC4', 'PSMC5', 'PSMC1', 'PSMD11', 'PSMC2', 'PSMD13', 'PSMD14', 'PSMF1']	http://model.geneontology.org/R-HSA-5610780
50	ABC-family proteins mediated transport - Reactome	0.001161	10	64	['PSMD2', 'PSMC4', 'PSMC5', 'PSMC1', 'PSMD11', 'PSMC2', 'PSMD13', 'PSMD14', 'ABCC4', 'PSMF1']	http://model.geneontology.org/R-HSA-382556
51	Regulation of PTEN stability and activity - Reactome	0.001161	10	64	['PSMD2', 'PSMC4', 'PSMC5', 'PSMC1', 'PSMD11', 'PSMC2', 'PSMD13', 'CSNK2A1', 'PSMD14', 'PSMF1']	http://model.geneontology.org/R-HSA-8948751
52	Neddylation - Reactome	0.001161	10	64	['PSMD2', 'PSMC4', 'PSMC5', 'PSMC1', 'PSMD11', 'PSMC2', 'PSMD13', 'PSMD14', 'PSMF1', 'RNF7']	http://model.geneontology.org/R-HSA-8951664
53	Regulation of RUNX2 expression and activity - Reactome	0.001273	9	54	['PSMD2', 'PSMC4', 'PSMC5', 'PSMC1', 'PSMD11', 'PSMC2', 'PSMD13', 'PSMD14', 'PSMF1']	http://model.geneontology.org/R-HSA-8939902
54	Degradation of GLI2 by the proteasome - Reactome	0.001273	9	54	['PSMD2', 'PSMC4', 'PSMC5', 'PSMC1', 'PSMD11', 'PSMC2', 'PSMD13', 'PSMD14', 'PSMF1']	http://model.geneontology.org/R-HSA-5610783
55	Antigen Presentation: Folding, assembly and peptide loading of class I MHC - Reactome	0.001273	9	54	['PSMD2', 'PSMC4', 'PSMC5', 'PSMC1', 'PSMD11', 'PSMC2', 'PSMD13', 'PSMD14', 'PSMF1']	http://model.geneontology.org/R-HSA-983170
56	GLI3 is processed to GLI3R by the proteasome - Reactome	0.001273	9	54	['PSMD2', 'PSMC4', 'PSMC5', 'PSMC1', 'PSMD11', 'PSMC2', 'PSMD13', 'PSMD14', 'PSMF1']	http://model.geneontology.org/R-HSA-5610785
57	Interleukin-1 signaling - Reactome	0.001313	10	65	['PSMD2', 'PSMC4', 'PSMC5', 'PSMC1', 'PSMD11', 'PSMC2', 'PSMD13', 'PSMD14', 'PSMF1', 'MAP3K7']	http://model.geneontology.org/R-HSA-9020702
58	Synthesis of PC - Reactome	0.001317	8	44	['CSNK2A1', 'PNPLA2', 'PCYT1A', 'SLC44A1', 'GPAT4', 'GPAT3', 'ABHD3', 'LPCAT4']	http://model.geneontology.org/R-HSA-1483191
59	Hedgehog 'off' state - Reactome	0.001457	9	55	['PSMD2', 'PSMC4', 'PSMC5', 'PSMC1', 'PSMD11', 'PSMC2', 'PSMD13', 'PSMD14', 'PSMF1']	http://model.geneontology.org/R-HSA-5610787
60	Inhibition of replication initiation of damaged DNA by RB1/E2F1 - Reactome	0.001524	5	18	['PPP2R1B', 'PPP2CB', 'CDK2', 'CDKN1A', 'CDKN1B']	http://model.geneontology.org/R-HSA-113501
61	MAPK6/MAPK4 signaling - Reactome	0.001868	10	68	['PSMD2', 'PSMC4', 'PSMC5', 'PSMC1', 'PSMD11', 'PSMC2', 'PSMD13', 'CDK1', 'PSMD14', 'PSMF1']	http://model.geneontology.org/R-HSA-5687128
62	Activation of the mRNA upon binding of the cap-binding complex and eIFs, and subsequent binding to 43S - Reactome	0.002504	2	2	['EIF4A1', 'EIF4A2']	http://model.geneontology.org/R-HSA-72662
63	Translation initiation complex formation - Reactome	0.002504	2	2	['EIF4A1', 'EIF4A2']	http://model.geneontology.org/R-HSA-72649

```
In [4]: p97_weighted = enrich.enrich_wrapper('P97.csv','uniprot', FDR = 0.1, method='ncHGT', fpath = '../test_data/processed', table_number = 3)
        p97_weighted
```

```
100%|██████████████████████████████| 480/480 [00:42<00:00, 11.22it/s]
Analysis run on 338 entities from 270 out of 766 input genes
```

Out[4]: **Table SAC: P97 combined up/downregulated proteins, weighted set method, FDR = 0.1.**

	title	pval (uncorrected)	# entities in list	#entities in model	shared entities in gocam	url
0	CDK-mediated phosphorylation and removal of Cdc6 - Reactome	0.000001	14	65	['PSMD2', 'PSMC4', 'PSMC5', 'PSMC1', 'PSMD11', 'PSMC2', 'PSMD13', 'PSMD14', 'CDK2', 'PSMF1', 'UBE2S', 'UBE2C', 'UBE2E1', 'ANAPC2']	http://model.geneontology.org/R-HSA-69017
1	Autodegradation of Cdh1 by Cdh1:APC/C - Reactome	0.000001	13	59	['PSMD2', 'PSMC4', 'PSMC5', 'PSMC1', 'PSMD11', 'PSMC2', 'PSMD13', 'PSMD14', 'PSMF1', 'UBE2S', 'UBE2C', 'UBE2E1', 'ANAPC2']	http://model.geneontology.org/R-HSA-174084
2	SCF(Skp2)-mediated degradation of p27/p21 - Reactome	0.000001	12	49	['PSMD2', 'PSMC4', 'PSMC5', 'PSMC1', 'PSMD11', 'PSMC2', 'PSMD13', 'PSMD14', 'CDK2', 'PSMF1', 'CKS1B', 'set:p21,p27']	http://model.geneontology.org/R-HSA-187577
3	Assembly of the pre-replicative complex - Reactome	0.000001	13	60	['PSMD2', 'PSMC4', 'PSMC5', 'PSMC1', 'PSMD11', 'PSMC2', 'PSMD13', 'PSMD14', 'PSMF1', 'UBE2S', 'UBE2C', 'UBE2E1', 'ANAPC2']	http://model.geneontology.org/R-HSA-68867
4	Unwinding of DNA - Reactome	0.000001	6	7	['MCM3', 'MCM6', 'MCM4', 'MCM2', 'MCM7', 'MCM5']	http://model.geneontology.org/R-HSA-176974
5	Cdc20:Phospho-APC/C mediated degradation of Cyclin A - Reactome	0.000001	13	62	['PSMD2', 'PSMC4', 'PSMC5', 'PSMC1', 'PSMD11', 'PSMC2', 'PSMD13', 'PSMD14', 'PSMF1', 'UBE2S', 'UBE2C', 'UBE2E1', 'ANAPC2']	http://model.geneontology.org/R-HSA-174184
6	APC/C:Cdh1 mediated degradation of Cdc20 and other APC/C:Cdh1 targeted proteins in late mitosis/early G1 - Reactome	0.000001	13	62	['PSMD2', 'PSMC4', 'PSMC5', 'PSMC1', 'PSMD11', 'PSMC2', 'PSMD13', 'PSMD14', 'PSMF1', 'UBE2S', 'UBE2C', 'UBE2E1', 'ANAPC2']	http://model.geneontology.org/R-HSA-174178
7	APC/C:Cdc20 mediated degradation of Securin - Reactome	0.000001	13	62	['PSMD2', 'PSMC4', 'PSMC5', 'PSMC1', 'PSMD11', 'PSMC2', 'PSMD13', 'PSMD14', 'PSMF1', 'UBE2S', 'UBE2C', 'UBE2E1', 'ANAPC2']	http://model.geneontology.org/R-HSA-174154
8	Separation of Sister Chromatids - Reactome	0.000002	13	63	['PSMD2', 'PSMC4', 'PSMC5', 'PSMC1', 'PSMD11', 'PSMC2', 'PSMD13', 'PSMD14', 'PSMF1', 'UBE2S', 'UBE2C', 'UBE2E1', 'ANAPC2']	http://model.geneontology.org/R-HSA-2467813
9	The role of GTSE1 in G2/M progression after G2 checkpoint - Reactome	0.000007	11	50	['PSMD2', 'PSMC4', 'PSMC5', 'PSMC1', 'PSMD11', 'PSMC2', 'PSMD13', 'PSMD14', 'PSMF1', 'PLK1', 'TP53']	http://model.geneontology.org/R-HSA-8852276
10	Conversion from APC/C:Cdc20 to APC/C:Cdh1 in late anaphase - Reactome	0.000007	11	49	['PSMD2', 'PSMC4', 'PSMC5', 'PSMC1', 'PSMD11', 'PSMC2', 'PSMD13', 'CDK1', 'PSMD14', 'CDK2', 'PSMF1']	http://model.geneontology.org/R-HSA-176407
11	SCF-beta-TrCP mediated degradation of Emi1 - Reactome	0.000010	11	51	['PSMD2', 'PSMC4', 'PSMC5', 'PSMC1', 'PSMD11', 'PSMC2', 'PSMD13', 'CDK1', 'PSMD14', 'PSMF1', 'PLK1']	http://model.geneontology.org/R-HSA-174113
12	ER-Phagosome pathway - Reactome	0.000012	11	51	['PSMD2', 'PSMC4', 'PSMC5', 'PSMC1', 'PSMD11', 'PSMC2', 'PSMD13', 'PSMD14', 'PSMF1', 'SEC61G', 'set:SEC61 alpha']	http://model.geneontology.org/R-HSA-1236974
13	Regulation of APC/C activators between G1/S and early anaphase - Reactome	0.000015	11	52	['PSMD2', 'PSMC4', 'PSMC5', 'PSMC1', 'PSMD11', 'PSMC2', 'PSMD13', 'CDK1', 'PSMD14', 'CDK2', 'PSMF1']	http://model.geneontology.org/R-HSA-176408
14	Resolution of Sister Chromatid Cohesion - Reactome	0.000018	15	95	['CDK1', 'SEC13', 'TAOK1', 'KIF2C', 'MAD2L1', 'BUB1B', 'SPC24', 'DSN1', 'PLK1', 'NDE1', 'CDC20', 'INCENP', 'NSL1', 'set:PP2A-subunit A', 'set:PP2A-catalytic subunit C']	http://model.geneontology.org/R-HSA-2500257
15	Cyclin A:Cdk2-associated events at S phase entry - Reactome	0.000023	11	52	['PSMD2', 'PSMC4', 'PSMC5', 'PSMC1', 'PSMD11', 'PSMC2', 'PSMD13', 'PSMD14', 'CDK2', 'PSMF1', 'set:p21,p27']	http://model.geneontology.org/R-HSA-69656
16	Cyclin E associated events during G1/S transition - Reactome	0.000028	11	51	['PSMD2', 'PSMC4', 'PSMC5', 'PSMC1', 'PSMD11', 'PSMC2', 'PSMD13', 'PSMD14', 'PSMF1', 'set:p21,p27', 'set:p-T172-CDK4,p-T177-CDK6:CCND:CDKN1A,CDKN1B,(CDKN1C); p-T172-CDK4,p-T177-CDK6:CCND; (p-T172-CDK4,p-T177-CDK6:CCND:p-Y88-CDKN1B)']	http://model.geneontology.org/R-HSA-69202
17	Activation of NF-kappaB in B cells - Reactome	0.000028	11	54	['PSMD2', 'PSMC4', 'PSMC5', 'PSMC1', 'PSMD11', 'PSMC2', 'PSMD13', 'CHUK', 'PSMD14', 'PSMF1', 'MAP3K7']	http://model.geneontology.org/R-HSA-1169091
18	G2/M Checkpoints - Reactome	0.000034	10	48	['PSMD2', 'PSMC4', 'PSMC5', 'PSMC1', 'PSMD11', 'PSMC2', 'PSMD13', 'PSMD14', 'PSMF1', 'TP53']	http://model.geneontology.org/R-HSA-69481
19	Degradation of beta-catenin by the destruction complex - Reactome	0.000038	11	54	['PSMD2', 'PSMC4', 'PSMC5', 'PSMC1', 'PSMD11', 'PSMC2', 'PSMD13', 'PSMD14', 'PSMF1', 'set:PP2A-subunit A', 'set:PP2A-catalytic subunit C']	http://model.geneontology.org/R-HSA-195253
20	NIK-->noncanonical NF-kB signaling - Reactome	0.000065	10	50	['PSMD2', 'PSMC4', 'PSMC5', 'PSMC1', 'PSMD11', 'PSMC2', 'PSMD13', 'CHUK', 'PSMD14', 'PSMF1']	http://model.geneontology.org/R-HSA-5676590
21	Dectin-1 mediated noncanonical NF-kB signaling - Reactome	0.000065	10	50	['PSMD2', 'PSMC4', 'PSMC5', 'PSMC1', 'PSMD11', 'PSMC2', 'PSMD13', 'CHUK', 'PSMD14', 'PSMF1']	http://model.geneontology.org/R-HSA-5607761
22	Regulation of expression of SLITs and ROBOs - Reactome	0.000067	10	51	['PSMD2', 'PSMC4', 'PSMC5', 'PSMC1', 'PSMD11', 'PSMC2', 'PSMD13', 'PSMD14', 'PSMF1', 'USP33']	http://model.geneontology.org/R-HSA-9010553
23	Hedgehog ligand biogenesis - Reactome	0.000073	10	50	['VCP', 'PSMD2', 'PSMC4', 'PSMC5', 'PSMC1', 'PSMD11', 'PSMC2', 'PSMD13', 'PSMD14', 'PSMF1']	http://model.geneontology.org/R-HSA-5358346
24	Orc1 removal from chromatin - Reactome	0.000076	10	51	['PSMD2', 'PSMC4', 'PSMC5', 'PSMC1', 'PSMD11', 'PSMC2', 'PSMD13', 'PSMD14', 'CDK2', 'PSMF1']	http://model.geneontology.org/R-HSA-68949
25	Regulation of RAS by GAPs - Reactome	0.000130	10	48	['PSMD2', 'PSMC4', 'PSMC5', 'PSMC1', 'PSMD11', 'PSMC2', 'PSMD13', 'PSMD14', 'PSMF1', 'set:mature p21 RAS']	http://model.geneontology.org/R-HSA-5658442
26	FCERI mediated NF-kB activation - Reactome	0.000136	10	54	['PSMD2', 'PSMC4', 'PSMC5', 'PSMC1', 'PSMD11', 'PSMC2', 'PSMD13', 'PSMD14', 'PSMF1', 'MAP3K7']	http://model.geneontology.org/R-HSA-2871837
27	GSK3B and BTRC:CUL1-mediated-degradation of NFE2L2 - Reactome	0.000137	9	44	['PSMD2', 'PSMC4', 'PSMC5', 'PSMC1', 'PSMD11', 'PSMC2', 'PSMD13', 'PSMD14', 'PSMF1']	http://model.geneontology.org/R-HSA-9762114
28	CLEC7A (Dectin-1) signaling - Reactome	0.000140	10	55	['PSMD2', 'PSMC4', 'PSMC5', 'PSMC1', 'PSMD11', 'PSMC2', 'PSMD13', 'PSMD14', 'PSMF1', 'MAP3K7']	http://model.geneontology.org/R-HSA-5607764
29	AUF1 (hnRNP D0) binds and destabilizes mRNA - Reactome	0.000161	9	45	['PSMD2', 'PSMC4', 'PSMC5', 'PSMC1', 'PSMD11', 'PSMC2', 'PSMD13', 'PSMD14', 'PSMF1']	http://model.geneontology.org/R-HSA-450408
30	Cross-presentation of soluble exogenous antigens (endosomes) - Reactome	0.000161	9	45	['PSMD2', 'PSMC4', 'PSMC5', 'PSMC1', 'PSMD11', 'PSMC2', 'PSMD13', 'PSMD14', 'PSMF1']	http://model.geneontology.org/R-HSA-1236978
31	APC/C:Cdc20 mediated degradation of mitotic proteins - Reactome	0.000161	9	45	['PSMD2', 'PSMC4', 'PSMC5', 'PSMC1', 'PSMD11', 'PSMC2', 'PSMD13', 'PSMD14', 'PSMF1']	http://model.geneontology.org/R-HSA-176409
32	KEAP1-NFE2L2 pathway - Reactome	0.000174	10	55	['PSMD2', 'PSMC4', 'PSMC5', 'PSMC1', 'PSMD11', 'PSMC2', 'PSMD13', 'PSMD14', 'PSMF1', 'set:CSNK2(A1:A1/A1:A2/A2:A2)']	http://model.geneontology.org/R-HSA-9755511
33	Regulation of RUNX3 expression and activity - Reactome	0.000184	9	45	['PSMD2', 'PSMC4', 'PSMC5', 'PSMC1', 'PSMD11', 'PSMC2', 'PSMD13', 'PSMD14', 'PSMF1']	http://model.geneontology.org/R-HSA-8941858
34	Regulation of activated PAK-2p34 by proteasome mediated degradation - Reactome	0.000190	9	46	['PSMD2', 'PSMC4', 'PSMC5', 'PSMC1', 'PSMD11', 'PSMC2', 'PSMD13', 'PSMD14', 'PSMF1']	http://model.geneontology.org/R-HSA-211733
35	Ubiquitin-dependent degradation of Cyclin D - Reactome	0.000190	9	46	['PSMD2', 'PSMC4', 'PSMC5', 'PSMC1', 'PSMD11', 'PSMC2', 'PSMD13', 'PSMD14', 'PSMF1']	http://model.geneontology.org/R-HSA-75815
36	Asymmetric localization of PCP proteins - Reactome	0.000216	9	46	['PSMD2', 'PSMC4', 'PSMC5', 'PSMC1', 'PSMD11', 'PSMC2', 'PSMD13', 'PSMD14', 'PSMF1']	http://model.geneontology.org/R-HSA-4608870
37	Degradation of DVL - Reactome	0.000222	9	47	['PSMD2', 'PSMC4', 'PSMC5', 'PSMC1', 'PSMD11', 'PSMC2', 'PSMD13', 'PSMD14', 'PSMF1']	http://model.geneontology.org/R-HSA-4641258
38	Autodegradation of the E3 ubiquitin ligase COP1 - Reactome	0.000222	9	47	['PSMD2', 'PSMC4', 'PSMC5', 'PSMC1', 'PSMD11', 'PSMC2', 'PSMD13', 'PSMD14', 'PSMF1']	http://model.geneontology.org/R-HSA-349425
39	PI5P, PP2A and IER3 Regulate PI3K/AKT Signaling - Reactome	0.000251	8	13	['set:PP2A-subunit A', 'set:PIP5K1A/B', 'set:PI4K2A/2B', 'set:SYNJ/MTM(1)', 'set:Activated SRC,LCK,EGFR,INSR', 'set:Activator:PI3K', 'set:PP2A-catalytic subunit C', 'set:PIP5K1A-C']	http://model.geneontology.org/R-HSA-6811558
40	ABC-family proteins mediated transport - Reactome	0.000283	10	54	['PSMD2', 'PSMC4', 'PSMC5', 'PSMC1', 'PSMD11', 'PSMC2', 'PSMD13', 'PSMD14', 'PSMF1', 'set:ABCCs']	http://model.geneontology.org/R-HSA-382556
41	TNFR2 non-canonical NF-kB pathway - Reactome	0.000284	9	47	['PSMD2', 'PSMC4', 'PSMC5', 'PSMC1', 'PSMD11', 'PSMC2', 'PSMD13', 'PSMD14', 'PSMF1']	http://model.geneontology.org/R-HSA-5668541
42	Degradation of AXIN - Reactome	0.000292	9	48	['PSMD2', 'PSMC4', 'PSMC5', 'PSMC1', 'PSMD11', 'PSMC2', 'PSMD13', 'PSMD14', 'PSMF1']	http://model.geneontology.org/R-HSA-4641257
43	Antigen processing: Ubiquitination & Proteasome degradation - Reactome	0.000297	11	50	['PSMD2', 'PSMC4', 'PSMC5', 'PSMC1', 'PSMD11', 'PSMC2', 'PSMD13', 'PSMD14', 'PSMF1', 'set:E3 ligases in proteasomal degradation', 'set:E2 Ubiquitin-conjugating enzyme']	http://model.geneontology.org/R-HSA-983168
44	RUNX1 regulates transcription of genes involved in differentiation of HSCs - Reactome	0.000300	9	49	['PSMD2', 'PSMC4', 'PSMC5', 'PSMC1', 'PSMD11', 'PSMC2', 'PSMD13', 'PSMD14', 'PSMF1']	http://model.geneontology.org/R-HSA-8939236
45	Ubiquitin Mediated Degradation of Phosphorylated Cdc25A - Reactome	0.000300	9	49	['PSMD2', 'PSMC4', 'PSMC5', 'PSMC1', 'PSMD11', 'PSMC2', 'PSMD13', 'PSMD14', 'PSMF1']	http://model.geneontology.org/R-HSA-69601
46	Oxygen-dependent proline hydroxylation of Hypoxia-inducible Factor Alpha - Reactome	0.000329	9	48	['PSMD2', 'PSMC4', 'PSMC5', 'PSMC1', 'PSMD11', 'PSMC2', 'PSMD13', 'PSMD14', 'PSMF1']	http://model.geneontology.org/R-HSA-1234176
47	Regulation of PTEN stability and activity - Reactome	0.000329	10	56	['PSMD2', 'PSMC4', 'PSMC5', 'PSMC1', 'PSMD11', 'PSMC2', 'PSMD13', 'PSMD14', 'PSMF1', 'set:CSNK2(A1:A1/A1:A2/A2:A2)']	http://model.geneontology.org/R-HSA-8948751
48	Hedgehog 'on' state - Reactome	0.000338	9	49	['PSMD2', 'PSMC4', 'PSMC5', 'PSMC1', 'PSMD11', 'PSMC2', 'PSMD13', 'PSMD14', 'PSMF1']	http://model.geneontology.org/R-HSA-5632684
49	FBXL7 down-regulates AURKA during mitotic entry and in early mitosis - Reactome	0.000346	9	50	['PSMD2', 'PSMC4', 'PSMC5', 'PSMC1', 'PSMD11', 'PSMC2', 'PSMD13', 'PSMD14', 'PSMF1']	http://model.geneontology.org/R-HSA-8854050
50	Hedgehog 'off' state - Reactome	0.000373	9	46	['PSMD2', 'PSMC4', 'PSMC5', 'PSMC1', 'PSMD11', 'PSMC2', 'PSMD13', 'PSMD14', 'PSMF1']	http://model.geneontology.org/R-HSA-5610787
51	Negative regulation of NOTCH4 signaling - Reactome	0.000380	9	49	['PSMD2', 'PSMC4', 'PSMC5', 'PSMC1', 'PSMD11', 'PSMC2', 'PSMD13', 'PSMD14', 'PSMF1']	http://model.geneontology.org/R-HSA-9604323
52	Neddylation - Reactome	0.000394	10	62	['PSMD2', 'PSMC4', 'PSMC5', 'PSMC1', 'PSMD11', 'PSMC2', 'PSMD13', 'PSMD14', 'PSMF1', 'RNF7']	http://model.geneontology.org/R-HSA-8951664
53	Degradation of GLI1 by the proteasome - Reactome	0.000491	9	51	['PSMD2', 'PSMC4', 'PSMC5', 'PSMC1', 'PSMD11', 'PSMC2', 'PSMD13', 'PSMD14', 'PSMF1']	http://model.geneontology.org/R-HSA-5610780
54	Antigen Presentation: Folding, assembly and peptide loading of class I MHC - Reactome	0.000522	9	50	['PSMD2', 'PSMC4', 'PSMC5', 'PSMC1', 'PSMD11', 'PSMC2', 'PSMD13', 'PSMD14', 'PSMF1']	http://model.geneontology.org/R-HSA-983170
55	GLI3 is processed to GLI3R by the proteasome - Reactome	0.000561	9	52	['PSMD2', 'PSMC4', 'PSMC5', 'PSMC1', 'PSMD11', 'PSMC2', 'PSMD13', 'PSMD14', 'PSMF1']	http://model.geneontology.org/R-HSA-5610785
56	Degradation of GLI2 by the proteasome - Reactome	0.000561	9	52	['PSMD2', 'PSMC4', 'PSMC5', 'PSMC1', 'PSMD11', 'PSMC2', 'PSMD13', 'PSMD14', 'PSMF1']	http://model.geneontology.org/R-HSA-5610783
57	Regulation of RUNX2 expression and activity - Reactome	0.000595	9	54	['PSMD2', 'PSMC4', 'PSMC5', 'PSMC1', 'PSMD11', 'PSMC2', 'PSMD13', 'PSMD14', 'PSMF1']	http://model.geneontology.org/R-HSA-8939902
58	Interleukin-1 signaling - Reactome	0.000712	10	64	['PSMD2', 'PSMC4', 'PSMC5', 'PSMC1', 'PSMD11', 'PSMC2', 'PSMD13', 'PSMD14', 'PSMF1', 'MAP3K7']	http://model.geneontology.org/R-HSA-9020702
59	MAPK6/MAPK4 signaling - Reactome	0.000893	10	62	['PSMD2', 'PSMC4', 'PSMC5', 'PSMC1', 'PSMD11', 'PSMC2', 'PSMD13', 'CDK1', 'PSMD14', 'PSMF1']	http://model.geneontology.org/R-HSA-5687128
60	Downstream TCR signaling - Reactome	0.001877	12	74	['PSMD2', 'PSMC4', 'PSMC5', 'PSMC1', 'PSMD11', 'PSMC2', 'PSMD13', 'PSMD14', 'PSMF1', 'MAP3K7', 'set:Activator:PI3K', 'set:PIP5K1A-C']	http://model.geneontology.org/R-HSA-202424
61	Synthesis of PC - Reactome	0.001973	7	21	['ABHD3', 'set:LPEAT', 'set:PCYT1 dimer', 'set:CSNK2(A1:A1/A1:A2/A2:A2)', 'set:AGPAT', 'set:PNPLA2/3', 'set:CTL1-5']	http://model.geneontology.org/R-HSA-1483191
62	COPII-mediated vesicle transport - Reactome	0.002506	7	27	['SEC31A', 'SEC23A', 'TFG', 'SEC13', 'CSNK1D', 'set:SEC24', 'set:COPII cargo:receptors']	http://model.geneontology.org/R-HSA-204005

Dilated and Arrhythmogenic Cardiomyopathy

Reichart et al., 2022

https://www.science.org/doi/10.1126/science.abo1984?url_ver=Z39.88-2003&rfr_id=ori:rid:crossref.org&rfr_dat=cr_pub%20%200pubmed#supplementary-materials

snRNA seq on human left and right ventricles from healthy and diseased donors. 5 genotypes: LMNA, RBM20, PKP2, TTN, and PVneg (pathogenic variant negative)

cardiomyocytes: tables S7,8 of Reichart et al

fibroblasts: tables S16,17 of Reichart et al

smooth muscle cells: tables S23-24 of Reichart et al

Left Ventricle Cardiomyocytes

LMNA

```
In [5]: LMNA_set = enrich.enrich_wrapper('LMNA_CM_comb.csv','Gene Symbol',method='set',FDR = 0.1,fpath = '../test_data/processed/', table_number = 4)
        LMNA_set
```

```
100%|██████████████████████████████| 299/299 [00:00<00:00, 16470.28it/s]
Analysis run on 191 entities from 141 out of 689 input genes
```

Out[5]: **Table SAD: LMNA cardiomyocytes combined up/downregulated genes, unweighted set method, FDR = 0.1.**

	title	pval (uncorrected)	# entities in list	#entities in model	shared entities in gocam	url
0	Regulation of Complement cascade - Reactome	0.000004	6	11	['C1R', 'C3', 'set:CD46, CR1:C4b:C3b complexes', 'set:C3 convertases', 'set:C4 activators', 'set:Complement factor D']	http://model.geneontology.org/R-HSA-977606
1	CD28 dependent PI3K/Akt signaling - Reactome	0.000060	6	16	['PIK3R1', 'MAP3K8', 'set:PI3K-regulatory subunit', 'set:Activator:PI3K', 'set:G-protein gamma subunit', 'set:PI3K alpha, beta, gamma']	http://model.geneontology.org/R-HSA-389357

```
In [6]: LMNA_standard= enrich.enrich_wrapper('LMNA_CM_comb.csv','Gene Symbol',method='standard',FDR = 0.1,fpath = '../test_data/processed/', table_number = 5)
        LMNA_standard
```

```
100%|██████████████████████████████| 299/299 [00:00<00:00, 18158.74it/s]
```

Out[6]: **Table SAE: LMNA cardiomyocytes combined up/downregulated genes, standard method, FDR = 0.1.**

title	pval (uncorrected)	# entities in list	#entities in model	shared entities in gocam	url

```
In [7]: LMNA_weighted  = enrich.enrich_wrapper('LMNA_CM_comb.csv','Gene Symbol',method='ncHGT',FDR = 0.1,fpath = '../test_data/processed/', table_number = 6)
        LMNA_weighted
```

```
100%|██████████████████████████████| 299/299 [01:55<00:00,  2.60it/s]
Analysis run on 191 entities from 141 out of 689 input genes
```

Out[7]: **Table SAF: LMNA cardiomyocytes combined up/downregulated genes, weighted set method, FDR = 0.1.**

title	pval (uncorrected)	# entities in list	#entities in model	shared entities in gocam	url

RBM20

```
In [8]: RBM20_step = enrich.enrich_wrapper('RBM20_CM_comb.csv','Gene Symbol',method='set',FDR = 0.1,fpath = '../test_data/processed/', table_number = 7)
        RBM20_step
```

```
100%|██████████████████████████████| 207/207 [00:00<00:00, 8784.19it/s]
Analysis run on 156 entities from 115 out of 533 input genes
```

Out[8]: **Table SAG: RBM20 cardiomyocytes combined up/downregulated genes, unweighted set method, FDR = 0.1.**

title	pval (uncorrected)	# entities in list	#entities in model	shared entities in gocam	url

```
In [9]: RBM20_standard= enrich.enrich_wrapper('RBM20_CM_comb.csv','Gene Symbol',method='standard',FDR = 0.1,fpath = '../test_data/processed/',table_number = 8)
        RBM20_standard
```

```
100%|██████████████████████████████| 207/207 [00:00<00:00, 17741.97it/s]
```

Out[9]: **Table SAH: RBM20 cardiomyocytes combined up/downregulated genes, standard method, FDR = 0.1.**

	title	pval (uncorrected)	# entities in list	#entities in model	shared entities in gocam	url
0	Collagen biosynthesis and modifying enzymes - Reactome	0.000079	8	65	['COL16A1', 'COL23A1', 'COL7A1', 'COL15A1', 'TLL2', 'COL25A1', 'COL5A3', 'COL6A3']	http://model.geneontology.org/R-HSA-1650814

```
In [10]: RBM20_weighted = enrich.enrich_wrapper('RBM20_CM_comb.csv','Gene Symbol',method='ncHGT',FDR = 0.1,fpath = '../test_data/processed/',table_number = 9)
         RBM20_weighted
```

```
100%|██████████████████████████████| 207/207 [00:44<00:00,  4.65it/s]
Analysis run on 156 entities from 115 out of 533 input genes
```

Out[10]: **Table SAI: RBM20 cardiomyocytes combined up/downregulated genes, weighted set method, FDR = 0.1.**

title	pval (uncorrected)	# entities in list	#entities in model	shared entities in gocam	url

PKP2

```
In [11]: PKP2_step = enrich.enrich_wrapper('PKP2_CM_comb.csv','Gene Symbol',method='set',FDR = 0.1,fpath = '../test_data/processed/',table_number = 10)
         PKP2_step
```

```
100%|██████████████████████████████| 302/302 [00:00<00:00, 18431.40it/s]
Analysis run on 151 entities from 94 out of 403 input genes
```

Out[11]: **Table SAJ: PKP2 cardiomyocytes combined up/downregulated genes, unweighted set method, FDR = 0.1.**

	title	pval (uncorrected)	# entities in list	#entities in model	shared entities in gocam	url
0	Synthesis of PIPs at the plasma membrane - Reactome	0.000007	7	21	['set:PI3K-regulatory subunit', 'set:INPP5(2)', 'set:SYNJ/INPP5(1)', 'set:PIK3C(1)', 'set:PIK3(2)', 'set:SYNJ/MTM(1)', 'set:Activator:PI3K']	http://model.geneontology.org/R-HSA-1660499

```
In [12]: PKP2_standard = enrich.enrich_wrapper('PKP2_CM_comb.csv','Gene Symbol',method='standard',FDR = 0.1,fpath = '../test_data/processed/',table_number = 11)
         PKP2_standard
```

```
100%|██████████████████████████████| 302/302 [00:00<00:00, 18041.82it/s]
```

Out[12]: **Table SAK: PKP2 cardiomyocytes combined up/downregulated genes, standard method, FDR = 0.1.**

title	pval (uncorrected)	# entities in list	#entities in model	shared entities in gocam	url

```
In [13]: PKP2_weighted = enrich.enrich_wrapper('PKP2_CM_comb.csv','Gene Symbol',method='ncHGT',FDR = 0.1,fpath = '../test_data/processed/',table_number = 12)
         PKP2_weighted
```

```
100%|██████████████████████████████| 302/302 [01:31<00:00,  3.31it/s]
Analysis run on 151 entities from 94 out of 403 input genes
```

Out[13]: **Table SAL: PKP2 cardiomyocytes combined up/downregulated genes, weighted set method, FDR = 0.1.**

title	pval (uncorrected)	# entities in list	#entities in model	shared entities in gocam	url

TTN

```
In [14]: TTN_step = enrich.enrich_wrapper('TTN_CM_comb.csv','Gene Symbol',method='set',FDR = 0.1,fpath = '../test_data/processed/',table_number = 13)
         TTN_step
```

```
100%|██████████████████████████████| 362/362 [00:00<00:00, 18259.57it/s]
Analysis run on 193 entities from 133 out of 602 input genes
```

Out[14]: **Table SAM: TTN cardiomyocytes combined up/downregulated genes, unweighted set method, FDR = 0.1.**

	title	pval (uncorrected)	# entities in list	#entities in model	shared entities in gocam	url
0	Regulation of Complement cascade - Reactome	0.000005	6	11	['C1R', 'C3', 'set:CD46, CR1:C4b:C3b complexes', 'set:C3 convertases', 'set:C4 activators', 'set:Complement factor D']	http://model.geneontology.org/R-HSA-977606
1	CD28 dependent PI3K/Akt signaling - Reactome	0.000064	6	16	['PIK3R1', 'MAP3K8', 'set:PI3K-regulatory subunit', 'set:Activator:PI3K', 'set:G-protein gamma subunit', 'set:PI3K alpha, beta, gamma']	http://model.geneontology.org/R-HSA-389357

```
In [15]: TTN_standard = enrich.enrich_wrapper('TTN_CM_comb.csv','Gene Symbol',method='standard',FDR = 0.1,fpath = '../test_data/processed/',table_number = 14)
         TTN_standard
```

```
100%|██████████████████████████████| 362/362 [00:00<00:00, 18277.37it/s]
```

Out[15]: **Table SAN: TTN cardiomyocytes combined up/downregulated genes, standard method, FDR = 0.1.**

title	pval (uncorrected)	# entities in list	#entities in model	shared entities in gocam	url

```
In [16]: TTN_weighted = enrich.enrich_wrapper('TTN_CM_comb.csv','Gene Symbol',method='ncHGT',FDR = 0.1,fpath = '../test_data/processed/',table_number = 15)
         TTN_weighted
```

```
100%|██████████████████████████████| 362/362 [01:52<00:00,  3.21it/s]
Analysis run on 193 entities from 133 out of 602 input genes
```

Out[16]: **Table SAO: TTN cardiomyocytes combined up/downregulated genes, weighted set method, FDR = 0.1.**

title	pval (uncorrected)	# entities in list	#entities in model	shared entities in gocam	url

PVneg

```
In [17]: PVneg_step = enrich.enrich_wrapper('PVneg_CM_comb.csv','Gene Symbol',method='set',FDR = 0.1,fpath = '../test_data/processed/',table_number = 16)
         PVneg_step
```

```
100%|██████████████████████████████| 93/93 [00:00<00:00, 17857.91it/s]
Analysis run on 59 entities from 42 out of 178 input genes
```

Out[17]: **Table SAP: PVneg cardiomyocytes combined up/downregulated genes, unweighted set method, FDR = 0.1.**

title	pval (uncorrected)	# entities in list	#entities in model	shared entities in gocam	url

```
In [18]: PVneg_standard = enrich.enrich_wrapper('PVneg_CM_comb.csv','Gene Symbol',method='standard',FDR = 0.1,fpath = '../test_data/processed/',table_number = 17)
         PVneg_standard
```

```
100%|██████████████████████████████| 93/93 [00:00<00:00, 17562.82it/s]
```

Out[18]: **Table SAQ: PVneg cardiomyocytes combined up/downregulated genes, standard method, FDR = 0.1.**

title	pval (uncorrected)	# entities in list	#entities in model	shared entities in gocam	url

```
In [19]: PVneg_weighted = enrich.enrich_wrapper('PVneg_CM_comb.csv','Gene Symbol',method='ncHGT',FDR = 0.1,fpath = '../test_data/processed/',table_number = 18)
         PVneg_weighted
```

```
100%|██████████████████████████████| 93/93 [00:43<00:00,  2.12it/s]
Analysis run on 59 entities from 42 out of 178 input genes
```

Out[19]: **Table SAR: PVneg cardiomyocytes combined up/downregulated genes, weighted set method, FDR = 0.1.**

title	pval (uncorrected)	# entities in list	#entities in model	shared entities in gocam	url

Left Ventricle Fibroblasts

LMNA

```
In [20]: LMNA_step = enrich.enrich_wrapper('LMNA_FB_comb.csv','Gene Symbol',method='set',FDR = 0.1,fpath = '../test_data/processed/',table_number = 19)
         LMNA_step
```

```
100%|██████████████████████████████| 401/401 [00:00<00:00, 18418.23it/s]
Analysis run on 284 entities from 234 out of 872 input genes
```

Out[20]: **Table SAS: LMNA fibroblasts combined up/downregulated genes, unweighted set method, FDR = 0.1.**

	title	pval (uncorrected)	# entities in list	#entities in model	shared entities in gocam	url
0	Peptide chain elongation - Reactome	0.000000	20	79	['RPS17', 'RPL13A', 'RPS18', 'RPLP0', 'RPLP2', 'RPL34', 'RPS20', 'RPL27A', 'RPL7', 'RPL35', 'RPL38', 'RPLP1', 'RPS15A', 'RPL23A', 'RPS5', 'RPS19', 'RPL27', 'RPS15', 'set:RPL3,RPL3L', 'set:RPL36A,RPL36AL']	http://model.geneontology.org/R-HSA-156902
1	Eukaryotic Translation Elongation - Reactome	0.000001	20	83	['RPS17', 'RPL13A', 'RPS18', 'RPLP0', 'RPLP2', 'RPL34', 'RPS20', 'RPL27A', 'RPL7', 'RPL35', 'RPL38', 'RPLP1', 'RPS15A', 'RPL23A', 'RPS5', 'RPS19', 'RPL27', 'RPS15', 'set:RPL3,RPL3L', 'set:RPL36A,RPL36AL']	http://model.geneontology.org/R-HSA-156842
2	Regulation of Complement cascade - Reactome	0.000002	7	11	['C3', 'C1R', 'set:CD46, CR1:C4b:C3b complexes', 'set:C3 convertases', 'set:C4 activators', 'set:Activated thrombin, (ELANE)', 'set:Complement factor D']	http://model.geneontology.org/R-HSA-977606
3	G alpha (i) signalling events - Reactome	0.000083	5	8	['set:GNB', 'set:RGS1,3,4,5,6,7,8,9,10,11,12,13,14,16,17,18,19,20,21', 'set:Ligand:GPCR complexes that activate Gi', 'set:Light-sensing opsins', 'set:G alpha (i)']	http://model.geneontology.org/R-HSA-418594

```
In [21]: LMNA_standard = enrich.enrich_wrapper('LMNA_FB_comb.csv','Gene Symbol',method='standard',FDR = 0.1,fpath = '../test_data/processed/',table_number = 20)
         LMNA_standard
```

```
100%|██████████████████████████████| 401/401 [00:00<00:00, 18191.53it/s]
```

Out[21]: **Table SAT: LMNA fibroblasts combined up/downregulated genes, standard method, FDR = 0.1.**

	title	pval (uncorrected)	# entities in list	#entities in model	shared entities in gocam	url
0	Peptide chain elongation - Reactome	0.000000	20	88	['RPS17', 'RPL13A', 'RPS18', 'RPLP0', 'RPLP2', 'RPL34', 'RPS20', 'RPL27A', 'RPL7', 'RPL3', 'RPL35', 'RPL38', 'RPLP1', 'RPS15A', 'RPL23A', 'RPS5', 'RPS19', 'RPL27', 'RPL36AL', 'RPS15']	http://model.geneontology.org/R-HSA-156902
1	Eukaryotic Translation Elongation - Reactome	0.000000	20	94	['RPS17', 'RPL13A', 'RPS18', 'RPLP0', 'RPLP2', 'RPL34', 'RPS20', 'RPL27A', 'RPL7', 'RPL3', 'RPL35', 'RPL38', 'RPLP1', 'RPS15A', 'RPL23A', 'RPS5', 'RPS19', 'RPL27', 'RPL36AL', 'RPS15']	http://model.geneontology.org/R-HSA-156842
2	Formation of ATP by chemiosmotic coupling - Reactome	0.000033	14	89	['MT-ND2', 'MT-ND1', 'MT-CO1', 'MT-ND4', 'SLC25A6', 'NDUFA1', 'UQCRB', 'MT-ND6', 'MT-CYB', 'MT-ATP6', 'MT-CO2', 'NDUFA3', 'SLC25A5', 'COX7C']	http://model.geneontology.org/R-HSA-163210
3	The proton buffering model - Reactome	0.000392	11	74	['MT-ND2', 'MT-ND1', 'MT-CO1', 'MT-ND4', 'NDUFA1', 'UQCRB', 'MT-ND6', 'MT-CYB', 'MT-CO2', 'NDUFA3', 'COX7C']	http://model.geneontology.org/R-HSA-167827
4	The fatty acid cycling model - Reactome	0.000392	11	74	['MT-ND2', 'MT-ND1', 'MT-CO1', 'MT-ND4', 'NDUFA1', 'UQCRB', 'MT-ND6', 'MT-CYB', 'MT-CO2', 'NDUFA3', 'COX7C']	http://model.geneontology.org/R-HSA-167826

```
In [22]: LMNA_weighted = enrich.enrich_wrapper('LMNA_FB_comb.csv','Gene Symbol',method='ncHGT',FDR = 0.1,fpath = '../test_data/processed/',table_number = 21)
         LMNA_weighted
```

```
100%|██████████████████████████████| 401/401 [02:31<00:00,  2.64it/s]
Analysis run on 284 entities from 234 out of 872 input genes
```

Out[22]: **Table SAU: LMNA fibroblasts combined up/downregulated genes, weighted set method, FDR = 0.1.**

	title	pval (uncorrected)	# entities in list	#entities in model	shared entities in gocam	url
0	Peptide chain elongation - Reactome	0.000000	20	79	['RPS17', 'RPL13A', 'RPS18', 'RPLP0', 'RPLP2', 'RPL34', 'RPS20', 'RPL27A', 'RPL7', 'RPL35', 'RPL38', 'RPLP1', 'RPS15A', 'RPL23A', 'RPS5', 'RPS19', 'RPL27', 'RPS15', 'set:RPL3,RPL3L', 'set:RPL36A,RPL36AL']	http://model.geneontology.org/R-HSA-156902
1	Eukaryotic Translation Elongation - Reactome	0.000000	20	83	['RPS17', 'RPL13A', 'RPS18', 'RPLP0', 'RPLP2', 'RPL34', 'RPS20', 'RPL27A', 'RPL7', 'RPL35', 'RPL38', 'RPLP1', 'RPS15A', 'RPL23A', 'RPS5', 'RPS19', 'RPL27', 'RPS15', 'set:RPL3,RPL3L', 'set:RPL36A,RPL36AL']	http://model.geneontology.org/R-HSA-156842
2	Formation of ATP by chemiosmotic coupling - Reactome	0.000016	13	88	['MT-ND2', 'MT-ND1', 'MT-CO1', 'MT-ND4', 'NDUFA1', 'UQCRB', 'MT-ND6', 'MT-CYB', 'MT-ATP6', 'MT-CO2', 'NDUFA3', 'COX7C', 'set:ANT homodimer']	http://model.geneontology.org/R-HSA-163210
3	TP53 Regulates Metabolic Genes - Reactome	0.000052	10	46	['MT-CO1', 'G6PD', 'UQCRB', 'MT-CYB', 'MT-CO2', 'COX7C', 'PRDX1', 'set:AMPK gamma', 'set:Active AKT', 'set:PRDX1,2,5']	http://model.geneontology.org/R-HSA-5628897
4	The proton buffering model - Reactome	0.000071	11	70	['MT-ND2', 'MT-ND1', 'MT-CO1', 'MT-ND4', 'NDUFA1', 'UQCRB', 'MT-ND6', 'MT-CYB', 'MT-CO2', 'NDUFA3', 'COX7C']	http://model.geneontology.org/R-HSA-167827
5	The fatty acid cycling model - Reactome	0.000071	11	70	['MT-ND2', 'MT-ND1', 'MT-CO1', 'MT-ND4', 'NDUFA1', 'UQCRB', 'MT-ND6', 'MT-CYB', 'MT-CO2', 'NDUFA3', 'COX7C']	http://model.geneontology.org/R-HSA-167826
6	Regulation of Complement cascade - Reactome	0.000178	7	11	['C3', 'C1R', 'set:CD46, CR1:C4b:C3b complexes', 'set:C3 convertases', 'set:C4 activators', 'set:Activated thrombin, (ELANE)', 'set:Complement factor D']	http://model.geneontology.org/R-HSA-977606
7	Respiratory electron transport - Reactome	0.000474	11	91	['MT-ND2', 'MT-ND1', 'MT-CO1', 'MT-ND4', 'NDUFA1', 'UQCRB', 'MT-ND6', 'MT-CYB', 'MT-CO2', 'NDUFA3', 'COX7C']	http://model.geneontology.org/R-HSA-611105

RBM20

```
In [23]: RBM20_set = enrich.enrich_wrapper('RBM20_FB_comb.csv','Gene Symbol',method='set',FDR = 0.1,fpath = '../test_data/processed/',table_number = 22)
         RBM20_set
```

```
100%|██████████████████████████████| 283/283 [00:00<00:00, 18585.31it/s]
Analysis run on 193 entities from 163 out of 625 input genes
```

Out[23]: **Table SAV: RBM20 fibroblasts combined up/downregulated genes, unweighted set method, FDR = 0.1.**

	title	pval (uncorrected)	# entities in list	#entities in model	shared entities in gocam	url
0	Formation of ATP by chemiosmotic coupling - Reactome	0.000015	15	88	['MT-ND4', 'MT-ND2', 'MT-ND1', 'MT-ATP6', 'MT-ND6', 'MT-CYB', 'MT-CO1', 'UQCRB', 'MT-CO2', 'MT-ND3', 'MT-CO3', 'MT-ND5', 'NDUFA1', 'COX7C', 'set:ANT homodimer']	http://model.geneontology.org/R-HSA-163210
1	Peptide chain elongation - Reactome	0.000019	14	79	['RPS17', 'RPL13A', 'RPS18', 'RPS15A', 'RPL27A', 'RPLP2', 'RPL34', 'RPS20', 'RPLP0', 'RPS5', 'RPL7', 'RPL38', 'RPL27', 'set:RPL3,RPL3L']	http://model.geneontology.org/R-HSA-156902
2	The proton buffering model - Reactome	0.000022	13	70	['MT-ND4', 'MT-ND2', 'MT-ND1', 'MT-ND6', 'MT-CYB', 'MT-CO1', 'UQCRB', 'MT-CO2', 'MT-ND3', 'MT-CO3', 'MT-ND5', 'NDUFA1', 'COX7C']	http://model.geneontology.org/R-HSA-167827
3	The fatty acid cycling model - Reactome	0.000022	13	70	['MT-ND4', 'MT-ND2', 'MT-ND1', 'MT-ND6', 'MT-CYB', 'MT-CO1', 'UQCRB', 'MT-CO2', 'MT-ND3', 'MT-CO3', 'MT-ND5', 'NDUFA1', 'COX7C']	http://model.geneontology.org/R-HSA-167826
4	Eukaryotic Translation Elongation - Reactome	0.000034	14	83	['RPS17', 'RPL13A', 'RPS18', 'RPS15A', 'RPL27A', 'RPLP2', 'RPL34', 'RPS20', 'RPLP0', 'RPS5', 'RPL7', 'RPL38', 'RPL27', 'set:RPL3,RPL3L']	http://model.geneontology.org/R-HSA-156842
5	Regulation of Complement cascade - Reactome	0.000092	5	11	['C3', 'set:CD46, CR1:C4b:C3b complexes', 'set:C3 convertases', 'set:C4 activators', 'set:Activated thrombin, (ELANE)']	http://model.geneontology.org/R-HSA-977606
6	G alpha (i) signalling events - Reactome	0.000321	4	8	['set:RGS1,3,4,5,6,7,8,9,10,11,12,13,14,16,17,18,19,20,21', 'set:Ligand:GPCR complexes that activate Gi', 'set:G alpha (i)', 'set:Light-sensing opsins']	http://model.geneontology.org/R-HSA-418594
7	Respiratory electron transport - Reactome	0.000368	13	91	['MT-ND4', 'MT-ND2', 'MT-ND1', 'MT-ND6', 'MT-CYB', 'MT-CO1', 'UQCRB', 'MT-CO2', 'MT-ND3', 'MT-CO3', 'MT-ND5', 'NDUFA1', 'COX7C']	http://model.geneontology.org/R-HSA-611105

```
In [24]: RBM20_standard = enrich.enrich_wrapper('RBM20_FB_comb.csv','Gene Symbol',method='standard',FDR = 0.1,fpath = '../test_data/processed/',table_number = 23)
         RBM20_standard
```

```
100%|██████████████████████████████| 283/283 [00:00<00:00, 18278.23it/s]
```

Out[24]: **Table SAW: RBM20 fibroblasts combined up/downregulated genes, standard method, FDR = 0.1.**

	title	pval (uncorrected)	# entities in list	#entities in model	shared entities in gocam	url
0	Formation of ATP by chemiosmotic coupling - Reactome	0.000000	15	89	['MT-ND4', 'MT-ND2', 'MT-ND1', 'MT-ATP6', 'MT-ND6', 'MT-CYB', 'MT-CO1', 'UQCRB', 'MT-CO2', 'MT-ND3', 'MT-CO3', 'MT-ND5', 'NDUFA1', 'SLC25A6', 'COX7C']	http://model.geneontology.org/R-HSA-163210
1	The proton buffering model - Reactome	0.000000	13	74	['MT-ND4', 'MT-ND2', 'MT-ND1', 'MT-ND6', 'MT-CYB', 'MT-CO1', 'UQCRB', 'MT-CO2', 'MT-ND3', 'MT-CO3', 'MT-ND5', 'NDUFA1', 'COX7C']	http://model.geneontology.org/R-HSA-167827
2	The fatty acid cycling model - Reactome	0.000000	13	74	['MT-ND4', 'MT-ND2', 'MT-ND1', 'MT-ND6', 'MT-CYB', 'MT-CO1', 'UQCRB', 'MT-CO2', 'MT-ND3', 'MT-CO3', 'MT-ND5', 'NDUFA1', 'COX7C']	http://model.geneontology.org/R-HSA-167826
3	Peptide chain elongation - Reactome	0.000000	14	88	['RPS17', 'RPL13A', 'RPS18', 'RPS15A', 'RPL27A', 'RPLP2', 'RPL34', 'RPL3L', 'RPS20', 'RPLP0', 'RPS5', 'RPL7', 'RPL38', 'RPL27']	http://model.geneontology.org/R-HSA-156902
4	Eukaryotic Translation Elongation - Reactome	0.000001	14	94	['RPS17', 'RPL13A', 'RPS18', 'RPS15A', 'RPL27A', 'RPLP2', 'RPL34', 'RPL3L', 'RPS20', 'RPLP0', 'RPS5', 'RPL7', 'RPL38', 'RPL27']	http://model.geneontology.org/R-HSA-156842
5	Respiratory electron transport - Reactome	0.000004	13	91	['MT-ND4', 'MT-ND2', 'MT-ND1', 'MT-ND6', 'MT-CYB', 'MT-CO1', 'UQCRB', 'MT-CO2', 'MT-ND3', 'MT-CO3', 'MT-ND5', 'NDUFA1', 'COX7C']	http://model.geneontology.org/R-HSA-611105
6	Cytoprotection by HMOX1 - Reactome	0.000314	6	31	['MT-CYB', 'MT-CO1', 'UQCRB', 'MT-CO2', 'MT-CO3', 'COX7C']	http://model.geneontology.org/R-HSA-9707564
7	TP53 Regulates Metabolic Genes - Reactome	0.000416	8	59	['PRKAG3', 'MT-CYB', 'MT-CO1', 'UQCRB', 'MT-CO2', 'MT-CO3', 'COX7C', 'G6PD']	http://model.geneontology.org/R-HSA-5628897

```
In [25]: RBM20_weighted = enrich.enrich_wrapper('RBM20_FB_comb.csv','Gene Symbol',method='ncHGT',FDR = 0.1,fpath = '../test_data/processed/',table_number = 24)
         RBM20_weighted
```

```
100%|██████████████████████████████| 283/283 [02:06<00:00,  2.24it/s]
Analysis run on 193 entities from 163 out of 625 input genes
```

Out[25]: **Table SAX: RBM20 fibroblasts combined up/downregulated genes, weighted set method, FDR = 0.1.**

	title	pval (uncorrected)	# entities in list	#entities in model	shared entities in gocam	url
0	Formation of ATP by chemiosmotic coupling - Reactome	0.000000	15	88	['MT-ND4', 'MT-ND2', 'MT-ND1', 'MT-ATP6', 'MT-ND6', 'MT-CYB', 'MT-CO1', 'UQCRB', 'MT-CO2', 'MT-ND3', 'MT-CO3', 'MT-ND5', 'NDUFA1', 'COX7C', 'set:ANT homodimer']	http://model.geneontology.org/R-HSA-163210
1	Peptide chain elongation - Reactome	0.000000	14	79	['RPS17', 'RPL13A', 'RPS18', 'RPS15A', 'RPL27A', 'RPLP2', 'RPL34', 'RPS20', 'RPLP0', 'RPS5', 'RPL7', 'RPL38', 'RPL27', 'set:RPL3,RPL3L']	http://model.geneontology.org/R-HSA-156902
2	The fatty acid cycling model - Reactome	0.000000	13	70	['MT-ND4', 'MT-ND2', 'MT-ND1', 'MT-ND6', 'MT-CYB', 'MT-CO1', 'UQCRB', 'MT-CO2', 'MT-ND3', 'MT-CO3', 'MT-ND5', 'NDUFA1', 'COX7C']	http://model.geneontology.org/R-HSA-167826
3	The proton buffering model - Reactome	0.000000	13	70	['MT-ND4', 'MT-ND2', 'MT-ND1', 'MT-ND6', 'MT-CYB', 'MT-CO1', 'UQCRB', 'MT-CO2', 'MT-ND3', 'MT-CO3', 'MT-ND5', 'NDUFA1', 'COX7C']	http://model.geneontology.org/R-HSA-167827
4	Eukaryotic Translation Elongation - Reactome	0.000000	14	83	['RPS17', 'RPL13A', 'RPS18', 'RPS15A', 'RPL27A', 'RPLP2', 'RPL34', 'RPS20', 'RPLP0', 'RPS5', 'RPL7', 'RPL38', 'RPL27', 'set:RPL3,RPL3L']	http://model.geneontology.org/R-HSA-156842
5	Respiratory electron transport - Reactome	0.000000	13	91	['MT-ND4', 'MT-ND2', 'MT-ND1', 'MT-ND6', 'MT-CYB', 'MT-CO1', 'UQCRB', 'MT-CO2', 'MT-ND3', 'MT-CO3', 'MT-ND5', 'NDUFA1', 'COX7C']	http://model.geneontology.org/R-HSA-611105
6	TP53 Regulates Metabolic Genes - Reactome	0.000200	8	46	['MT-CYB', 'MT-CO1', 'UQCRB', 'MT-CO2', 'MT-CO3', 'COX7C', 'G6PD', 'set:AMPK gamma']	http://model.geneontology.org/R-HSA-5628897
7	Cytoprotection by HMOX1 - Reactome	0.000418	6	29	['MT-CYB', 'MT-CO1', 'UQCRB', 'MT-CO2', 'MT-CO3', 'COX7C']	http://model.geneontology.org/R-HSA-9707564

PKP2

```
In [26]: PKP2_set = enrich.enrich_wrapper('PKP2_FB_comb.csv','Gene Symbol',method='set',FDR = 0.1,fpath = '../test_data/processed/',table_number = 25)
         PKP2_set
```

```
100%|██████████████████████████████| 358/358 [00:00<00:00, 18474.61it/s]
Analysis run on 239 entities from 191 out of 725 input genes
```

Out[26]: **Table SAY: PKP2 fibroblasts combined up/downregulated genes, unweighted set method, FDR = 0.1.**

	title	pval (uncorrected)	# entities in list	#entities in model	shared entities in gocam	url
0	Synthesis of PIPs at the plasma membrane - Reactome	0.000000	12	21	['PTEN', 'set:PI3K-catalytic subunit', 'set:PI3K-regulatory subunit', 'set:SYNJ/INPP5(1)', 'set:PIK3C(1)', 'set:PIP4K2 dimers', 'set:PIP4K2/5K1', 'set:INPP4A/B', 'set:PIK3(2)', 'set:SYNJ/MTM(1)', 'set:SYNJs,OCRL', 'set:Activator:PI3K']	http://model.geneontology.org/R-HSA-1660499
1	PI3K/AKT Signaling - Reactome	0.000011	7	15	['PIK3R1', 'PIK3CA', 'set:PI3K-catalytic subunit', 'set:PI3K-regulatory subunit', 'set:PIP4K2 dimers', 'set:Activator:PI3K', 'set:PI3K alpha, beta, gamma']	http://model.geneontology.org/R-HSA-1257604
2	Negative regulation of the PI3K/AKT network - Reactome	0.000014	5	7	['PTEN', 'set:PI3K-catalytic subunit', 'set:PI3K-regulatory subunit', 'set:PIK3C(1)', 'set:Activator:PI3K']	http://model.geneontology.org/R-HSA-199418
3	CD28 dependent PI3K/Akt signaling - Reactome	0.000018	7	16	['PIK3R1', 'PIK3CA', 'set:PI3K-catalytic subunit', 'set:PI3K-regulatory subunit', 'set:Active AKT', 'set:Activator:PI3K', 'set:PI3K alpha, beta, gamma']	http://model.geneontology.org/R-HSA-389357
4	G alpha (i) signalling events - Reactome	0.000036	5	8	['set:RGS1,3,4,5,6,7,8,9,10,11,12,13,14,16,17,18,19,20,21', 'set:Ligand:GPCR complexes that activate Gi', 'set:G alpha (i)', 'set:Adenylate cyclase', 'set:Light-sensing opsins']	http://model.geneontology.org/R-HSA-418594
5	PI5P, PP2A and IER3 Regulate PI3K/AKT Signaling - Reactome	0.000053	6	13	['PTEN', 'set:SYNJ/INPP5(1)', 'set:PIP4K2 dimers', 'set:SYNJ/MTM(1)', 'set:Activated SRC,LCK,EGFR,INSR', 'set:Activator:PI3K']	http://model.geneontology.org/R-HSA-6811558
6	VEGFR2 mediated cell proliferation - Reactome	0.000122	8	27	['PTEN', 'SPHK1', 'set:PI3K-catalytic subunit', 'set:PI3K-regulatory subunit', 'set:G(q) alpha 11,14,15,Q', 'set:PLCdegh', 'set:ORAI dimer', 'set:G-protein alpha (q/11/14/15)']	http://model.geneontology.org/R-HSA-5218921
7	G beta:gamma signalling through PI3Kgamma - Reactome	0.000147	5	10	['PIK3R1', 'PIK3CA', 'set:PI3K-catalytic subunit', 'set:PI3K-regulatory subunit', 'set:PI3K alpha, beta, gamma']	http://model.geneontology.org/R-HSA-392451
8	DAG and IP3 signaling - Reactome	0.000441	6	18	['PTEN', 'set:PI3K-catalytic subunit', 'set:PI3K-regulatory subunit', 'set:G(q) alpha 11,14,15,Q', 'set:PLCdegh', 'set:G-protein alpha (q/11/14/15)']	http://model.geneontology.org/R-HSA-1489509
9	PI3K events in ERBB2 signaling - Reactome	0.000645	5	13	['PIK3R1', 'PIK3CA', 'EGFR', 'set:p-Y877-ERBB2 heterodimers', 'set:ERBB2 heterodimers']	http://model.geneontology.org/R-HSA-1963642
10	PI3K/AKT activation - Reactome	0.000731	4	8	['PTEN', 'NTRK1', 'set:PI3K-catalytic subunit', 'set:PI3K-regulatory subunit']	http://model.geneontology.org/R-HSA-198203
11	MET activates PI3K/AKT signaling - Reactome	0.000816	3	4	['PIK3R1', 'PIK3CA', 'HGF']	http://model.geneontology.org/R-HSA-8851907
12	VEGFR2 mediated vascular permeability - Reactome	0.000832	6	20	['PIK3R1', 'PIK3CA', 'set:PI3K-catalytic subunit', 'set:PI3K-regulatory subunit', 'set:Activator:PI3K', 'set:PI3K alpha, beta, gamma']	http://model.geneontology.org/R-HSA-5218920

```
In [27]: PKP2_standard = enrich.enrich_wrapper('PKP2_FB_comb.csv','Gene Symbol',method='standard',FDR = 0.1,fpath = '../test_data/processed/',table_number = 26)
         PKP2_standard
```

```
100%|██████████████████████████████| 358/358 [00:00<00:00, 17955.67it/s]
```

Out[27]: **Table SAZ: PKP2 fibroblasts combined up/downregulated genes, standard method, FDR = 0.1.**

title	pval (uncorrected)	# entities in list	#entities in model	shared entities in gocam	url

```
In [28]: PKP2_weighted = enrich.enrich_wrapper('PKP2_FB_comb.csv','Gene Symbol',method='ncHGT',FDR = 0.1,fpath = '../test_data/processed/',table_number = 27)
PKP2_weighted
```

```
100%|██████████████████████████████| 358/358 [02:03<00:00,  2.91it/s]
Analysis run on 239 entities from 191 out of 725 input genes
```

Out[28]: **Table SBA: PKP2 fibroblasts combined up/downregulated genes, weighted set method, FDR = 0.1.**

	title	pval (uncorrected)	# entities in list	#entities in model	shared entities in gocam	url
0	Synthesis of PIPs at the plasma membrane - Reactome	0.000000	12	21	['PTEN', 'set:PI3K-catalytic subunit', 'set:PI3K-regulatory subunit', 'set:SYNJ/INPP5(1)', 'set:PIK3C(1)', 'set:PIP4K2 dimers', 'set:PIP4K2/5K1', 'set:INPP4A/B', 'set:PIK3(2)', 'set:SYNJ/MTM(1)', 'set:SYNJs,OCRL', 'set:Activator:PI3K']	http://model.geneontology.org/R-HSA-1660499

TTN

```
In [29]: TTN_set = enrich.enrich_wrapper('TTN_FB_comb.csv','Gene Symbol',method='set',FDR = 0.1,fpath = '../test_data/processed/',table_number = 28)
TTN_set
```

```
100%|██████████████████████████████| 461/461 [00:00<00:00, 18273.33it/s]
Analysis run on 359 entities from 311 out of 1092 input genes
```

Out[29]: **Table SBB: TTN fibroblasts combined up/downregulated genes, unweighted set method, FDR = 0.1.**

	title	pval (uncorrected)	# entities in list	#entities in model	shared entities in gocam	url
0	Peptide chain elongation - Reactome	0.000000	54	79	['RPS17', 'RPL13A', 'RPS18', 'RPLP0', 'RPL34', 'RPLP2', 'RPS15A', 'RPS5', 'RPL7', 'RPL18', 'RPS20', 'RPL27A', 'RPL35', 'RPL27', 'RPS25', 'RPS14', 'RPS19', 'RPL36', 'RPS15', 'RPL38', 'RPS28', 'RPL28', 'UBA52', 'RPLP1', 'RPL21', 'RPS11', 'RPL23A', 'RPS2', 'RPS13', 'RPS6', 'RPL13', 'RPL23', 'RPL7A', 'RPS23', 'RPS27A', 'RPL41', 'RPL15', 'RPL35A', 'RPS8', 'RPL10A', 'RPL12', 'RPL37A', 'FAU', 'RPL29', 'RPL31', 'RPS9', 'RPS12', 'RPL11', 'RPL8', 'set:RPL22,RPL22L1', 'set:RPS27,RPS27L', 'set:RPS4X,RPS4Y1,RPS4Y2', 'set:RPL3,RPL3L', 'set:RPL36A,RPL36AL']	http://model.geneontology.org/R-HSA-156902
1	Eukaryotic Translation Elongation - Reactome	0.000000	55	83	['RPS17', 'RPL13A', 'RPS18', 'RPLP0', 'RPL34', 'RPLP2', 'RPS15A', 'RPS5', 'RPL7', 'RPL18', 'RPS20', 'RPL27A', 'RPL35', 'RPL27', 'RPS25', 'RPS14', 'RPS19', 'RPL36', 'RPS15', 'RPL38', 'RPS28', 'RPL28', 'UBA52', 'RPLP1', 'RPL21', 'RPS11', 'RPL23A', 'RPS2', 'RPS13', 'RPS6', 'RPL13', 'RPL23', 'RPL7A', 'RPS23', 'RPS27A', 'RPL41', 'RPL15', 'RPL35A', 'RPS8', 'RPL10A', 'RPL12', 'RPL37A', 'FAU', 'RPL29', 'RPL31', 'RPS9', 'RPS12', 'RPL11', 'RPL8', 'set:EEF1A1-like proteins', 'set:RPL22,RPL22L1', 'set:RPS27,RPS27L', 'set:RPS4X,RPS4Y1,RPS4Y2', 'set:RPL3,RPL3L', 'set:RPL36A,RPL36AL']	http://model.geneontology.org/R-HSA-156842
2	Regulation of Complement cascade - Reactome	0.000011	7	11	['C3', 'C1R', 'set:CD46, CR1:C4b:C3b complexes', 'set:C3 convertases', 'set:C4 activators', 'set:Activated thrombin, (ELANE)', 'set:Complement factor D']	http://model.geneontology.org/R-HSA-977606
3	G alpha (i) signalling events - Reactome	0.000012	6	8	['set:GNB', 'set:RGS1,3,4,5,6,7,8,9,10,11,12,13,14,16,17,18,19,20,21', 'set:Ligand:GPCR complexes that activate Gi', 'set:G alpha (i)', 'set:Adenylate cyclase', 'set:Light-sensing opsins']	http://model.geneontology.org/R-HSA-418594
4	Collagen biosynthesis and modifying enzymes - Reactome	0.000297	6	12	['PPIB', 'set:Procollagen C-proteinases', 'set:Prolyl 3-hydroxylases', 'set:COLGALT1,COLGALT2', 'set:4-Hyp collagen propeptides', 'set:Procollagen N-proteinases']	http://model.geneontology.org/R-HSA-1650814

```
In [30]: TTN_standard = enrich.enrich_wrapper('TTN_FB_comb.csv','Gene Symbol',method='standard',FDR = 0.1,fpath = '../test_data/processed/',table_number = 29)
TTN_standard
```

```
100%|██████████████████████████████| 461/461 [00:00<00:00, 17019.40it/s]
```

Out[30]: **Table SBC: TTN fibroblasts combined up/downregulated genes, standard method, FDR = 0.1.**

	title	pval (uncorrected)	# entities in list	#entities in model	shared entities in gocam	url
0	Eukaryotic Translation Elongation - Reactome	0.000000	56	94	['EEF1A2', 'RPS17', 'RPL13A', 'RPS18', 'RPLP0', 'RPL34', 'RPLP2', 'RPL3', 'RPS15A', 'RPS5', 'RPL7', 'RPL18', 'RPS20', 'RPS27', 'RPL27A', 'RPL35', 'RPL27', 'RPS25', 'RPS14', 'RPS19', 'RPL36', 'RPS15', 'RPL38', 'RPS28', 'RPL28', 'UBA52', 'RPLP1', 'RPL21', 'RPS11', 'RPL23A', 'RPS2', 'RPS13', 'RPS6', 'RPL13', 'RPL23', 'RPL7A', 'RPS23', 'RPS4X', 'RPS27A', 'RPL41', 'RPL15', 'RPL35A', 'RPS8', 'RPL10A', 'RPL36AL', 'RPL22L1', 'RPL12', 'RPL37A', 'FAU', 'RPL29', 'EEF1A1', 'RPL31', 'RPS9', 'RPS12', 'RPL11', 'RPL8']	http://model.geneontology.org/R-HSA-156842
1	Peptide chain elongation - Reactome	0.000000	54	88	['RPS17', 'RPL13A', 'RPS18', 'RPLP0', 'RPL34', 'RPLP2', 'RPL3', 'RPS15A', 'RPS5', 'RPL7', 'RPL18', 'RPS20', 'RPS27', 'RPL27A', 'RPL35', 'RPL27', 'RPS25', 'RPS14', 'RPS19', 'RPL36', 'RPS15', 'RPL38', 'RPS28', 'RPL28', 'UBA52', 'RPLP1', 'RPL21', 'RPS11', 'RPL23A', 'RPS2', 'RPS13', 'RPS6', 'RPL13', 'RPL23', 'RPL7A', 'RPS23', 'RPS4X', 'RPS27A', 'RPL41', 'RPL15', 'RPL35A', 'RPS8', 'RPL10A', 'RPL36AL', 'RPL22L1', 'RPL12', 'RPL37A', 'FAU', 'RPL29', 'RPL31', 'RPS9', 'RPS12', 'RPL11', 'RPL8']	http://model.geneontology.org/R-HSA-156902

```
In [31]: TTN_weighted = enrich.enrich_wrapper('TTN_FB_comb.csv','Gene Symbol',method='ncHGT',FDR = 0.1,fpath = '../test_data/processed/',table_number = 30)
TTN_weighted
```

```
100%|██████████████████████████████| 461/461 [02:47<00:00,  2.76it/s]
Analysis run on 359 entities from 311 out of 1092 input genes
```

Out[31]: **Table SBD: TTN fibroblasts combined up/downregulated genes, weighted set method, FDR = 0.1.**

	title	pval (uncorrected)	# entities in list	#entities in model	shared entities in gocam	url
0	Peptide chain elongation - Reactome	0.000000	54	79	['RPS17', 'RPL13A', 'RPS18', 'RPLP0', 'RPL34', 'RPLP2', 'RPS15A', 'RPS5', 'RPL7', 'RPL18', 'RPS20', 'RPL27A', 'RPL35', 'RPL27', 'RPS25', 'RPS14', 'RPS19', 'RPL36', 'RPS15', 'RPL38', 'RPS28', 'RPL28', 'UBA52', 'RPLP1', 'RPL21', 'RPS11', 'RPL23A', 'RPS2', 'RPS13', 'RPS6', 'RPL13', 'RPL23', 'RPL7A', 'RPS23', 'RPS27A', 'RPL41', 'RPL15', 'RPL35A', 'RPS8', 'RPL10A', 'RPL12', 'RPL37A', 'FAU', 'RPL29', 'RPL31', 'RPS9', 'RPS12', 'RPL11', 'RPL8', 'set:RPL22,RPL22L1', 'set:RPS27,RPS27L', 'set:RPS4X,RPS4Y1,RPS4Y2', 'set:RPL3,RPL3L', 'set:RPL36A,RPL36AL']	http://model.geneontology.org/R-HSA-156902
1	Eukaryotic Translation Elongation - Reactome	0.000000	55	83	['RPS17', 'RPL13A', 'RPS18', 'RPLP0', 'RPL34', 'RPLP2', 'RPS15A', 'RPS5', 'RPL7', 'RPL18', 'RPS20', 'RPL27A', 'RPL35', 'RPL27', 'RPS25', 'RPS14', 'RPS19', 'RPL36', 'RPS15', 'RPL38', 'RPS28', 'RPL28', 'UBA52', 'RPLP1', 'RPL21', 'RPS11', 'RPL23A', 'RPS2', 'RPS13', 'RPS6', 'RPL13', 'RPL23', 'RPL7A', 'RPS23', 'RPS27A', 'RPL41', 'RPL15', 'RPL35A', 'RPS8', 'RPL10A', 'RPL12', 'RPL37A', 'FAU', 'RPL29', 'RPL31', 'RPS9', 'RPS12', 'RPL11', 'RPL8', 'set:EEF1A1-like proteins', 'set:RPL22,RPL22L1', 'set:RPS27,RPS27L', 'set:RPS4X,RPS4Y1,RPS4Y2', 'set:RPL3,RPL3L', 'set:RPL36A,RPL36AL']	http://model.geneontology.org/R-HSA-156842

PVneg

```
In [32]: PVneg_set = enrich.enrich_wrapper('PVneg_FB_comb.csv','Gene Symbol',method='set',FDR = 0.1 ,fpath = '../test_data/processed/',table_number = 31)
PVneg_set
```

```
100%|██████████████████████████████| 452/452 [00:00<00:00, 18584.70it/s]
Analysis run on 396 entities from 338 out of 1135 input genes
```

Out[32]: **Table SBE: PVneg fibroblasts combined up/downregulated genes, unweighted set method, FDR = 0.1.**

	title	pval (uncorrected)	# entities in list	#entities in model	shared entities in gocam	url
0	Peptide chain elongation - Reactome	0.000000	50	79	['RPS17', 'RPL13A', 'RPLP0', 'RPS18', 'RPLP2', 'RPL27A', 'RPS15A', 'RPS2', 'RPL7', 'RPL35', 'RPLP1', 'RPS20', 'RPS28', 'RPS5', 'RPS14', 'RPL34', 'RPL15', 'RPL23A', 'FAU', 'RPL21', 'RPL27', 'RPS25', 'RPL12', 'RPL10A', 'RPS15', 'RPS19', 'RPS16', 'RPL18', 'RPS21', 'RPL13', 'UBA52', 'RPL41', 'RPL7A', 'RPS11', 'RPS8', 'RPL29', 'RPS6', 'RPL8', 'RPS23', 'RPS9', 'RPSA', 'RPL4', 'RPS13', 'RPS27A', 'RPL19', 'set:RPL10,RPL10L', 'set:RPL22,RPL22L1', 'set:RPS27,RPS27L', 'set:RPL3,RPL3L', 'set:RPL36A,RPL36AL']	http://model.geneontology.org/R-HSA-156902
1	Eukaryotic Translation Elongation - Reactome	0.000000	51	83	['RPS17', 'RPL13A', 'RPLP0', 'RPS18', 'RPLP2', 'RPL27A', 'RPS15A', 'RPS2', 'RPL7', 'RPL35', 'RPLP1', 'RPS20', 'RPS28', 'RPS5', 'RPS14', 'RPL34', 'RPL15', 'RPL23A', 'FAU', 'RPL21', 'RPL27', 'RPS25', 'RPL12', 'RPL10A', 'RPS15', 'RPS19', 'RPS16', 'RPL18', 'RPS21', 'RPL13', 'UBA52', 'RPL41', 'RPL7A', 'RPS11', 'RPS8', 'RPL29', 'RPS6', 'EEF1B2', 'RPL8', 'RPS23', 'RPS9', 'RPSA', 'RPL4', 'RPS13', 'RPS27A', 'RPL19', 'set:RPL10,RPL10L', 'set:RPL22,RPL22L1', 'set:RPS27,RPS27L', 'set:RPL3,RPL3L', 'set:RPL36A,RPL36AL']	http://model.geneontology.org/R-HSA-156842
2	Formation of ATP by chemiosmotic coupling - Reactome	0.000000	29	88	['MT-CO1', 'MT-ND4', 'MT-ND2', 'ATP5MC1', 'CYC1', 'COX5B', 'MT-CYB', 'UQCRB', 'MT-ATP6', 'ATP5MC3', 'COX7C', 'NDUFA1', 'MT-CO2', 'MT-CO3', 'MT-ND1', 'UQCRFS1', 'NDUFA3', 'NDUFA4', 'NDUFA6', 'ATP5ME', 'UQCRQ', 'ATP5F1D', 'UQCR10', 'MT-ND6', 'NDUFB10', 'NDUFA2', 'COX4I1', 'COX6B1', 'set:ANT homodimer']	http://model.geneontology.org/R-HSA-163210
3	Cytoprotection by HMOX1 - Reactome	0.000000	15	29	['MT-CO1', 'CYC1', 'COX5B', 'MT-CYB', 'UQCRB', 'COX7C', 'MT-CO2', 'MT-CO3', 'UQCRFS1', 'NDUFA4', 'UQCRQ', 'UQCR10', 'COX4I1', 'COX6B1', 'set:BLVRA:Zn2+,BLVRB']	http://model.geneontology.org/R-HSA-9707564
4	The proton buffering model - Reactome	0.000000	23	70	['MT-CO1', 'MT-ND4', 'MT-ND2', 'CYC1', 'COX5B', 'MT-CYB', 'UQCRB', 'COX7C', 'NDUFA1', 'MT-CO2', 'MT-CO3', 'MT-ND1', 'UQCRFS1', 'NDUFA3', 'NDUFA4', 'NDUFA6', 'UQCRQ', 'UQCR10', 'MT-ND6', 'NDUFB10', 'NDUFA2', 'COX4I1', 'COX6B1']	http://model.geneontology.org/R-HSA-167827
5	The fatty acid cycling model - Reactome	0.000000	23	70	['MT-CO1', 'MT-ND4', 'MT-ND2', 'CYC1', 'COX5B', 'MT-CYB', 'UQCRB', 'COX7C', 'NDUFA1', 'MT-CO2', 'MT-CO3', 'MT-ND1', 'UQCRFS1', 'NDUFA3', 'NDUFA4', 'NDUFA6', 'UQCRQ', 'UQCR10', 'MT-ND6', 'NDUFB10', 'NDUFA2', 'COX4I1', 'COX6B1']	http://model.geneontology.org/R-HSA-167826
6	TP53 Regulates Metabolic Genes - Reactome	0.000000	18	46	['G6PD', 'MT-CO1', 'CYC1', 'COX5B', 'MT-CYB', 'UQCRB', 'COX7C', 'PRDX1', 'MT-CO2', 'MT-CO3', 'UQCRFS1', 'NDUFA4', 'UQCRQ', 'UQCR10', 'COX4I1', 'COX6B1', 'set:AMPK gamma', 'set:PRDX1,2,5']	http://model.geneontology.org/R-HSA-5628897
7	Respiratory electron transport - Reactome	0.000001	25	91	['MT-CO1', 'MT-ND4', 'MT-ND2', 'CYC1', 'COX5B', 'MT-CYB', 'UQCRB', 'COX7C', 'NDUFA1', 'MT-CO2', 'MT-CO3', 'MT-ND1', 'UQCRFS1', 'NDUFA3', 'NDUFA4', 'NDUFA6', 'UQCRQ', 'UQCR10', 'ACADVL', 'MT-ND6', 'NDUFB10', 'NDUFA2', 'IDH3B', 'COX4I1', 'COX6B1']	http://model.geneontology.org/R-HSA-611105
8	Regulation of Complement cascade - Reactome	0.000021	7	11	['C3', 'C1R', 'set:CD46, CR1:C4b:C3b complexes', 'set:C3 convertases', 'set:C4 activators', 'set:Activated thrombin, (ELANE)', 'set:Complement factor D']	http://model.geneontology.org/R-HSA-977606
9	Collagen biosynthesis and modifying enzymes - Reactome	0.000510	6	12	['PPIB', 'set:Procollagen C-proteinases', 'set:Prolyl 3-hydroxylases', 'set:COLGALT1,COLGALT2', 'set:4-Hyp collagen propeptides', 'set:Procollagen N-proteinases']	http://model.geneontology.org/R-HSA-1650814

```
In [33]: PVneg_standard = enrich.enrich_wrapper('PVneg_FB_comb.csv','Gene Symbol',method='standard',FDR = 0.1 ,fpath = '../test_data/processed/',table_number = 32)
PVneg_standard
```

```
100%|██████████████████████████████| 452/452 [00:00<00:00, 18319.81it/s]
```

Out[33]: **Table SBF: PVneg fibroblasts combined up/downregulated genes, standard method, FDR = 0.1.**

	title	pval (uncorrected)	# entities in list	#entities in model	shared entities in gocam	url
0	Peptide chain elongation - Reactome	0.000000	51	88	['RPS17', 'RPL13A', 'RPLP0', 'RPS18', 'RPLP2', 'RPL27A', 'RPS15A', 'RPS2', 'RPL7', 'RPL36AL', 'RPL35', 'RPLP1', 'RPS20', 'RPL3L', 'RPS28', 'RPS5', 'RPL3', 'RPS14', 'RPL34', 'RPL15', 'RPL23A', 'RPS27', 'FAU', 'RPL21', 'RPL27', 'RPS25', 'RPL12', 'RPL10A', 'RPS15', 'RPS19', 'RPS16', 'RPL10', 'RPL18', 'RPS21', 'RPL13', 'UBA52', 'RPL41', 'RPL7A', 'RPS11', 'RPS8', 'RPL29', 'RPS6', 'RPL8', 'RPS23', 'RPS9', 'RPSA', 'RPL4', 'RPS13', 'RPS27A', 'RPL22L1', 'RPL19']	http://model.geneontology.org/R-HSA-156902
1	Eukaryotic Translation Elongation - Reactome	0.000000	52	94	['RPS17', 'RPL13A', 'RPLP0', 'RPS18', 'RPLP2', 'RPL27A', 'RPS15A', 'RPS2', 'RPL7', 'RPL36AL', 'RPL35', 'RPLP1', 'RPS20', 'RPL3L', 'RPS28', 'RPS5', 'RPL3', 'RPS14', 'RPL34', 'RPL15', 'RPL23A', 'RPS27', 'FAU', 'RPL21', 'RPL27', 'RPS25', 'RPL12', 'RPL10A', 'RPS15', 'RPS19', 'RPS16', 'RPL10', 'RPL18', 'RPS21', 'RPL13', 'UBA52', 'RPL41', 'RPL7A', 'RPS11', 'RPS8', 'RPL29', 'RPS6', 'EEF1B2', 'RPL8', 'RPS23', 'RPS9', 'RPSA', 'RPL4', 'RPS13', 'RPS27A', 'RPL22L1', 'RPL19']	http://model.geneontology.org/R-HSA-156842
2	Formation of ATP by chemiosmotic coupling - Reactome	0.000000	30	89	['MT-CO1', 'MT-ND4', 'SLC25A6', 'MT-ND2', 'ATP5MC1', 'CYC1', 'COX5B', 'MT-CYB', 'UQCRB', 'MT-ATP6', 'ATP5MC3', 'COX7C', 'SLC25A5', 'NDUFA1', 'MT-CO2', 'MT-CO3', 'MT-ND1', 'UQCRFS1', 'NDUFA3', 'NDUFA4', 'NDUFA6', 'ATP5ME', 'UQCRQ', 'ATP5F1D', 'UQCR10', 'MT-ND6', 'NDUFB10', 'NDUFA2', 'COX4I1', 'COX6B1']	http://model.geneontology.org/R-HSA-163210
3	The proton buffering model - Reactome	0.000000	23	74	['MT-CO1', 'MT-ND4', 'MT-ND2', 'CYC1', 'COX5B', 'MT-CYB', 'UQCRB', 'COX7C', 'NDUFA1', 'MT-CO2', 'MT-CO3', 'MT-ND1', 'UQCRFS1', 'NDUFA3', 'NDUFA4', 'NDUFA6', 'UQCRQ', 'UQCR10', 'MT-ND6', 'NDUFB10', 'NDUFA2', 'COX4I1', 'COX6B1']	http://model.geneontology.org/R-HSA-167827
4	The fatty acid cycling model - Reactome	0.000000	23	74	['MT-CO1', 'MT-ND4', 'MT-ND2', 'CYC1', 'COX5B', 'MT-CYB', 'UQCRB', 'COX7C', 'NDUFA1', 'MT-CO2', 'MT-CO3', 'MT-ND1', 'UQCRFS1', 'NDUFA3', 'NDUFA4', 'NDUFA6', 'UQCRQ', 'UQCR10', 'MT-ND6', 'NDUFB10', 'NDUFA2', 'COX4I1', 'COX6B1']	http://model.geneontology.org/R-HSA-167826
5	Cytoprotection by HMOX1 - Reactome	0.000000	15	31	['MT-CO1', 'CYC1', 'COX5B', 'MT-CYB', 'UQCRB', 'COX7C', 'BLVRB', 'MT-CO2', 'MT-CO3', 'UQCRFS1', 'NDUFA4', 'UQCRQ', 'UQCR10', 'COX4I1', 'COX6B1']	http://model.geneontology.org/R-HSA-9707564
6	Respiratory electron transport - Reactome	0.000000	25	91	['MT-CO1', 'MT-ND4', 'MT-ND2', 'CYC1', 'COX5B', 'MT-CYB', 'UQCRB', 'COX7C', 'NDUFA1', 'MT-CO2', 'MT-CO3', 'MT-ND1', 'UQCRFS1', 'NDUFA3', 'NDUFA4', 'NDUFA6', 'UQCRQ', 'UQCR10', 'ACADVL', 'MT-ND6', 'NDUFB10', 'NDUFA2', 'IDH3B', 'COX4I1', 'COX6B1']	http://model.geneontology.org/R-HSA-611105
7	TP53 Regulates Metabolic Genes - Reactome	0.000000	17	59	['PRKAG3', 'G6PD', 'MT-CO1', 'CYC1', 'COX5B', 'MT-CYB', 'UQCRB', 'COX7C', 'PRDX1', 'MT-CO2', 'MT-CO3', 'UQCRFS1', 'NDUFA4', 'UQCRQ', 'UQCR10', 'COX4I1', 'COX6B1']	http://model.geneontology.org/R-HSA-5628897

```
In [34]: PVneg_weighted = enrich.enrich_wrapper('PVneg_FB_comb.csv','Gene Symbol',method='ncHGT',FDR = 0.1 ,fpath = '../test_data/processed/',table_number = 33)
PVneg_weighted
```

```
100%|██████████████████████████████| 452/452 [02:53<00:00,  2.61it/s]
Analysis run on 396 entities from 338 out of 1135 input genes
```

Out[34]: **Table SBG: PVneg fibroblasts combined up/downregulated genes, weighted set method, FDR = 0.1.**

	title	pval (uncorrected)	# entities in list	#entities in model	shared entities in gocam	url
0	Peptide chain elongation - Reactome	0.000000	50	79	['RPS17', 'RPL13A', 'RPLP0', 'RPS18', 'RPLP2', 'RPL27A', 'RPS15A', 'RPS2', 'RPL7', 'RPL35', 'RPLP1', 'RPS20', 'RPS28', 'RPS5', 'RPS14', 'RPL34', 'RPL15', 'RPL23A', 'FAU', 'RPL21', 'RPL27', 'RPS25', 'RPL12', 'RPL10A', 'RPS15', 'RPS19', 'RPS16', 'RPL18', 'RPS21', 'RPL13', 'UBA52', 'RPL41', 'RPL7A', 'RPS11', 'RPS8', 'RPL29', 'RPS6', 'RPL8', 'RPS23', 'RPS9', 'RPSA', 'RPL4', 'RPS13', 'RPS27A', 'RPL19', 'set:RPL10,RPL10L', 'set:RPL22,RPL22L1', 'set:RPS27,RPS27L', 'set:RPL3,RPL3L', 'set:RPL36A,RPL36AL']	http://model.geneontology.org/R-HSA-156902
1	Eukaryotic Translation Elongation - Reactome	0.000000	51	83	['RPS17', 'RPL13A', 'RPLP0', 'RPS18', 'RPLP2', 'RPL27A', 'RPS15A', 'RPS2', 'RPL7', 'RPL35', 'RPLP1', 'RPS20', 'RPS28', 'RPS5', 'RPS14', 'RPL34', 'RPL15', 'RPL23A', 'FAU', 'RPL21', 'RPL27', 'RPS25', 'RPL12', 'RPL10A', 'RPS15', 'RPS19', 'RPS16', 'RPL18', 'RPS21', 'RPL13', 'UBA52', 'RPL41', 'RPL7A', 'RPS11', 'RPS8', 'RPL29', 'RPS6', 'EEF1B2', 'RPL8', 'RPS23', 'RPS9', 'RPSA', 'RPL4', 'RPS13', 'RPS27A', 'RPL19', 'set:RPL10,RPL10L', 'set:RPL22,RPL22L1', 'set:RPS27,RPS27L', 'set:RPL3,RPL3L', 'set:RPL36A,RPL36AL']	http://model.geneontology.org/R-HSA-156842
2	Formation of ATP by chemiosmotic coupling - Reactome	0.000000	29	88	['MT-CO1', 'MT-ND4', 'MT-ND2', 'ATP5MC1', 'CYC1', 'COX5B', 'MT-CYB', 'UQCRB', 'MT-ATP6', 'ATP5MC3', 'COX7C', 'NDUFA1', 'MT-CO2', 'MT-CO3', 'MT-ND1', 'UQCRFS1', 'NDUFA3', 'NDUFA4', 'NDUFA6', 'ATP5ME', 'UQCRQ', 'ATP5F1D', 'UQCR10', 'MT-ND6', 'NDUFB10', 'NDUFA2', 'COX4I1', 'COX6B1', 'set:ANT homodimer']	http://model.geneontology.org/R-HSA-163210
3	The proton buffering model - Reactome	0.000000	23	70	['MT-CO1', 'MT-ND4', 'MT-ND2', 'CYC1', 'COX5B', 'MT-CYB', 'UQCRB', 'COX7C', 'NDUFA1', 'MT-CO2', 'MT-CO3', 'MT-ND1', 'UQCRFS1', 'NDUFA3', 'NDUFA4', 'NDUFA6', 'UQCRQ', 'UQCR10', 'MT-ND6', 'NDUFB10', 'NDUFA2', 'COX4I1', 'COX6B1']	http://model.geneontology.org/R-HSA-167827
4	The fatty acid cycling model - Reactome	0.000000	23	70	['MT-CO1', 'MT-ND4', 'MT-ND2', 'CYC1', 'COX5B', 'MT-CYB', 'UQCRB', 'COX7C', 'NDUFA1', 'MT-CO2', 'MT-CO3', 'MT-ND1', 'UQCRFS1', 'NDUFA3', 'NDUFA4', 'NDUFA6', 'UQCRQ', 'UQCR10', 'MT-ND6', 'NDUFB10', 'NDUFA2', 'COX4I1', 'COX6B1']	http://model.geneontology.org/R-HSA-167826
5	Respiratory electron transport - Reactome	0.000000	25	91	['MT-CO1', 'MT-ND4', 'MT-ND2', 'CYC1', 'COX5B', 'MT-CYB', 'UQCRB', 'COX7C', 'NDUFA1', 'MT-CO2', 'MT-CO3', 'MT-ND1', 'UQCRFS1', 'NDUFA3', 'NDUFA4', 'NDUFA6', 'UQCRQ', 'UQCR10', 'ACADVL', 'MT-ND6', 'NDUFB10', 'NDUFA2', 'IDH3B', 'COX4I1', 'COX6B1']	http://model.geneontology.org/R-HSA-611105
6	Cytoprotection by HMOX1 - Reactome	0.000000	15	29	['MT-CO1', 'CYC1', 'COX5B', 'MT-CYB', 'UQCRB', 'COX7C', 'MT-CO2', 'MT-CO3', 'UQCRFS1', 'NDUFA4', 'UQCRQ', 'UQCR10', 'COX4I1', 'COX6B1', 'set:BLVRA:Zn2+,BLVRB']	http://model.geneontology.org/R-HSA-9707564
7	TP53 Regulates Metabolic Genes - Reactome	0.000000	18	46	['G6PD', 'MT-CO1', 'CYC1', 'COX5B', 'MT-CYB', 'UQCRB', 'COX7C', 'PRDX1', 'MT-CO2', 'MT-CO3', 'UQCRFS1', 'NDUFA4', 'UQCRQ', 'UQCR10', 'COX4I1', 'COX6B1', 'set:AMPK gamma', 'set:PRDX1,2,5']	http://model.geneontology.org/R-HSA-5628897

Left Ventricle Mural Smooth Muscle Cells

LMNA

```
In [35]: LMNA_set = enrich.enrich_wrapper('LMNA_SMC_comb.csv','Gene Symbol',method='set',FDR = 0.1,fpath = '../test_data/processed/',table_number = 34)
LMNA_set
```

```
100%|██████████████████████████████| 326/326 [00:00<00:00, 18469.37it/s]
Analysis run on 210 entities from 161 out of 633 input genes
```

Out[35]: **Table SBH: LMNA smooth muscle cells combined up/downregulated genes, unweighted set method, FDR = 0.1.**

	title	pval (uncorrected)	# entities in list	#entities in model	shared entities in gocam	url
0	Eukaryotic Translation Elongation - Reactome	0.000004	16	83	['RPL13A', 'RPLP2', 'RPS18', 'RPL34', 'RPLP0', 'RPL7', 'RPS15A', 'RPS20', 'RPL23A', 'RPS15', 'RPL27A', 'UBA52', 'RPL35', 'RPS5', 'set:EEF1A1-like proteins', 'set:RPL3,RPL3L']	http://model.geneontology.org/R-HSA-156842
1	Regulation of Complement cascade - Reactome	0.000007	6	11	['C3', 'C1R', 'set:CD46, CR1:C4b:C3b complexes', 'set:C3 convertases', 'set:C4 activators', 'set:Complement factor D']	http://model.geneontology.org/R-HSA-977606
2	Peptide chain elongation - Reactome	0.000011	15	79	['RPL13A', 'RPLP2', 'RPS18', 'RPL34', 'RPLP0', 'RPL7', 'RPS15A', 'RPS20', 'RPL23A', 'RPS15', 'RPL27A', 'UBA52', 'RPL35', 'RPS5', 'set:RPL3,RPL3L']	http://model.geneontology.org/R-HSA-156902
3	Acyl chain remodelling of PC - Reactome	0.000041	5	9	['set:LPCAT', 'set:PLA2(5)', 'set:PLA2(16)', 'set:PLA2(7)', 'set:PLA2(6)']	http://model.geneontology.org/R-HSA-1482788
4	Acyl chain remodelling of PE - Reactome	0.000079	5	10	['set:LPEAT', 'set:PLA2(16)', 'set:PLA2(2)', 'set:PLA2(3)', 'set:PLA2(4)']	http://model.geneontology.org/R-HSA-1482839
5	Hydrolysis of LPC - Reactome	0.000232	4	7	['set:PLA2(5)', 'set:PLA2(7)', 'set:PLA2(6)', 'set:PLA2(8)']	http://model.geneontology.org/R-HSA-1483115

```
In [36]: LMNA_standard = enrich.enrich_wrapper('LMNA_SMC_comb.csv','Gene Symbol',method='standard',FDR = 0.1 ,fpath = '../test_data/processed/',table_number = 35)
LMNA_standard
```

```
100%|██████████████████████████████| 326/326 [00:00<00:00, 18267.29it/s]
```

Out[36]: **Table SBI: LMNA smooth muscle cells combined up/downregulated genes, standard method, FDR = 0.1.**

	title	pval (uncorrected)	# entities in list	#entities in model	shared entities in gocam	url
0	Eukaryotic Translation Elongation - Reactome	0.000000	16	94	['EEF1A2', 'RPL13A', 'RPLP2', 'RPS18', 'RPL34', 'RPLP0', 'RPL7', 'RPL3', 'RPS15A', 'RPS20', 'RPL23A', 'RPS15', 'RPL27A', 'UBA52', 'RPL35', 'RPS5']	http://model.geneontology.org/R-HSA-156842
1	Peptide chain elongation - Reactome	0.000000	15	88	['RPL13A', 'RPLP2', 'RPS18', 'RPL34', 'RPLP0', 'RPL7', 'RPL3', 'RPS15A', 'RPS20', 'RPL23A', 'RPS15', 'RPL27A', 'UBA52', 'RPL35', 'RPS5']	http://model.geneontology.org/R-HSA-156902

```
In [37]: LMNA_weighted = enrich.enrich_wrapper('LMNA_SMC_comb.csv','Gene Symbol',method='ncHGT',FDR = 0.1,fpath = '../test_data/processed/',table_number = 36)
LMNA_weighted
```

```
100%|██████████████████████████████| 326/326 [02:04<00:00,  2.63it/s]
Analysis run on 210 entities from 161 out of 633 input genes
```

Out[37]: **Table SBJ: LMNA smooth muscle cells combined up/downregulated genes, weighted set method, FDR = 0.1.**

	title	pval (uncorrected)	# entities in list	#entities in model	shared entities in gocam	url
0	Eukaryotic Translation Elongation - Reactome	0.000000	16	83	['RPL13A', 'RPLP2', 'RPS18', 'RPL34', 'RPLP0', 'RPL7', 'RPS15A', 'RPS20', 'RPL23A', 'RPS15', 'RPL27A', 'UBA52', 'RPL35', 'RPS5', 'set:EEF1A1-like proteins', 'set:RPL3,RPL3L']	http://model.geneontology.org/R-HSA-156842
1	Peptide chain elongation - Reactome	0.000000	15	79	['RPL13A', 'RPLP2', 'RPS18', 'RPL34', 'RPLP0', 'RPL7', 'RPS15A', 'RPS20', 'RPL23A', 'RPS15', 'RPL27A', 'UBA52', 'RPL35', 'RPS5', 'set:RPL3,RPL3L']	http://model.geneontology.org/R-HSA-156902

RBM20

```
In [38]: RBM20_set = enrich.enrich_wrapper('RBM20_SMC_comb.csv','Gene Symbol',method='set',FDR = 0.1,fpath = '../test_data/processed/',table_number = 37)
RBM20_set
```

```
100%|██████████████████████████████| 195/195 [00:00<00:00, 18175.32it/s]
Analysis run on 135 entities from 103 out of 435 input genes
```

Out[38]: **Table SBK: RBM20 smooth muscle cells combined up/downregulated genes, unweighted set method, FDR = 0.1.**

	title	pval (uncorrected)	# entities in list	#entities in model	shared entities in gocam	url
0	Regulation of Complement cascade - Reactome	0.000016	5	11	['C3', 'set:CD46, CR1:C4b:C3b complexes', 'set:C3 convertases', 'set:Complement factor D', 'set:C4 activators']	http://model.geneontology.org/R-HSA-977606

```
In [39]: RBM20_standard = enrich.enrich_wrapper('RBM20_SMC_comb.csv','Gene Symbol',method='standard',FDR = 0.1,fpath = '../test_data/processed/',table_number = 38)
RBM20_standard
```

```
100%|██████████████████████████████| 195/195 [00:00<00:00, 18052.16it/s]
```

Out[39]: **Table SBL: RBM20 smooth muscle cells combined up/downregulated genes, standard method, FDR = 0.1.**

	title	pval (uncorrected)	# entities in list	#entities in model	shared entities in gocam	url
0	Eukaryotic Translation Elongation - Reactome	0.000017	10	94	['EEF1A2', 'RPL13A', 'RPS18', 'RPLP0', 'RPS15A', 'RPLP2', 'RPL34', 'RPL27A', 'RPS20', 'RPL38']	http://model.geneontology.org/R-HSA-156842
1	Peptide chain elongation - Reactome	0.000062	9	88	['RPL13A', 'RPS18', 'RPLP0', 'RPS15A', 'RPLP2', 'RPL34', 'RPL27A', 'RPS20', 'RPL38']	http://model.geneontology.org/R-HSA-156902

```
In [40]: RBM20_weighted = enrich.enrich_wrapper('RBM20_SMC_comb.csv','Gene Symbol',method='ncHGT',FDR = 0.1,fpath = '../test_data/processed/',table_number = 39)
RBM20_weighted
```

```
100%|██████████████████████████████| 195/195 [01:29<00:00,  2.19it/s]
Analysis run on 135 entities from 103 out of 435 input genes
```

Out[40]: **Table SBM: RBM20 smooth muscle cells combined up/downregulated genes, weighted set method, FDR = 0.1.**

	title	pval (uncorrected)	# entities in list	#entities in model	shared entities in gocam	url
0	Eukaryotic Translation Elongation - Reactome	0.000004	10	83	['RPL13A', 'RPS18', 'RPLP0', 'RPS15A', 'RPLP2', 'RPL34', 'RPL27A', 'RPS20', 'RPL38', 'set:EEF1A1-like proteins']	http://model.geneontology.org/R-HSA-156842
1	Peptide chain elongation - Reactome	0.000019	9	79	['RPL13A', 'RPS18', 'RPLP0', 'RPS15A', 'RPLP2', 'RPL34', 'RPL27A', 'RPS20', 'RPL38']	http://model.geneontology.org/R-HSA-156902

PKP2

```
In [41]: PKP2_set = enrich.enrich_wrapper('PKP2_SMC_comb.csv','Gene Symbol',method='set',FDR = 0.1,fpath = '../test_data/processed/',table_number = 40)
PKP2_set
```

```
100%|██████████████████████████████| 185/185 [00:00<00:00, 18153.34it/s]
Analysis run on 92 entities from 65 out of 309 input genes
```

Out[41]: **Table SBN: PKP2 smooth muscle cells combined up/downregulated genes, unweighted set method, FDR = 0.1.**

title	pval (uncorrected)	# entities in list	#entities in model	shared entities in gocam	url

```
In [42]: PKP2_standard = enrich.enrich_wrapper('PKP2_SMC_comb.csv','Gene Symbol',method='standard',FDR = 0.1,fpath = '../test_data/processed/',table_number = 41)
PKP2_standard
```

```
100%|██████████████████████████████| 185/185 [00:00<00:00, 18027.65it/s]
```

Out[42]: **Table SBO: PKP2 smooth muscle cells combined up/downregulated genes, standard method, FDR = 0.1.**

title	pval (uncorrected)	# entities in list	#entities in model	shared entities in gocam	url

```
In [43]: PKP2_weighted = enrich.enrich_wrapper('PKP2_SMC_comb.csv','Gene Symbol',method='ncHGT',FDR = 0.1,fpath = '../test_data/processed/',table_number = 42)
PKP2_weighted
```

```
100%|██████████████████████████████| 185/185 [01:16<00:00,  2.40it/s]
Analysis run on 92 entities from 65 out of 309 input genes
```

Out[43]: **Table SBP: PKP2 smooth muscle cells combined up/downregulated genes, weighted set method, FDR = 0.1.**

title	pval (uncorrected)	# entities in list	#entities in model	shared entities in gocam	url

TTN

```
In [44]: TTN_set = enrich.enrich_wrapper('TTN_SMC_comb.csv','Gene Symbol',method='set',FDR = 0.1,fpath = '../test_data/processed/',table_number = 43)
TTN_set
```

```
100%|██████████████████████████████| 268/268 [00:00<00:00, 18542.95it/s]
Analysis run on 139 entities from 103 out of 396 input genes
```

Out[44]: **Table SBQ: TTN smooth muscle cells combined up/downregulated genes, unweighted set method, FDR = 0.1.**

	title	pval (uncorrected)	# entities in list	#entities in model	shared entities in gocam	url
0	Eukaryotic Translation Elongation - Reactome	0.000000	16	83	['RPL13A', 'RPS18', 'RPLP0', 'RPLP2', 'RPS5', 'RPL18', 'RPL34', 'RPL7', 'RPS14', 'RPL27', 'RPS15', 'RPS15A', 'RPS25', 'RPSA', 'set:EEF1A1-like proteins', 'set:RPL3,RPL3L']	http://model.geneontology.org/R-HSA-156842
1	Peptide chain elongation - Reactome	0.000000	15	79	['RPL13A', 'RPS18', 'RPLP0', 'RPLP2', 'RPS5', 'RPL18', 'RPL34', 'RPL7', 'RPS14', 'RPL27', 'RPS15', 'RPS15A', 'RPS25', 'RPSA', 'set:RPL3,RPL3L']	http://model.geneontology.org/R-HSA-156902
2	Regulation of Complement cascade - Reactome	0.000001	6	11	['C3', 'C1R', 'set:CD46, CR1:C4b:C3b complexes', 'set:C3 convertases', 'set:C4 activators', 'set:Complement factor D']	http://model.geneontology.org/R-HSA-977606

```
In [45]: TTN_standard = enrich.enrich_wrapper('TTN_SMC_comb.csv','Gene Symbol',method='standard',FDR = 0.1,fpath = '../test_data/processed/',table_number = 44)
TTN_standard
```

```
100%|██████████████████████████████| 268/268 [00:00<00:00, 18043.78it/s]
```

Out[45]: **Table SBR: TTN smooth muscle cells combined up/downregulated genes, standard method, FDR = 0.1.**

	title	pval (uncorrected)	# entities in list	#entities in model	shared entities in gocam	url
0	Eukaryotic Translation Elongation - Reactome	0.000000	16	94	['EEF1A2', 'RPL13A', 'RPS18', 'RPL3', 'RPLP0', 'RPLP2', 'RPS5', 'RPL18', 'RPL34', 'RPL7', 'RPS14', 'RPL27', 'RPS15', 'RPS15A', 'RPS25', 'RPSA']	http://model.geneontology.org/R-HSA-156842
1	Peptide chain elongation - Reactome	0.000000	15	88	['RPL13A', 'RPS18', 'RPL3', 'RPLP0', 'RPLP2', 'RPS5', 'RPL18', 'RPL34', 'RPL7', 'RPS14', 'RPL27', 'RPS15', 'RPS15A', 'RPS25', 'RPSA']	http://model.geneontology.org/R-HSA-156902

```
In [46]: TTN_weighted = enrich.enrich_wrapper('TTN_SMC_comb.csv','Gene Symbol',method='ncHGT',FDR = 0.1,fpath = '../test_data/processed/',table_number = 45)
TTN_weighted
```

```
100%|██████████████████████████████| 268/268 [01:38<00:00,  2.73it/s]
Analysis run on 139 entities from 103 out of 396 input genes
```

Out[46]: **Table SBS: TTN smooth muscle cells combined up/downregulated genes, weighted set method, FDR = 0.1.**

	title	pval (uncorrected)	# entities in list	#entities in model	shared entities in gocam	url
0	Eukaryotic Translation Elongation - Reactome	0.000000	16	83	['RPL13A', 'RPS18', 'RPLP0', 'RPLP2', 'RPS5', 'RPL18', 'RPL34', 'RPL7', 'RPS14', 'RPL27', 'RPS15', 'RPS15A', 'RPS25', 'RPSA', 'set:EEF1A1-like proteins', 'set:RPL3,RPL3L']	http://model.geneontology.org/R-HSA-156842
1	Peptide chain elongation - Reactome	0.000000	15	79	['RPL13A', 'RPS18', 'RPLP0', 'RPLP2', 'RPS5', 'RPL18', 'RPL34', 'RPL7', 'RPS14', 'RPL27', 'RPS15', 'RPS15A', 'RPS25', 'RPSA', 'set:RPL3,RPL3L']	http://model.geneontology.org/R-HSA-156902
2	Regulation of Complement cascade - Reactome	0.000082	6	11	['C3', 'C1R', 'set:CD46, CR1:C4b:C3b complexes', 'set:C3 convertases', 'set:C4 activators', 'set:Complement factor D']	http://model.geneontology.org/R-HSA-977606

PVneg

```
In [47]: PVneg_set = enrich.enrich_wrapper('PVneg_SMC_comb.csv','Gene Symbol',method='set',FDR = 0.1,fpath = '../test_data/processed/',table_number = 46)
PVneg_set
```

```
100%|██████████████████████████████| 324/324 [00:00<00:00, 17431.43it/s]
Analysis run on 256 entities from 221 out of 686 input genes
```

Out[47]: **Table SBT: PVneg smooth muscle cells combined up/downregulated genes, unweighted set method, FDR = 0.1.**

	title	pval (uncorrected)	# entities in list	#entities in model	shared entities in gocam	url
0	Peptide chain elongation - Reactome	0.000000	41	79	['RPL13A', 'RPLP2', 'RPLP0', 'RPS18', 'RPL35', 'RPL27A', 'RPS15A', 'RPL7', 'RPS5', 'RPS2', 'RPS20', 'RPLP1', 'RPSA', 'RPL10A', 'RPS25', 'RPL15', 'RPL27', 'RPL23A', 'RPL34', 'RPS16', 'RPS9', 'RPS14', 'RPL8', 'RPS15', 'RPL38', 'RPS29', 'RPL13', 'RPL4', 'RPS19', 'RPL12', 'RPL29', 'RPS28', 'RPS6', 'RPS23', 'RPL21', 'UBA52', 'RPL41', 'set:RPL10,RPL10L', 'set:RPS27,RPS27L', 'set:RPL3,RPL3L', 'set:RPL36A,RPL36AL']	http://model.geneontology.org/R-HSA-156902
1	Eukaryotic Translation Elongation - Reactome	0.000000	41	83	['RPL13A', 'RPLP2', 'RPLP0', 'RPS18', 'RPL35', 'RPL27A', 'RPS15A', 'RPL7', 'RPS5', 'RPS2', 'RPS20', 'RPLP1', 'RPSA', 'RPL10A', 'RPS25', 'RPL15', 'RPL27', 'RPL23A', 'RPL34', 'RPS16', 'RPS9', 'RPS14', 'RPL8', 'RPS15', 'RPL38', 'RPS29', 'RPL13', 'RPL4', 'RPS19', 'RPL12', 'RPL29', 'RPS28', 'RPS6', 'RPS23', 'RPL21', 'UBA52', 'RPL41', 'set:RPL10,RPL10L', 'set:RPS27,RPS27L', 'set:RPL3,RPL3L', 'set:RPL36A,RPL36AL']	http://model.geneontology.org/R-HSA-156842
2	Formation of ATP by chemiosmotic coupling - Reactome	0.000000	23	88	['MT-ND4', 'MT-ND2', 'CYC1', 'UQCRQ', 'COX5B', 'ATP5MC1', 'COX7C', 'NDUFA1', 'MT-CO2', 'MT-CO1', 'ATP5F1D', 'ATP5MC3', 'UQCRB', 'COX6B1', 'NDUFA4', 'ATP5ME', 'NDUFA6', 'NDUFB3', 'ATP5PD', 'ATP5F1E', 'COX4I1', 'NDUFB11', 'set:ANT homodimer']	http://model.geneontology.org/R-HSA-163210
3	Respiratory electron transport - Reactome	0.000003	19	91	['MT-ND4', 'MT-ND2', 'CYC1', 'UQCRQ', 'COX5B', 'IDH3B', 'ACADVL', 'COX7C', 'NDUFA1', 'MT-CO2', 'MT-CO1', 'UQCRB', 'COX6B1', 'NDUFA4', 'NDUFA6', 'IDH3G', 'NDUFB3', 'COX4I1', 'NDUFB11']	http://model.geneontology.org/R-HSA-611105
4	The fatty acid cycling model - Reactome	0.000006	16	70	['MT-ND4', 'MT-ND2', 'CYC1', 'UQCRQ', 'COX5B', 'COX7C', 'NDUFA1', 'MT-CO2', 'MT-CO1', 'UQCRB', 'COX6B1', 'NDUFA4', 'NDUFA6', 'NDUFB3', 'COX4I1', 'NDUFB11']	http://model.geneontology.org/R-HSA-167826
5	The proton buffering model - Reactome	0.000006	16	70	['MT-ND4', 'MT-ND2', 'CYC1', 'UQCRQ', 'COX5B', 'COX7C', 'NDUFA1', 'MT-CO2', 'MT-CO1', 'UQCRB', 'COX6B1', 'NDUFA4', 'NDUFA6', 'NDUFB3', 'COX4I1', 'NDUFB11']	http://model.geneontology.org/R-HSA-167827
6	Cytoprotection by HMOX1 - Reactome	0.000007	10	29	['CYC1', 'UQCRQ', 'COX5B', 'COX7C', 'MT-CO2', 'MT-CO1', 'UQCRB', 'COX6B1', 'NDUFA4', 'COX4I1']	http://model.geneontology.org/R-HSA-9707564
7	Transport of nucleosides and free purine and pyrimidine bases across the plasma membrane - Reactome	0.000010	10	30	['ATP5MC1', 'ATP5F1D', 'ATP5MC3', 'SLC25A4', 'ATP5ME', 'ATP5PD', 'ATP5F1E', 'set:SLC29A1-like proteins', 'set:ANT homodimer', 'set:SLC29A2-like proteins']	http://model.geneontology.org/R-HSA-83936
8	TP53 Regulates Metabolic Genes - Reactome	0.000021	12	46	['CYC1', 'UQCRQ', 'COX5B', 'COX7C', 'MT-CO2', 'MT-CO1', 'UQCRB', 'COX6B1', 'NDUFA4', 'COX4I1', 'PRDX1', 'set:PRDX1,2,5']	http://model.geneontology.org/R-HSA-5628897
9	Chondroitin sulfate biosynthesis - Reactome	0.000107	5	9	['set:CHPF,CHPF2,CHSY3', 'set:CHPF,CHSY3', 'set:B3GAT dimers', 'set:PAPST1,2', 'set:CHST9,11,12,13']	http://model.geneontology.org/R-HSA-2022870
10	Hydrolysis of LPC - Reactome	0.000500	4	7	['set:PLA2(5)', 'set:PLA2(7)', 'set:PLA2(6)', 'set:PLA2(8)']	http://model.geneontology.org/R-HSA-1483115

```
In [48]: PVneg_standard = enrich.enrich_wrapper('PVneg_SMC_comb.csv','Gene Symbol',method='standard',FDR = 0.1,fpath = '../test_data/processed/',table_number = 47)
PVneg_standard
```

```
100%|██████████████████████████████| 324/324 [00:00<00:00, 17802.04it/s]
```

Out[48]: **Table SBU: PVneg smooth muscle cells combined up/downregulated genes, standard method, FDR = 0.1.**

	title	pval (uncorrected)	# entities in list	#entities in model	shared entities in gocam	url
0	Peptide chain elongation - Reactome	0.000000	42	88	['RPL13A', 'RPLP2', 'RPLP0', 'RPS18', 'RPL35', 'RPL3', 'RPL27A', 'RPS15A', 'RPL7', 'RPS5', 'RPS2', 'RPS20', 'RPS27', 'RPLP1', 'RPL3L', 'RPSA', 'RPL10A', 'RPS25', 'RPL15', 'RPL27', 'RPL23A', 'RPL34', 'RPS16', 'RPS9', 'RPS14', 'RPL8', 'RPS15', 'RPL38', 'RPL10', 'RPL36AL', 'RPS29', 'RPL13', 'RPL4', 'RPS19', 'RPL12', 'RPL29', 'RPS28', 'RPS6', 'RPS23', 'RPL21', 'UBA52', 'RPL41']	http://model.geneontology.org/R-HSA-156902
1	Eukaryotic Translation Elongation - Reactome	0.000000	42	94	['RPL13A', 'RPLP2', 'RPLP0', 'RPS18', 'RPL35', 'RPL3', 'RPL27A', 'RPS15A', 'RPL7', 'RPS5', 'RPS2', 'RPS20', 'RPS27', 'RPLP1', 'RPL3L', 'RPSA', 'RPL10A', 'RPS25', 'RPL15', 'RPL27', 'RPL23A', 'RPL34', 'RPS16', 'RPS9', 'RPS14', 'RPL8', 'RPS15', 'RPL38', 'RPL10', 'RPL36AL', 'RPS29', 'RPL13', 'RPL4', 'RPS19', 'RPL12', 'RPL29', 'RPS28', 'RPS6', 'RPS23', 'RPL21', 'UBA52', 'RPL41']	http://model.geneontology.org/R-HSA-156842
2	Formation of ATP by chemiosmotic coupling - Reactome	0.000000	24	89	['MT-ND4', 'MT-ND2', 'SLC25A6', 'CYC1', 'UQCRQ', 'COX5B', 'ATP5MC1', 'SLC25A5', 'COX7C', 'NDUFA1', 'MT-CO2', 'MT-CO1', 'ATP5F1D', 'ATP5MC3', 'UQCRB', 'COX6B1', 'NDUFA4', 'ATP5ME', 'NDUFA6', 'NDUFB3', 'ATP5PD', 'ATP5F1E', 'COX4I1', 'NDUFB11']	http://model.geneontology.org/R-HSA-163210
3	Respiratory electron transport - Reactome	0.000000	19	91	['MT-ND4', 'MT-ND2', 'CYC1', 'UQCRQ', 'COX5B', 'IDH3B', 'ACADVL', 'COX7C', 'NDUFA1', 'MT-CO2', 'MT-CO1', 'UQCRB', 'COX6B1', 'NDUFA4', 'NDUFA6', 'IDH3G', 'NDUFB3', 'COX4I1', 'NDUFB11']	http://model.geneontology.org/R-HSA-611105
4	The fatty acid cycling model - Reactome	0.000000	16	74	['MT-ND4', 'MT-ND2', 'CYC1', 'UQCRQ', 'COX5B', 'COX7C', 'NDUFA1', 'MT-CO2', 'MT-CO1', 'UQCRB', 'COX6B1', 'NDUFA4', 'NDUFA6', 'NDUFB3', 'COX4I1', 'NDUFB11']	http://model.geneontology.org/R-HSA-167826
5	The proton buffering model - Reactome	0.000000	16	74	['MT-ND4', 'MT-ND2', 'CYC1', 'UQCRQ', 'COX5B', 'COX7C', 'NDUFA1', 'MT-CO2', 'MT-CO1', 'UQCRB', 'COX6B1', 'NDUFA4', 'NDUFA6', 'NDUFB3', 'COX4I1', 'NDUFB11']	http://model.geneontology.org/R-HSA-167827
6	Cytoprotection by HMOX1 - Reactome	0.000000	10	31	['CYC1', 'UQCRQ', 'COX5B', 'COX7C', 'MT-CO2', 'MT-CO1', 'UQCRB', 'COX6B1', 'NDUFA4', 'COX4I1']	http://model.geneontology.org/R-HSA-9707564
7	Transport of nucleosides and free purine and pyrimidine bases across the plasma membrane - Reactome	0.000000	10	32	['SLC25A6', 'ATP5MC1', 'SLC25A5', 'ATP5F1D', 'ATP5MC3', 'SLC25A4', 'ATP5ME', 'SLC29A1', 'ATP5PD', 'ATP5F1E']	http://model.geneontology.org/R-HSA-83936
8	TP53 Regulates Metabolic Genes - Reactome	0.000028	11	59	['CYC1', 'UQCRQ', 'COX5B', 'COX7C', 'MT-CO2', 'MT-CO1', 'UQCRB', 'COX6B1', 'NDUFA4', 'COX4I1', 'PRDX1']	http://model.geneontology.org/R-HSA-5628897
9	Canonical glycolysis 1 (Mouse)	0.000537	4	10	['TPI1', 'GAPDH', 'ENO3', 'PGAM2']	http://model.geneontology.org/5745387b00001516

```
In [49]: PVneg_weighted = enrich.enrich_wrapper('PVneg_SMC_comb.csv','Gene Symbol',method='ncHGT',FDR = 0.1,fpath = '../test_data/processed/',table_number = 48)
PVneg_weighted
```

```
100%|██████████████████████████████| 324/324 [00:38<00:00,  8.51it/s]
Analysis run on 256 entities from 221 out of 686 input genes
```

Out[49]: **Table SBV: PVneg smooth muscle cells combined up/downregulated genes, weighted set method, FDR = 0.1.**

	title	pval (uncorrected)	# entities in list	#entities in model	shared entities in gocam	url
0	Peptide chain elongation - Reactome	0.000000	41	79	['RPL13A', 'RPLP2', 'RPLP0', 'RPS18', 'RPL35', 'RPL27A', 'RPS15A', 'RPL7', 'RPS5', 'RPS2', 'RPS20', 'RPLP1', 'RPSA', 'RPL10A', 'RPS25', 'RPL15', 'RPL27', 'RPL23A', 'RPL34', 'RPS16', 'RPS9', 'RPS14', 'RPL8', 'RPS15', 'RPL38', 'RPS29', 'RPL13', 'RPL4', 'RPS19', 'RPL12', 'RPL29', 'RPS28', 'RPS6', 'RPS23', 'RPL21', 'UBA52', 'RPL41', 'set:RPL10,RPL10L', 'set:RPS27,RPS27L', 'set:RPL3,RPL3L', 'set:RPL36A,RPL36AL']	http://model.geneontology.org/R-HSA-156902
1	Eukaryotic Translation Elongation - Reactome	0.000000	41	83	['RPL13A', 'RPLP2', 'RPLP0', 'RPS18', 'RPL35', 'RPL27A', 'RPS15A', 'RPL7', 'RPS5', 'RPS2', 'RPS20', 'RPLP1', 'RPSA', 'RPL10A', 'RPS25', 'RPL15', 'RPL27', 'RPL23A', 'RPL34', 'RPS16', 'RPS9', 'RPS14', 'RPL8', 'RPS15', 'RPL38', 'RPS29', 'RPL13', 'RPL4', 'RPS19', 'RPL12', 'RPL29', 'RPS28', 'RPS6', 'RPS23', 'RPL21', 'UBA52', 'RPL41', 'set:RPL10,RPL10L', 'set:RPS27,RPS27L', 'set:RPL3,RPL3L', 'set:RPL36A,RPL36AL']	http://model.geneontology.org/R-HSA-156842
2	Formation of ATP by chemiosmotic coupling - Reactome	0.000000	23	88	['MT-ND4', 'MT-ND2', 'CYC1', 'UQCRQ', 'COX5B', 'ATP5MC1', 'COX7C', 'NDUFA1', 'MT-CO2', 'MT-CO1', 'ATP5F1D', 'ATP5MC3', 'UQCRB', 'COX6B1', 'NDUFA4', 'ATP5ME', 'NDUFA6', 'NDUFB3', 'ATP5PD', 'ATP5F1E', 'COX4I1', 'NDUFB11', 'set:ANT homodimer']	http://model.geneontology.org/R-HSA-163210
3	Respiratory electron transport - Reactome	0.000000	19	91	['MT-ND4', 'MT-ND2', 'CYC1', 'UQCRQ', 'COX5B', 'IDH3B', 'ACADVL', 'COX7C', 'NDUFA1', 'MT-CO2', 'MT-CO1', 'UQCRB', 'COX6B1', 'NDUFA4', 'NDUFA6', 'IDH3G', 'NDUFB3', 'COX4I1', 'NDUFB11']	http://model.geneontology.org/R-HSA-611105
4	The fatty acid cycling model - Reactome	0.000000	16	70	['MT-ND4', 'MT-ND2', 'CYC1', 'UQCRQ', 'COX5B', 'COX7C', 'NDUFA1', 'MT-CO2', 'MT-CO1', 'UQCRB', 'COX6B1', 'NDUFA4', 'NDUFA6', 'NDUFB3', 'COX4I1', 'NDUFB11']	http://model.geneontology.org/R-HSA-167826
5	The proton buffering model - Reactome	0.000000	16	70	['MT-ND4', 'MT-ND2', 'CYC1', 'UQCRQ', 'COX5B', 'COX7C', 'NDUFA1', 'MT-CO2', 'MT-CO1', 'UQCRB', 'COX6B1', 'NDUFA4', 'NDUFA6', 'NDUFB3', 'COX4I1', 'NDUFB11']	http://model.geneontology.org/R-HSA-167827
6	Cytoprotection by HMOX1 - Reactome	0.000000	10	29	['CYC1', 'UQCRQ', 'COX5B', 'COX7C', 'MT-CO2', 'MT-CO1', 'UQCRB', 'COX6B1', 'NDUFA4', 'COX4I1']	http://model.geneontology.org/R-HSA-9707564
7	Transport of nucleosides and free purine and pyrimidine bases across the plasma membrane - Reactome	0.000000	10	30	['ATP5MC1', 'ATP5F1D', 'ATP5MC3', 'SLC25A4', 'ATP5ME', 'ATP5PD', 'ATP5F1E', 'set:SLC29A1-like proteins', 'set:ANT homodimer', 'set:SLC29A2-like proteins']	http://model.geneontology.org/R-HSA-83936
8	TP53 Regulates Metabolic Genes - Reactome	0.000000	12	46	['CYC1', 'UQCRQ', 'COX5B', 'COX7C', 'MT-CO2', 'MT-CO1', 'UQCRB', 'COX6B1', 'NDUFA4', 'COX4I1', 'PRDX1', 'set:PRDX1,2,5']	http://model.geneontology.org/R-HSA-5628897
9	KEAP1-NFE2L2 pathway - Reactome	0.000228	9	55	['PSMC3', 'PSMC4', 'PSMB6', 'PSMC5', 'PSMB4', 'PSMA7', 'PRDX1', 'PSMB3', 'set:PRDX1,2,5']	http://model.geneontology.org/R-HSA-9755511
10	Conversion from APC/C:Cdc20 to APC/C:Cdh1 in late anaphase - Reactome	0.000480	8	49	['PSMC3', 'PSMC4', 'PSMB6', 'PSMC5', 'PSMB4', 'PSMA7', 'CDK2', 'PSMB3']	http://model.geneontology.org/R-HSA-176407
11	SCF(Skp2)-mediated degradation of p27/p21 - Reactome	0.000535	8	49	['PSMC3', 'PSMC4', 'PSMB6', 'PSMC5', 'PSMB4', 'PSMA7', 'CDK2', 'PSMB3']	http://model.geneontology.org/R-HSA-187577
12	Orc1 removal from chromatin - Reactome	0.000684	8	51	['PSMC3', 'PSMC4', 'PSMB6', 'PSMC5', 'PSMB4', 'PSMA7', 'CDK2', 'PSMB3']	http://model.geneontology.org/R-HSA-68949
13	Interleukin-1 signaling - Reactome	0.000818	9	64	['PSMC3', 'PSMC4', 'PSMB6', 'PSMC5', 'PSMB4', 'PSMA7', 'PSMB3', 'set:Pellino 1,2,3', 'set:p-Pellino-1,2,(3)']	http://model.geneontology.org/R-HSA-9020702
14	Regulation of APC/C activators between G1/S and early anaphase - Reactome	0.000840	8	52	['PSMC3', 'PSMC4', 'PSMB6', 'PSMC5', 'PSMB4', 'PSMA7', 'CDK2', 'PSMB3']	http://model.geneontology.org/R-HSA-176408
15	Cyclin A:Cdk2-associated events at S phase entry - Reactome	0.001120	8	52	['PSMC3', 'PSMC4', 'PSMB6', 'PSMC5', 'PSMB4', 'PSMA7', 'CDK2', 'PSMB3']	http://model.geneontology.org/R-HSA-69656
16	Chondroitin sulfate biosynthesis - Reactome	0.001234	5	9	['set:CHPF,CHPF2,CHSY3', 'set:CHPF,CHSY3', 'set:B3GAT dimers', 'set:PAPST1,2', 'set:CHST9,11,12,13']	http://model.geneontology.org/R-HSA-2022870
17	GSK3B and BTRC:CUL1-mediated-degradation of NFE2L2 - Reactome	0.001563	7	44	['PSMC3', 'PSMC4', 'PSMB6', 'PSMC5', 'PSMB4', 'PSMA7', 'PSMB3']	http://model.geneontology.org/R-HSA-9762114
18	AUF1 (hnRNP D0) binds and destabilizes mRNA - Reactome	0.001762	7	45	['PSMC3', 'PSMC4', 'PSMB6', 'PSMC5', 'PSMB4', 'PSMA7', 'PSMB3']	http://model.geneontology.org/R-HSA-450408
19	Cross-presentation of soluble exogenous antigens (endosomes) - Reactome	0.001762	7	45	['PSMC3', 'PSMC4', 'PSMB6', 'PSMC5', 'PSMB4', 'PSMA7', 'PSMB3']	http://model.geneontology.org/R-HSA-1236978
20	APC/C:Cdc20 mediated degradation of mitotic proteins - Reactome	0.001762	7	45	['PSMC3', 'PSMC4', 'PSMB6', 'PSMC5', 'PSMB4', 'PSMA7', 'PSMB3']	http://model.geneontology.org/R-HSA-176409
21	Regulation of RUNX3 expression and activity - Reactome	0.001947	7	45	['PSMC3', 'PSMC4', 'PSMB6', 'PSMC5', 'PSMB4', 'PSMA7', 'PSMB3']	http://model.geneontology.org/R-HSA-8941858
22	ABC-family proteins mediated transport - Reactome	0.001971	8	54	['PSMC3', 'PSMC4', 'PSMB6', 'PSMC5', 'PSMB4', 'PSMA7', 'PSMB3', 'set:RNF5,RNF185']	http://model.geneontology.org/R-HSA-382556
23	Ubiquitin-dependent degradation of Cyclin D - Reactome	0.001979	7	46	['PSMC3', 'PSMC4', 'PSMB6', 'PSMC5', 'PSMB4', 'PSMA7', 'PSMB3']	http://model.geneontology.org/R-HSA-75815
24	Regulation of activated PAK-2p34 by proteasome mediated degradation - Reactome	0.001979	7	46	['PSMC3', 'PSMC4', 'PSMB6', 'PSMC5', 'PSMB4', 'PSMA7', 'PSMB3']	http://model.geneontology.org/R-HSA-211733
25	Regulation of PTEN stability and activity - Reactome	0.002128	8	56	['FRK', 'PSMC3', 'PSMC4', 'PSMB6', 'PSMC5', 'PSMB4', 'PSMA7', 'PSMB3']	http://model.geneontology.org/R-HSA-8948751
26	Asymmetric localization of PCP proteins - Reactome	0.002182	7	46	['PSMC3', 'PSMC4', 'PSMB6', 'PSMC5', 'PSMB4', 'PSMA7', 'PSMB3']	http://model.geneontology.org/R-HSA-4608870
27	Autodegradation of the E3 ubiquitin ligase COP1 - Reactome	0.002216	7	47	['PSMC3', 'PSMC4', 'PSMB6', 'PSMC5', 'PSMB4', 'PSMA7', 'PSMB3']	http://model.geneontology.org/R-HSA-349425
28	G2/M Checkpoints - Reactome	0.002216	7	48	['PSMC3', 'PSMC4', 'PSMB6', 'PSMC5', 'PSMB4', 'PSMA7', 'PSMB3']	http://model.geneontology.org/R-HSA-69481
29	Degradation of DVL - Reactome	0.002216	7	47	['PSMC3', 'PSMC4', 'PSMB6', 'PSMC5', 'PSMB4', 'PSMA7', 'PSMB3']	http://model.geneontology.org/R-HSA-4641258
30	Canonical glycolysis 1 (Mouse)	0.002456	4	10	['TPI1', 'GAPDH', 'ENO3', 'PGAM2']	http://model.geneontology.org/5745387b00001516
31	TNFR2 non-canonical NF-kB pathway - Reactome	0.002676	7	47	['PSMC3', 'PSMC4', 'PSMB6', 'PSMC5', 'PSMB4', 'PSMA7', 'PSMB3']	http://model.geneontology.org/R-HSA-5668541
32	Degradation of AXIN - Reactome	0.002715	7	48	['PSMC3', 'PSMC4', 'PSMB6', 'PSMC5', 'PSMB4', 'PSMA7', 'PSMB3']	http://model.geneontology.org/R-HSA-4641257
33	The role of GTSE1 in G2/M progression after G2 checkpoint - Reactome	0.002754	7	50	['PSMC3', 'PSMC4', 'PSMB6', 'PSMC5', 'PSMB4', 'PSMA7', 'PSMB3']	http://model.geneontology.org/R-HSA-8852276
34	Ubiquitin Mediated Degradation of Phosphorylated Cdc25A - Reactome	0.002754	7	49	['PSMC3', 'PSMC4', 'PSMB6', 'PSMC5', 'PSMB4', 'PSMA7', 'PSMB3']	http://model.geneontology.org/R-HSA-69601
35	RUNX1 regulates transcription of genes involved in differentiation of HSCs - Reactome	0.002754	7	49	['PSMC3', 'PSMC4', 'PSMB6', 'PSMC5', 'PSMB4', 'PSMA7', 'PSMB3']	http://model.geneontology.org/R-HSA-8939236
36	Oxygen-dependent proline hydroxylation of Hypoxia-inducible Factor Alpha - Reactome	0.002974	7	48	['PSMC3', 'PSMC4', 'PSMB6', 'PSMC5', 'PSMB4', 'PSMA7', 'PSMB3']	http://model.geneontology.org/R-HSA-1234176
37	Hedgehog 'on' state - Reactome	0.003015	7	49	['PSMC3', 'PSMC4', 'PSMB6', 'PSMC5', 'PSMB4', 'PSMA7', 'PSMB3']	http://model.geneontology.org/R-HSA-5632684
38	FBXL7 down-regulates AURKA during mitotic entry and in early mitosis - Reactome	0.003058	7	50	['PSMC3', 'PSMC4', 'PSMB6', 'PSMC5', 'PSMB4', 'PSMA7', 'PSMB3']	http://model.geneontology.org/R-HSA-8854050
39	CDK-mediated phosphorylation and removal of Cdc6 - Reactome	0.003264	8	65	['PSMC3', 'PSMC4', 'PSMB6', 'PSMC5', 'PSMB4', 'PSMA7', 'CDK2', 'PSMB3']	http://model.geneontology.org/R-HSA-69017
40	Negative regulation of NOTCH4 signaling - Reactome	0.003296	7	49	['PSMC3', 'PSMC4', 'PSMB6', 'PSMC5', 'PSMB4', 'PSMA7', 'PSMB3']	http://model.geneontology.org/R-HSA-9604323
41	NIK-->noncanonical NF-kB signaling - Reactome	0.003340	7	50	['PSMC3', 'PSMC4', 'PSMB6', 'PSMC5', 'PSMB4', 'PSMA7', 'PSMB3']	http://model.geneontology.org/R-HSA-5676590
42	Dectin-1 mediated noncanonical NF-kB signaling - Reactome	0.003340	7	50	['PSMC3', 'PSMC4', 'PSMB6', 'PSMC5', 'PSMB4', 'PSMA7', 'PSMB3']	http://model.geneontology.org/R-HSA-5607761
43	SCF-beta-TrCP mediated degradation of Emi1 - Reactome	0.003385	7	51	['PSMC3', 'PSMC4', 'PSMB6', 'PSMC5', 'PSMB4', 'PSMA7', 'PSMB3']	http://model.geneontology.org/R-HSA-174113
44	Regulation of expression of SLITs and ROBOs - Reactome	0.003385	7	51	['PSMC3', 'PSMC4', 'PSMB6', 'PSMC5', 'PSMB4', 'PSMA7', 'PSMB3']	http://model.geneontology.org/R-HSA-9010553
45	Hedgehog 'off' state - Reactome	0.003469	7	46	['PSMC3', 'PSMC4', 'PSMB6', 'PSMC5', 'PSMB4', 'PSMA7', 'PSMB3']	http://model.geneontology.org/R-HSA-5610787
46	Hedgehog ligand biogenesis - Reactome	0.003607	7	50	['PSMC3', 'PSMC4', 'PSMB6', 'PSMC5', 'PSMB4', 'PSMA7', 'PSMB3']	http://model.geneontology.org/R-HSA-5358346
47	ER-Phagosome pathway - Reactome	0.003690	7	51	['PSMC3', 'PSMC4', 'PSMB6', 'PSMC5', 'PSMB4', 'PSMA7', 'PSMB3']	http://model.geneontology.org/R-HSA-1236974
48	Degradation of GLI1 by the proteasome - Reactome	0.003979	7	51	['PSMC3', 'PSMC4', 'PSMB6', 'PSMC5', 'PSMB4', 'PSMA7', 'PSMB3']	http://model.geneontology.org/R-HSA-5610780

Platelets in SARS-CoV-2

Kanth manne et al., 2020

https://ashpublications.org/blood/article/136/11/1317/461106/Platelet-gene-expression-and-function-in-patients

RNAseq in platelets from patients with covid-19 vs healthy donors. There were 6 ICU and 4 nonICU patients.

```
In [50]: platelets_up_set = enrich.enrich_wrapper('platelets_up.csv','Gene Symbol',method='set',FDR = 0.1,fpath = '../test_data/processed/',table_number = 49)
platelets_up_set
```

```
100%|██████████████████████████████| 482/482 [00:00<00:00, 17448.51it/s]
Analysis run on 423 entities from 365 out of 1172 input genes
```

Out[50]: **Table SBW: platelets up, unweighted set method, FDR = 0.1.**

	title	pval (uncorrected)	# entities in list	#entities in model	shared entities in gocam	url
0	Collagen biosynthesis and modifying enzymes - Reactome	0.000000	10	12	['PPIB', 'P3H1', 'PLOD3', 'P4HB', 'set:Procollagen C-proteinases', 'set:Prolyl 3-hydroxylases', 'set:COLGALT1,COLGALT2', 'set:4-Hyp collagen propeptides', 'set:Procollagen N-proteinases', 'set:Lysyl hydroxylases']	http://model.geneontology.org/R-HSA-1650814
1	Synthesis of PE - Reactome	0.000050	8	15	['PTDSS2', 'PCYT2', 'PISD', 'PHOSPHO1', 'set:CHK/ETNK', 'set:LPIN', 'set:AGPAT', 'set:PNPLA2/3']	http://model.geneontology.org/R-HSA-1483213

```
In [51]: platelets_up_standard = enrich.enrich_wrapper('platelets_up.csv','Gene Symbol',method='standard',FDR = 0.1,fpath = '../test_data/processed/',table_number = 50)
platelets_up_standard
```

```
100%|██████████████████████████████| 482/482 [00:00<00:00, 18241.04it/s]
```

Out[51]: **Table SBX: platelets up, standard method, FDR = 0.1.**

	title	pval (uncorrected)	# entities in list	#entities in model	shared entities in gocam	url
0	ER-Phagosome pathway - Reactome	0.000045	13	52	['SEC61A1', 'PSMD13', 'PSMA5', 'PSMD11', 'SEC61B', 'PSMA7', 'PSME2', 'PSMD4', 'PSMB1', 'PSMB7', 'PSMD8', 'PSMB5', 'PSMB6']	http://model.geneontology.org/R-HSA-1236974
1	Hedgehog ligand biogenesis - Reactome	0.000045	13	52	['PSMD13', 'PSMA5', 'PSMD11', 'PSMA7', 'PSME2', 'PSMD4', 'PSMB1', 'PSMB7', 'PSMD8', 'PSMB5', 'P4HB', 'PSMB6', 'SYVN1']	http://model.geneontology.org/R-HSA-5358346
2	Regulation of APC/C activators between G1/S and early anaphase - Reactome	0.000069	13	54	['PSMD13', 'PSMA5', 'PSMD11', 'PSMA7', 'PSME2', 'PSMD4', 'PSMB1', 'CDC25B', 'PSMB7', 'CDK1', 'PSMD8', 'PSMB5', 'PSMB6']	http://model.geneontology.org/R-HSA-176408
3	Conversion from APC/C:Cdc20 to APC/C:Cdh1 in late anaphase - Reactome	0.000111	12	49	['PSMD13', 'PSMA5', 'PSMD11', 'PSMA7', 'PSME2', 'PSMD4', 'PSMB1', 'PSMB7', 'CDK1', 'PSMD8', 'PSMB5', 'PSMB6']	http://model.geneontology.org/R-HSA-176407
4	Neddylation - Reactome	0.000113	14	64	['UBE2M', 'PSMD13', 'PSMA5', 'PSMD11', 'PSMA7', 'PSME2', 'PSMD4', 'PSMB1', 'CUL9', 'PSMB7', 'UCHL3', 'PSMD8', 'PSMB5', 'PSMB6']	http://model.geneontology.org/R-HSA-8951664
5	KEAP1-NFE2L2 pathway - Reactome	0.000126	13	57	['PSMD13', 'PSMA5', 'PSMD11', 'PSMA7', 'PSME2', 'PSMD4', 'PSMB1', 'PSMB7', 'CSNK2B', 'PSMD8', 'PSMB5', 'PSMB6', 'PRDX2']	http://model.geneontology.org/R-HSA-9755511
6	SCF(Skp2)-mediated degradation of p27/p21 - Reactome	0.000136	12	50	['PSMD13', 'PSMA5', 'PSMD11', 'PSMA7', 'PSME2', 'PSMD4', 'PSMB1', 'PSMB7', 'PSMD8', 'PSMB5', 'PSMB6', 'CKS1B']	http://model.geneontology.org/R-HSA-187577
7	The role of GTSE1 in G2/M progression after G2 checkpoint - Reactome	0.000136	12	50	['PSMD13', 'PSMA5', 'PSMD11', 'PSMA7', 'PSME2', 'PSMD4', 'PSMB1', 'CDC25B', 'PSMB7', 'PSMD8', 'PSMB5', 'PSMB6']	http://model.geneontology.org/R-HSA-8852276
8	SCF-beta-TrCP mediated degradation of Emi1 - Reactome	0.000167	12	51	['PSMD13', 'PSMA5', 'PSMD11', 'PSMA7', 'PSME2', 'PSMD4', 'PSMB1', 'PSMB7', 'CDK1', 'PSMD8', 'PSMB5', 'PSMB6']	http://model.geneontology.org/R-HSA-174113
9	GSK3B and BTRC:CUL1-mediated-degradation of NFE2L2 - Reactome	0.000175	11	44	['PSMD13', 'PSMA5', 'PSMD11', 'PSMA7', 'PSME2', 'PSMD4', 'PSMB1', 'PSMB7', 'PSMD8', 'PSMB5', 'PSMB6']	http://model.geneontology.org/R-HSA-9762114
10	Degradation of beta-catenin by the destruction complex - Reactome	0.000183	13	59	['PSMD13', 'PSMA5', 'PSMD11', 'PPP2R5B', 'PSMA7', 'PSME2', 'PSMD4', 'PSMB1', 'PSMB7', 'PPP2R1B', 'PSMD8', 'PSMB5', 'PSMB6']	http://model.geneontology.org/R-HSA-195253
11	Cross-presentation of soluble exogenous antigens (endosomes) - Reactome	0.000217	11	45	['PSMD13', 'PSMA5', 'PSMD11', 'PSMA7', 'PSME2', 'PSMD4', 'PSMB1', 'PSMB7', 'PSMD8', 'PSMB5', 'PSMB6']	http://model.geneontology.org/R-HSA-1236978
12	AUF1 (hnRNP D0) binds and destabilizes mRNA - Reactome	0.000217	11	45	['PSMD13', 'PSMA5', 'PSMD11', 'PSMA7', 'PSME2', 'PSMD4', 'PSMB1', 'PSMB7', 'PSMD8', 'PSMB5', 'PSMB6']	http://model.geneontology.org/R-HSA-450408
13	APC/C:Cdc20 mediated degradation of mitotic proteins - Reactome	0.000217	11	45	['PSMD13', 'PSMA5', 'PSMD11', 'PSMA7', 'PSME2', 'PSMD4', 'PSMB1', 'PSMB7', 'PSMD8', 'PSMB5', 'PSMB6']	http://model.geneontology.org/R-HSA-176409
14	Regulation of activated PAK-2p34 by proteasome mediated degradation - Reactome	0.000268	11	46	['PSMD13', 'PSMA5', 'PSMD11', 'PSMA7', 'PSME2', 'PSMD4', 'PSMB1', 'PSMB7', 'PSMD8', 'PSMB5', 'PSMB6']	http://model.geneontology.org/R-HSA-211733
15	Ubiquitin-dependent degradation of Cyclin D - Reactome	0.000268	11	46	['PSMD13', 'PSMA5', 'PSMD11', 'PSMA7', 'PSME2', 'PSMD4', 'PSMB1', 'PSMB7', 'PSMD8', 'PSMB5', 'PSMB6']	http://model.geneontology.org/R-HSA-75815
16	Regulation of RUNX3 expression and activity - Reactome	0.000268	11	46	['PSMD13', 'PSMA5', 'PSMD11', 'PSMA7', 'PSME2', 'PSMD4', 'PSMB1', 'PSMB7', 'PSMD8', 'PSMB5', 'PSMB6']	http://model.geneontology.org/R-HSA-8941858
17	Antigen Presentation: Folding, assembly and peptide loading of class I MHC - Reactome	0.000297	12	54	['PSMD13', 'PSMA5', 'PSMD11', 'PSMA7', 'PSME2', 'PSMD4', 'PSMB1', 'PSMB7', 'PSMD8', 'PSMB5', 'PSMB6', 'ERAP2']	http://model.geneontology.org/R-HSA-983170
18	Degradation of DVL - Reactome	0.000328	11	47	['PSMD13', 'PSMA5', 'PSMD11', 'PSMA7', 'PSME2', 'PSMD4', 'PSMB1', 'PSMB7', 'PSMD8', 'PSMB5', 'PSMB6']	http://model.geneontology.org/R-HSA-4641258
19	Autodegradation of the E3 ubiquitin ligase COP1 - Reactome	0.000328	11	47	['PSMD13', 'PSMA5', 'PSMD11', 'PSMA7', 'PSME2', 'PSMD4', 'PSMB1', 'PSMB7', 'PSMD8', 'PSMB5', 'PSMB6']	http://model.geneontology.org/R-HSA-349425
20	Asymmetric localization of PCP proteins - Reactome	0.000328	11	47	['PSMD13', 'PSMA5', 'PSMD11', 'PSMA7', 'PSME2', 'PSMD4', 'PSMB1', 'PSMB7', 'PSMD8', 'PSMB5', 'PSMB6']	http://model.geneontology.org/R-HSA-4608870
21	TNFR2 non-canonical NF-kB pathway - Reactome	0.000328	11	47	['PSMD13', 'PSMA5', 'PSMD11', 'PSMA7', 'PSME2', 'PSMD4', 'PSMB1', 'PSMB7', 'PSMD8', 'PSMB5', 'PSMB6']	http://model.geneontology.org/R-HSA-5668541
22	Synthesis of PE - Reactome	0.000338	9	33	['PTDSS2', 'LPIN1', 'PCYT2', 'PNPLA2', 'PISD', 'AGPAT2', 'CHKB', 'AGPAT4', 'PHOSPHO1']	http://model.geneontology.org/R-HSA-1483213
23	Heme biosynthesis - Reactome	0.000348	6	15	['ALAS2', 'UROS', 'COX10', 'HMBS', 'CPOX', 'PPOX']	http://model.geneontology.org/R-HSA-189451
24	G2/M Checkpoints - Reactome	0.000399	11	48	['PSMD13', 'PSMA5', 'PSMD11', 'PSMA7', 'PSME2', 'PSMD4', 'PSMB1', 'PSMB7', 'PSMD8', 'PSMB5', 'PSMB6']	http://model.geneontology.org/R-HSA-69481
25	Degradation of AXIN - Reactome	0.000483	11	49	['PSMD13', 'PSMA5', 'PSMD11', 'PSMA7', 'PSME2', 'PSMD4', 'PSMB1', 'PSMB7', 'PSMD8', 'PSMB5', 'PSMB6']	http://model.geneontology.org/R-HSA-4641257
26	Oxygen-dependent proline hydroxylation of Hypoxia-inducible Factor Alpha - Reactome	0.000483	11	49	['PSMD13', 'PSMA5', 'PSMD11', 'PSMA7', 'PSME2', 'PSMD4', 'PSMB1', 'PSMB7', 'PSMD8', 'PSMB5', 'PSMB6']	http://model.geneontology.org/R-HSA-1234176
27	RUNX1 regulates transcription of genes involved in differentiation of HSCs - Reactome	0.000483	11	49	['PSMD13', 'PSMA5', 'PSMD11', 'PSMA7', 'PSME2', 'PSMD4', 'PSMB1', 'PSMB7', 'PSMD8', 'PSMB5', 'PSMB6']	http://model.geneontology.org/R-HSA-8939236
28	Ubiquitin Mediated Degradation of Phosphorylated Cdc25A - Reactome	0.000483	11	49	['PSMD13', 'PSMA5', 'PSMD11', 'PSMA7', 'PSME2', 'PSMD4', 'PSMB1', 'PSMB7', 'PSMD8', 'PSMB5', 'PSMB6']	http://model.geneontology.org/R-HSA-69601
29	Cyclin A:Cdk2-associated events at S phase entry - Reactome	0.000505	12	57	['PSMD13', 'PSMA5', 'PSMD11', 'PSMA7', 'PSME2', 'PSMD4', 'PSMB1', 'CDC25B', 'PSMB7', 'PSMD8', 'PSMB5', 'PSMB6']	http://model.geneontology.org/R-HSA-69656
30	Respiratory electron transport - Reactome	0.000546	16	91	['NDUFS8', 'ACADVL', 'COX5B', 'NDUFS5', 'NDUFA8', 'NDUFB7', 'ETFB', 'NDUFB8', 'NDUFV2', 'COX5A', 'COX7C', 'NDUFV1', 'NDUFA11', 'NDUFA13', 'ACADS', 'UQCRC1']	http://model.geneontology.org/R-HSA-611105
31	FBXL7 down-regulates AURKA during mitotic entry and in early mitosis - Reactome	0.000580	11	50	['PSMD13', 'PSMA5', 'PSMD11', 'PSMA7', 'PSME2', 'PSMD4', 'PSMB1', 'PSMB7', 'PSMD8', 'PSMB5', 'PSMB6']	http://model.geneontology.org/R-HSA-8854050
32	Hedgehog 'on' state - Reactome	0.000580	11	50	['PSMD13', 'PSMA5', 'PSMD11', 'PSMA7', 'PSME2', 'PSMD4', 'PSMB1', 'PSMB7', 'PSMD8', 'PSMB5', 'PSMB6']	http://model.geneontology.org/R-HSA-5632684
33	NIK-->noncanonical NF-kB signaling - Reactome	0.000694	11	51	['PSMD13', 'PSMA5', 'PSMD11', 'PSMA7', 'PSME2', 'PSMD4', 'PSMB1', 'PSMB7', 'PSMD8', 'PSMB5', 'PSMB6']	http://model.geneontology.org/R-HSA-5676590
34	Negative regulation of NOTCH4 signaling - Reactome	0.000694	11	51	['PSMD13', 'PSMA5', 'PSMD11', 'PSMA7', 'PSME2', 'PSMD4', 'PSMB1', 'PSMB7', 'PSMD8', 'PSMB5', 'PSMB6']	http://model.geneontology.org/R-HSA-9604323
35	Regulation of expression of SLITs and ROBOs - Reactome	0.000694	11	51	['PSMD13', 'PSMA5', 'PSMD11', 'PSMA7', 'PSME2', 'PSMD4', 'PSMB1', 'PSMB7', 'PSMD8', 'PSMB5', 'PSMB6']	http://model.geneontology.org/R-HSA-9010553
36	Dectin-1 mediated noncanonical NF-kB signaling - Reactome	0.000694	11	51	['PSMD13', 'PSMA5', 'PSMD11', 'PSMA7', 'PSME2', 'PSMD4', 'PSMB1', 'PSMB7', 'PSMD8', 'PSMB5', 'PSMB6']	http://model.geneontology.org/R-HSA-5607761
37	Cyclin E associated events during G1/S transition - Reactome	0.000703	12	59	['PSMD13', 'PSMA5', 'PSMD11', 'CDK4', 'PSMA7', 'PSME2', 'PSMD4', 'PSMB1', 'PSMB7', 'PSMD8', 'PSMB5', 'PSMB6']	http://model.geneontology.org/R-HSA-69202
38	Orc1 removal from chromatin - Reactome	0.000825	11	52	['PSMD13', 'PSMA5', 'PSMD11', 'PSMA7', 'PSME2', 'PSMD4', 'PSMB1', 'PSMB7', 'PSMD8', 'PSMB5', 'PSMB6']	http://model.geneontology.org/R-HSA-68949
39	Degradation of GLI1 by the proteasome - Reactome	0.000977	11	53	['PSMD13', 'PSMA5', 'PSMD11', 'PSMA7', 'PSME2', 'PSMD4', 'PSMB1', 'PSMB7', 'PSMD8', 'PSMB5', 'PSMB6']	http://model.geneontology.org/R-HSA-5610780
40	GLI3 is processed to GLI3R by the proteasome - Reactome	0.001151	11	54	['PSMD13', 'PSMA5', 'PSMD11', 'PSMA7', 'PSME2', 'PSMD4', 'PSMB1', 'PSMB7', 'PSMD8', 'PSMB5', 'PSMB6']	http://model.geneontology.org/R-HSA-5610785
41	Regulation of RUNX2 expression and activity - Reactome	0.001151	11	54	['PSMD13', 'PSMA5', 'PSMD11', 'PSMA7', 'PSME2', 'PSMD4', 'PSMB1', 'PSMB7', 'PSMD8', 'PSMB5', 'PSMB6']	http://model.geneontology.org/R-HSA-8939902
42	Degradation of GLI2 by the proteasome - Reactome	0.001151	11	54	['PSMD13', 'PSMA5', 'PSMD11', 'PSMA7', 'PSME2', 'PSMD4', 'PSMB1', 'PSMB7', 'PSMD8', 'PSMB5', 'PSMB6']	http://model.geneontology.org/R-HSA-5610783
43	Hedgehog 'off' state - Reactome	0.001349	11	55	['PSMD13', 'PSMA5', 'PSMD11', 'PSMA7', 'PSME2', 'PSMD4', 'PSMB1', 'PSMB7', 'PSMD8', 'PSMB5', 'PSMB6']	http://model.geneontology.org/R-HSA-5610787
44	Regulation of PTEN stability and activity - Reactome	0.001495	12	64	['PSMD13', 'PSMA5', 'PSMD11', 'PSMA7', 'PSME2', 'PSMD4', 'PSMB1', 'PSMB7', 'CSNK2B', 'PSMD8', 'PSMB5', 'PSMB6']	http://model.geneontology.org/R-HSA-8948751
45	CLEC7A (Dectin-1) signaling - Reactome	0.001575	11	56	['PSMD13', 'PSMA5', 'PSMD11', 'PSMA7', 'PSME2', 'PSMD4', 'PSMB1', 'PSMB7', 'PSMD8', 'PSMB5', 'PSMB6']	http://model.geneontology.org/R-HSA-5607764
46	FCERI mediated NF-kB activation - Reactome	0.001575	11	56	['PSMD13', 'PSMA5', 'PSMD11', 'PSMA7', 'PSME2', 'PSMD4', 'PSMB1', 'PSMB7', 'PSMD8', 'PSMB5', 'PSMB6']	http://model.geneontology.org/R-HSA-2871837
47	The proton buffering model - Reactome	0.001795	13	74	['NDUFS8', 'COX5B', 'NDUFS5', 'NDUFA8', 'NDUFB7', 'NDUFB8', 'NDUFV2', 'COX5A', 'COX7C', 'NDUFV1', 'NDUFA11', 'NDUFA13', 'UQCRC1']	http://model.geneontology.org/R-HSA-167827
48	The fatty acid cycling model - Reactome	0.001795	13	74	['NDUFS8', 'COX5B', 'NDUFS5', 'NDUFA8', 'NDUFB7', 'NDUFB8', 'NDUFV2', 'COX5A', 'COX7C', 'NDUFV1', 'NDUFA11', 'NDUFA13', 'UQCRC1']	http://model.geneontology.org/R-HSA-167826
49	Activation of NF-kappaB in B cells - Reactome	0.002120	11	58	['PSMD13', 'PSMA5', 'PSMD11', 'PSMA7', 'PSME2', 'PSMD4', 'PSMB1', 'PSMB7', 'PSMD8', 'PSMB5', 'PSMB6']	http://model.geneontology.org/R-HSA-1169091
50	HS-GAG degradation - Reactome	0.002333	4	9	['HPSE', 'IDUA', 'NAGLU', 'GUSB']	http://model.geneontology.org/R-HSA-2024096
51	Autodegradation of Cdh1 by Cdh1:APC/C - Reactome	0.002444	11	59	['PSMD13', 'PSMA5', 'PSMD11', 'PSMA7', 'PSME2', 'PSMD4', 'PSMB1', 'PSMB7', 'PSMD8', 'PSMB5', 'PSMB6']	http://model.geneontology.org/R-HSA-174084
52	MAPK6/MAPK4 signaling - Reactome	0.002564	12	68	['PSMD13', 'PSMA5', 'PSMD11', 'PSMA7', 'PSME2', 'PSMD4', 'PSMB1', 'PSMB7', 'CDK1', 'PSMD8', 'PSMB5', 'PSMB6']	http://model.geneontology.org/R-HSA-5687128
53	Assembly of the pre-replicative complex - Reactome	0.002808	11	60	['PSMD13', 'PSMA5', 'PSMD11', 'PSMA7', 'PSME2', 'PSMD4', 'PSMB1', 'PSMB7', 'PSMD8', 'PSMB5', 'PSMB6']	http://model.geneontology.org/R-HSA-68867
54	Formation of ATP by chemiosmotic coupling - Reactome	0.003556	14	89	['ATP5F1D', 'NDUFS8', 'COX5B', 'NDUFS5', 'NDUFA8', 'NDUFB7', 'NDUFB8', 'NDUFV2', 'COX5A', 'COX7C', 'NDUFV1', 'NDUFA11', 'NDUFA13', 'UQCRC1']	http://model.geneontology.org/R-HSA-163210
[illegible]	APC/C:Cdh1 mediated degradation of Cdc20 and other [illegible]	[illegible]	[illegible]	[illegible]	[illegible]	[illegible]

55	APC/C:Cdh1 mediated degradation of Cdc20 and other APC/C:Cdh1 targeted proteins in late mitosis/early G1 - Reactome	0.003666	11	62	['PSMD13', 'PSMA5', 'PSMD11', 'PSMA7', 'PSME2', 'PSMD4', 'PSMB1', 'PSMB7', 'PSMD8', 'PSMB5', 'PSMB6']	http://model.geneontology.org/R-HSA-174178
56	APC/C:Cdc20 mediated degradation of Securin - Reactome	0.003666	11	62	['PSMD13', 'PSMA5', 'PSMD11', 'PSMA7', 'PSME2', 'PSMD4', 'PSMB1', 'PSMB7', 'PSMD8', 'PSMB5', 'PSMB6']	http://model.geneontology.org/R-HSA-174154
57	Cdc20:Phospho-APC/C mediated degradation of Cyclin A - Reactome	0.003666	11	62	['PSMD13', 'PSMA5', 'PSMD11', 'PSMA7', 'PSME2', 'PSMD4', 'PSMB1', 'PSMB7', 'PSMD8', 'PSMB5', 'PSMB6']	http://model.geneontology.org/R-HSA-174184
58	Heparan sulfate proteoglycan catabolic process (Mouse)	0.003674	4	10	['HPSE', 'IDUA', 'NAGLU', 'GUSB']	http://model.geneontology.org/627d95ee00000275
59	Regulation of RAS by GAPs - Reactome	0.004167	11	63	['PSMD13', 'PSMA5', 'PSMD11', 'PSMA7', 'PSME2', 'PSMD4', 'PSMB1', 'PSMB7', 'PSMD8', 'PSMB5', 'PSMB6']	http://model.geneontology.org/R-HSA-5658442
60	Separation of Sister Chromatids - Reactome	0.004167	11	63	['PSMD13', 'PSMA5', 'PSMD11', 'PSMA7', 'PSME2', 'PSMD4', 'PSMB1', 'PSMB7', 'PSMD8', 'PSMB5', 'PSMB6']	http://model.geneontology.org/R-HSA-2467813
61	ABC-family proteins mediated transport - Reactome	0.004722	11	64	['PSMD13', 'PSMA5', 'PSMD11', 'PSMA7', 'PSME2', 'PSMD4', 'PSMB1', 'PSMB7', 'PSMD8', 'PSMB5', 'PSMB6']	http://model.geneontology.org/R-HSA-382556

In [52]:
```
platelets_up_weighted = enrich.enrich_wrapper('platelets_up.csv','Gene Symbol',method='ncHGT',FDR = 0.1,fpath = '../test_data/processed/',table_number = 51)
platelets_up_weighted
```

```
100%|████████████████████████████████████████| 482/482 [01:19<00:00,  6.07it/s]
Analysis run on 423 entities from 365 out of 1172 input genes
```

Out[52]: **Table SBY: platelets up, weighted set method, FDR = 0.1.**

	title	pval (uncorrected)	# entities in list	#entities in model	shared entities in gocam	url
0	Collagen biosynthesis and modifying enzymes - Reactome	0.000000	10	12	['PPIB', 'P3H1', 'PLOD3', 'P4HB', 'set:Procollagen C-proteinases', 'set:Prolyl 3-hydroxylases', 'set:COLGALT1,COLGALT2', 'set:4-Hyp collagen propeptides', 'set:Procollagen N-proteinases', 'set:Lysyl hydroxylases']	http://model.geneontology.org/R-HSA-1650814
1	Hedgehog ligand biogenesis - Reactome	0.000005	13	50	['PSMD13', 'PSMA5', 'PSMD11', 'PSMA7', 'PSME2', 'PSMD4', 'PSMB1', 'PSMB7', 'PSMD8', 'PSMB5', 'P4HB', 'PSMB6', 'SYVN1']	http://model.geneontology.org/R-HSA-5358346
2	ER-Phagosome pathway - Reactome	0.000005	13	51	['PSMD13', 'PSMA5', 'PSMD11', 'SEC61B', 'PSMA7', 'PSME2', 'PSMD4', 'PSMB1', 'PSMB7', 'PSMD8', 'PSMB5', 'PSMB6', 'set:SEC61 alpha']	http://model.geneontology.org/R-HSA-1236974
3	Regulation of APC/C activators between G1/S and early anaphase - Reactome	0.000007	13	52	['PSMD13', 'PSMA5', 'PSMD11', 'PSMA7', 'PSME2', 'PSMD4', 'PSMB1', 'PSMB7', 'CDK1', 'PSMD8', 'PSMB5', 'PSMB6', 'set:CDC25']	http://model.geneontology.org/R-HSA-176408
4	Neddylation - Reactome	0.000009	14	62	['UBE2M', 'PSMD13', 'PSMA5', 'PSMD11', 'PSMA7', 'PSME2', 'PSMD4', 'PSMB1', 'CUL9', 'PSMB7', 'PSMD8', 'PSMB5', 'PSMB6', 'set:UCHL3,SENP8']	http://model.geneontology.org/R-HSA-8951664
5	KEAP1-NFE2L2 pathway - Reactome	0.000015	13	55	['PSMD13', 'PSMA5', 'PSMD11', 'PSMA7', 'PSME2', 'PSMD4', 'PSMB1', 'PSMB7', 'CSNK2B', 'PSMD8', 'PSMB5', 'PSMB6', 'set:PRDX1,2,5']	http://model.geneontology.org/R-HSA-9755511
6	The role of GTSE1 in G2/M progression after G2 checkpoint - Reactome	0.000018	12	50	['PSMD13', 'PSMA5', 'PSMD11', 'PSMA7', 'PSME2', 'PSMD4', 'PSMB1', 'CDC25B', 'PSMB7', 'PSMD8', 'PSMB5', 'PSMB6']	http://model.geneontology.org/R-HSA-8852276
7	Conversion from APC/C:Cdc20 to APC/C:Cdh1 in late anaphase - Reactome	0.000018	12	49	['PSMD13', 'PSMA5', 'PSMD11', 'PSMA7', 'PSME2', 'PSMD4', 'PSMB1', 'PSMB7', 'CDK1', 'PSMD8', 'PSMB5', 'PSMB6']	http://model.geneontology.org/R-HSA-176407
8	Degradation of beta-catenin by the destruction complex - Reactome	0.000020	13	54	['PSMD13', 'PSMA5', 'PSMD11', 'PSMA7', 'PSME2', 'PSMD4', 'PSMB1', 'PSMB7', 'PSMD8', 'PSMB5', 'PSMB6', 'set:PP2A-subunit A', 'set:PP2A regulatory subunit B56']	http://model.geneontology.org/R-HSA-195253
9	SCF(Skp2)-mediated degradation of p27/p21 - Reactome	0.000021	12	49	['PSMD13', 'PSMA5', 'PSMD11', 'PSMA7', 'PSME2', 'PSMD4', 'PSMB1', 'PSMB7', 'PSMD8', 'PSMB5', 'PSMB6', 'CKS1B']	http://model.geneontology.org/R-HSA-187577
10	Antigen processing: Ubiquitination & Proteasome degradation - Reactome	0.000022	14	50	['PSMD13', 'PSMA5', 'PSMD11', 'PSMA7', 'PSME2', 'PSMD4', 'PSMB1', 'PSMB7', 'PSMD8', 'PSMB5', 'PSMB6', 'set:E3 ligases in proteasomal degradation', 'set:Activating enzymes E1', 'set:E2 Ubiquitin-conjugating enzyme']	http://model.geneontology.org/R-HSA-983168
11	SCF-beta-TrCP mediated degradation of Emi1 - Reactome	0.000027	12	51	['PSMD13', 'PSMA5', 'PSMD11', 'PSMA7', 'PSME2', 'PSMD4', 'PSMB1', 'PSMB7', 'CDK1', 'PSMD8', 'PSMB5', 'PSMB6']	http://model.geneontology.org/R-HSA-174113
12	Respiratory electron transport - Reactome	0.000031	16	91	['NDUFS8', 'ACADVL', 'COX5B', 'NDUFS5', 'NDUFA8', 'NDUFB7', 'ETFB', 'NDUFB8', 'NDUFV2', 'COX5A', 'COX7C', 'NDUFV1', 'NDUFA11', 'NDUFA13', 'ACADS', 'UQCRC1']	http://model.geneontology.org/R-HSA-611105
13	Antigen Presentation: Folding, assembly and peptide loading of class I MHC - Reactome	0.000037	12	50	['PSMD13', 'PSMA5', 'PSMD11', 'PSMA7', 'PSME2', 'PSMD4', 'PSMB1', 'PSMB7', 'PSMD8', 'PSMB5', 'PSMB6', 'set:ERAP1/2']	http://model.geneontology.org/R-HSA-983170
14	GSK3B and BTRC:CUL1-mediated-degradation of NFE2L2 - Reactome	0.000038	11	44	['PSMD13', 'PSMA5', 'PSMD11', 'PSMA7', 'PSME2', 'PSMD4', 'PSMB1', 'PSMB7', 'PSMD8', 'PSMB5', 'PSMB6']	http://model.geneontology.org/R-HSA-9762114
15	APC/C:Cdc20 mediated degradation of mitotic proteins - Reactome	0.000047	11	45	['PSMD13', 'PSMA5', 'PSMD11', 'PSMA7', 'PSME2', 'PSMD4', 'PSMB1', 'PSMB7', 'PSMD8', 'PSMB5', 'PSMB6']	http://model.geneontology.org/R-HSA-176409
16	Cross-presentation of soluble exogenous antigens (endosomes) - Reactome	0.000047	11	45	['PSMD13', 'PSMA5', 'PSMD11', 'PSMA7', 'PSME2', 'PSMD4', 'PSMB1', 'PSMB7', 'PSMD8', 'PSMB5', 'PSMB6']	http://model.geneontology.org/R-HSA-1236978
17	AUF1 (hnRNP D0) binds and destabilizes mRNA - Reactome	0.000047	11	45	['PSMD13', 'PSMA5', 'PSMD11', 'PSMA7', 'PSME2', 'PSMD4', 'PSMB1', 'PSMB7', 'PSMD8', 'PSMB5', 'PSMB6']	http://model.geneontology.org/R-HSA-450408
18	Regulation of RUNX3 expression and activity - Reactome	0.000055	11	45	['PSMD13', 'PSMA5', 'PSMD11', 'PSMA7', 'PSME2', 'PSMD4', 'PSMB1', 'PSMB7', 'PSMD8', 'PSMB5', 'PSMB6']	http://model.geneontology.org/R-HSA-8941858
19	Regulation of activated PAK-2p34 by proteasome mediated degradation - Reactome	0.000057	11	46	['PSMD13', 'PSMA5', 'PSMD11', 'PSMA7', 'PSME2', 'PSMD4', 'PSMB1', 'PSMB7', 'PSMD8', 'PSMB5', 'PSMB6']	http://model.geneontology.org/R-HSA-211733
20	Ubiquitin-dependent degradation of Cyclin D - Reactome	0.000057	11	46	['PSMD13', 'PSMA5', 'PSMD11', 'PSMA7', 'PSME2', 'PSMD4', 'PSMB1', 'PSMB7', 'PSMD8', 'PSMB5', 'PSMB6']	http://model.geneontology.org/R-HSA-75815
21	Cyclin A:Cdk2-associated events at S phase entry - Reactome	0.000064	12	52	['PSMD13', 'PSMA5', 'PSMD11', 'PSMA7', 'PSME2', 'PSMD4', 'PSMB1', 'PSMB7', 'PSMD8', 'PSMB5', 'PSMB6', 'set:Cdc25 A/B']	http://model.geneontology.org/R-HSA-69656
22	Asymmetric localization of PCP proteins - Reactome	0.000067	11	46	['PSMD13', 'PSMA5', 'PSMD11', 'PSMA7', 'PSME2', 'PSMD4', 'PSMB1', 'PSMB7', 'PSMD8', 'PSMB5', 'PSMB6']	http://model.geneontology.org/R-HSA-4608870
23	Degradation of DVL - Reactome	0.000070	11	47	['PSMD13', 'PSMA5', 'PSMD11', 'PSMA7', 'PSME2', 'PSMD4', 'PSMB1', 'PSMB7', 'PSMD8', 'PSMB5', 'PSMB6']	http://model.geneontology.org/R-HSA-4641258
24	Autodegradation of the E3 ubiquitin ligase COP1 - Reactome	0.000070	11	47	['PSMD13', 'PSMA5', 'PSMD11', 'PSMA7', 'PSME2', 'PSMD4', 'PSMB1', 'PSMB7', 'PSMD8', 'PSMB5', 'PSMB6']	http://model.geneontology.org/R-HSA-349425
25	G2/M Checkpoints - Reactome	0.000070	11	48	['PSMD13', 'PSMA5', 'PSMD11', 'PSMA7', 'PSME2', 'PSMD4', 'PSMB1', 'PSMB7', 'PSMD8', 'PSMB5', 'PSMB6']	http://model.geneontology.org/R-HSA-69481
26	Cyclin E associated events during G1/S transition - Reactome	0.000075	12	51	['PSMD13', 'PSMA5', 'PSMD11', 'PSMA7', 'PSME2', 'PSMD4', 'PSMB1', 'PSMB7', 'PSMD8', 'PSMB5', 'PSMB6', 'set:p-T172-CDK4,p-T177-CDK6:CCND:CDKN1A,CDKN1B,(CDKN1C); p-T172-CDK4,p-T177-CDK6:CCND; (p-T172-CDK4,p-T177-CDK6:CCND:p-Y88-CDKN1B)']	http://model.geneontology.org/R-HSA-69202
27	TNFR2 non-canonical NF-kB pathway - Reactome	0.000094	11	47	['PSMD13', 'PSMA5', 'PSMD11', 'PSMA7', 'PSME2', 'PSMD4', 'PSMB1', 'PSMB7', 'PSMD8', 'PSMB5', 'PSMB6']	http://model.geneontology.org/R-HSA-5668541
28	Degradation of AXIN - Reactome	0.000098	11	48	['PSMD13', 'PSMA5', 'PSMD11', 'PSMA7', 'PSME2', 'PSMD4', 'PSMB1', 'PSMB7', 'PSMD8', 'PSMB5', 'PSMB6']	http://model.geneontology.org/R-HSA-4641257
29	Ubiquitin Mediated Degradation of Phosphorylated Cdc25A - Reactome	0.000102	11	49	['PSMD13', 'PSMA5', 'PSMD11', 'PSMA7', 'PSME2', 'PSMD4', 'PSMB1', 'PSMB7', 'PSMD8', 'PSMB5', 'PSMB6']	http://model.geneontology.org/R-HSA-69601
30	RUNX1 regulates transcription of genes involved in differentiation of HSCs - Reactome	0.000102	11	49	['PSMD13', 'PSMA5', 'PSMD11', 'PSMA7', 'PSME2', 'PSMD4', 'PSMB1', 'PSMB7', 'PSMD8', 'PSMB5', 'PSMB6']	http://model.geneontology.org/R-HSA-8939236
31	Oxygen-dependent proline hydroxylation of Hypoxia-inducible Factor Alpha - Reactome	0.000112	11	48	['PSMD13', 'PSMA5', 'PSMD11', 'PSMA7', 'PSME2', 'PSMD4', 'PSMB1', 'PSMB7', 'PSMD8', 'PSMB5', 'PSMB6']	http://model.geneontology.org/R-HSA-1234176
32	Hedgehog 'on' state - Reactome	0.000117	11	49	['PSMD13', 'PSMA5', 'PSMD11', 'PSMA7', 'PSME2', 'PSMD4', 'PSMB1', 'PSMB7', 'PSMD8', 'PSMB5', 'PSMB6']	http://model.geneontology.org/R-HSA-5632684
33	Hedgehog 'off' state - Reactome	0.000119	11	46	['PSMD13', 'PSMA5', 'PSMD11', 'PSMA7', 'PSME2', 'PSMD4', 'PSMB1', 'PSMB7', 'PSMD8', 'PSMB5', 'PSMB6']	http://model.geneontology.org/R-HSA-5610787
34	FBXL7 down-regulates AURKA during mitotic entry and in early mitosis - Reactome	0.000122	11	50	['PSMD13', 'PSMA5', 'PSMD11', 'PSMA7', 'PSME2', 'PSMD4', 'PSMB1', 'PSMB7', 'PSMD8', 'PSMB5', 'PSMB6']	http://model.geneontology.org/R-HSA-8854050
35	Synthesis of PE - Reactome	0.000124	8	15	['PTDSS2', 'PCYT2', 'PISD', 'PHOSPHO1', 'set:CHK/ETNK', 'set:LPIN', 'set:AGPAT', 'set:PNPLA2/3']	http://model.geneontology.org/R-HSA-1483213
36	Negative regulation of NOTCH4 signaling - Reactome	0.000134	11	49	['PSMD13', 'PSMA5', 'PSMD11', 'PSMA7', 'PSME2', 'PSMD4', 'PSMB1', 'PSMB7', 'PSMD8', 'PSMB5', 'PSMB6']	http://model.geneontology.org/R-HSA-9604323
37	Dectin-1 mediated noncanonical NF-kB signaling - Reactome	0.000140	11	50	['PSMD13', 'PSMA5', 'PSMD11', 'PSMA7', 'PSME2', 'PSMD4', 'PSMB1', 'PSMB7', 'PSMD8', 'PSMB5', 'PSMB6']	http://model.geneontology.org/R-HSA-5607761
38	NIK-->noncanonical NF-kB signaling - Reactome	0.000140	11	50	['PSMD13', 'PSMA5', 'PSMD11', 'PSMA7', 'PSME2', 'PSMD4', 'PSMB1', 'PSMB7', 'PSMD8', 'PSMB5', 'PSMB6']	http://model.geneontology.org/R-HSA-5676590
39	Regulation of expression of SLITs and ROBOs - Reactome	0.000145	11	51	['PSMD13', 'PSMA5', 'PSMD11', 'PSMA7', 'PSME2', 'PSMD4', 'PSMB1', 'PSMB7', 'PSMD8', 'PSMB5', 'PSMB6']	http://model.geneontology.org/R-HSA-9010553
40	Orc1 removal from chromatin - Reactome	0.000166	11	51	['PSMD13', 'PSMA5', 'PSMD11', 'PSMA7', 'PSME2', 'PSMD4', 'PSMB1', 'PSMB7', 'PSMD8', 'PSMB5', 'PSMB6']	http://model.geneontology.org/R-HSA-68949
41	Regulation of PTEN stability and activity - Reactome	0.000172	12	56	['PSMD13', 'PSMA5', 'PSMD11', 'PSMA7', 'PSME2', 'PSMD4', 'PSMB1', 'PSMB7', 'CSNK2B', 'PSMD8', 'PSMB5', 'PSMB6']	http://model.geneontology.org/R-HSA-8948751
42	The proton buffering model - Reactome	0.000183	13	70	['NDUFS8', 'COX5B', 'NDUFS5', 'NDUFA8', 'NDUFB7', 'NDUFB8', 'NDUFV2', 'COX5A', 'COX7C', 'NDUFV1', 'NDUFA11', 'NDUFA13', 'UQCRC1']	http://model.geneontology.org/R-HSA-167827
43	The fatty acid cycling model - Reactome	0.000183	13	70	['NDUFS8', 'COX5B', 'NDUFS5', 'NDUFA8', 'NDUFB7', 'NDUFB8', 'NDUFV2', 'COX5A', 'COX7C', 'NDUFV1', 'NDUFA11', 'NDUFA13', 'UQCRC1']	http://model.geneontology.org/R-HSA-167826
44	Degradation of GLI1 by the proteasome - Reactome	0.000185	11	51	['PSMD13', 'PSMA5', 'PSMD11', 'PSMA7', 'PSME2', 'PSMD4', 'PSMB1', 'PSMB7', 'PSMD8', 'PSMB5', 'PSMB6']	http://model.geneontology.org/R-HSA-5610780
45	Degradation of GLI2 by the proteasome - Reactome	0.000218	11	52	['PSMD13', 'PSMA5', 'PSMD11', 'PSMA7', 'PSME2', 'PSMD4', 'PSMB1', 'PSMB7', 'PSMD8', 'PSMB5', 'PSMB6']	http://model.geneontology.org/R-HSA-5610783
46	GLI3 is processed to GLI3R by the proteasome - Reactome	0.000218	11	52	['PSMD13', 'PSMA5', 'PSMD11', 'PSMA7', 'PSME2', 'PSMD4', 'PSMB1', 'PSMB7', 'PSMD8', 'PSMB5', 'PSMB6']	http://model.geneontology.org/R-HSA-5610785
47	Regulation of RUNX2 expression and activity - Reactome	0.000239	11	54	['PSMD13', 'PSMA5', 'PSMD11', 'PSMA7', 'PSME2', 'PSMD4', 'PSMB1', 'PSMB7', 'PSMD8', 'PSMB5', 'PSMB6']	http://model.geneontology.org/R-HSA-8939902
48	Regulation of RAS by GAPs - Reactome	0.000264	11	48	['PSMD13', 'PSMA5', 'PSMD11', 'PSMA7', 'PSME2', 'PSMD4', 'PSMB1', 'PSMB7', 'PSMD8', 'PSMB5', 'PSMB6']	http://model.geneontology.org/R-HSA-5658442
49	FCERI mediated NF-kB activation - Reactome	0.000307	11	54	['PSMD13', 'PSMA5', 'PSMD11', 'PSMA7', 'PSME2', 'PSMD4', 'PSMB1', 'PSMB7', 'PSMD8', 'PSMB5', 'PSMB6']	http://model.geneontology.org/R-HSA-2871837
50	CLEC7A (Dectin-1) signaling - Reactome	0.000317	11	55	['PSMD13', 'PSMA5', 'PSMD11', 'PSMA7', 'PSME2', 'PSMD4', 'PSMB1', 'PSMB7', 'PSMD8', 'PSMB5', 'PSMB6']	http://model.geneontology.org/R-HSA-5607764
51	Formation of ATP by chemiosmotic coupling - Reactome	0.000358	14	88	['ATP5F1D', 'NDUFS8', 'COX5B', 'NDUFS5', 'NDUFA8', 'NDUFB7', 'NDUFB8', 'NDUFV2', 'COX5A', 'COX7C', 'NDUFV1', 'NDUFA11', 'NDUFA13', 'UQCRC1']	http://model.geneontology.org/R-HSA-163210
52	Cyclin A/B1/B2 associated events during G2/M transition - Reactome	0.000379	7	17	['LCMT1', 'CDC25B', 'CDK1', 'PPP2R2A', 'set:PP2A-subunit A', 'set:CDC25', 'set:CRS kinase']	http://model.geneontology.org/R-HSA-69273
53	Activation of NF-kappaB in B cells - Reactome	0.000383	11	54	['PSMD13', 'PSMA5', 'PSMD11', 'PSMA7', 'PSME2', 'PSMD4', 'PSMB1', 'PSMB7', 'PSMD8', 'PSMB5', 'PSMB6']	http://model.geneontology.org/R-HSA-1169091
54	Heme biosynthesis - Reactome	0.000479	6	14	['UROS', 'COX10', 'HMBS', 'CPOX', 'PPOX', 'set:ALAS1,2']	http://model.geneontology.org/R-HSA-189451
55	Autodegradation of Cdh1 by Cdh1:APC/C - Reactome	0.000507	11	59	['PSMD13', 'PSMA5', 'PSMD11', 'PSMA7', 'PSME2', 'PSMD4', 'PSMB1', 'PSMB7', 'PSMD8', 'PSMB5', 'PSMB6']	http://model.geneontology.org/R-HSA-174084
56	MAPK6/MAPK4 signaling - Reactome	0.000570	12	62	['PSMD13', 'PSMA5', 'PSMD11', 'PSMA7', 'PSME2', 'PSMD4', 'PSMB1', 'PSMB7', 'CDK1', 'PSMD8', 'PSMB5', 'PSMB6']	http://model.geneontology.org/R-HSA-5687128
57	Assembly of the pre-replicative complex - Reactome	0.000583	11	60	['PSMD13', 'PSMA5', 'PSMD11', 'PSMA7', 'PSME2', 'PSMD4', 'PSMB1', 'PSMB7', 'PSMD8', 'PSMB5', 'PSMB6']	http://model.geneontology.org/R-HSA-68867
58	ABC-family proteins mediated transport - Reactome	0.000624	11	54	['PSMD13', 'PSMA5', 'PSMD11', 'PSMA7', 'PSME2', 'PSMD4', 'PSMB1', 'PSMB7', 'PSMD8', 'PSMB5', 'PSMB6']	http://model.geneontology.org/R-HSA-382556
59	APC/C:Cdc20 mediated degradation of Securin - Reactome	0.000764	11	62	['PSMD13', 'PSMA5', 'PSMD11', 'PSMA7', 'PSME2', 'PSMD4', 'PSMB1', 'PSMB7', 'PSMD8', 'PSMB5', 'PSMB6']	http://model.geneontology.org/R-HSA-174154
60	Cdc20:Phospho-APC/C mediated degradation of Cyclin A - Reactome	0.000764	11	62	['PSMD13', 'PSMA5', 'PSMD11', 'PSMA7', 'PSME2', 'PSMD4', 'PSMB1', 'PSMB7', 'PSMD8', 'PSMB5', 'PSMB6']	http://model.geneontology.org/R-HSA-174184
61	APC/C:Cdh1 mediated degradation of Cdc20 and other APC/C:Cdh1 targeted proteins in late mitosis/early G1 - Reactome	0.000764	11	62	['PSMD13', 'PSMA5', 'PSMD11', 'PSMA7', 'PSME2', 'PSMD4', 'PSMB1', 'PSMB7', 'PSMD8', 'PSMB5', 'PSMB6']	http://model.geneontology.org/R-HSA-174178
62	Separation of Sister Chromatids - Reactome	0.000870	11	63	['PSMD13', 'PSMA5', 'PSMD11', 'PSMA7', 'PSME2', 'PSMD4', 'PSMB1', 'PSMB7', 'PSMD8', 'PSMB5', 'PSMB6']	http://model.geneontology.org/R-HSA-2467813
63	CDK-mediated phosphorylation and removal of Cdc6 - Reactome	0.001441	11	65	['PSMD13', 'PSMA5', 'PSMD11', 'PSMA7', 'PSME2', 'PSMD4', 'PSMB1', 'PSMB7', 'PSMD8', 'PSMB5', 'PSMB6']	http://model.geneontology.org/R-HSA-69017
64	Interleukin-1 signaling - Reactome	0.001727	11	64	['PSMD13', 'PSMA5', 'PSMD11', 'PSMA7', 'PSME2', 'PSMD4', 'PSMB1', 'PSMB7', 'PSMD8', 'PSMB5', 'PSMB6']	http://model.geneontology.org/R-HSA-9020702
65	Downstream TCR signaling - Reactome	0.001814	14	74	['PSMD13', 'PSMA5', 'PSMD11', 'PSMA7', 'PSME2', 'PSMD4', 'PSMB1', 'PSMB7', 'PSMD8', 'PSMB5', 'PSMB6', 'CD3D', 'set:PIK3C(1)', 'set:Activator:PI3K']	http://model.geneontology.org/R-HSA-202424
66	A tetrasaccharide linker sequence is required for GAG synthesis - Reactome	0.005201	4	6	['B3GAT3', 'B4GALT7', 'set:XYLT1, XYLT2', 'set:B3GAT dimers']	http://model.geneontology.org/R-HSA-1971475

In [53]:
```
platelets_down_set = enrich.enrich_wrapper('platelets_down.csv','Gene Symbol',method='set',FDR = 0.1,fpath = '../test_data/processed/',table_number = 52)
platelets_down_set
```

```
100%|████████████████████████████████████████| 436/436 [00:00<00:00, 18568.10it/s]
Analysis run on 309 entities from 267 out of 1088 input genes
```

Out[53]: **Table SBZ: platelets down, unweighted set method, FDR = 0.1.**

	title	pval (uncorrected)	# entities in list	#entities in model	shared entities in gocam	url
0	Role of phospholipids in phagocytosis - Reactome	0.000000	11	22	['set:PI3K-regulatory subunit', 'set:PLC beta1,2,3', 'set:PLCbz', 'set:PLC-beta', 'set:G(q) alpha 11,14,15,Q', 'set:PLCdegh', 'set:PL(C)D4:3xCa2+', 'set:PLD', 'set:G-protein gamma subunit', 'set:G-protein alpha (q/11/14/15)', 'set:PLC-beta 1/2/3']	http://model.geneontology.org/R-HSA-2029485
1	DAG and IP3 signaling - Reactome	0.000000	10	18	['set:PI3K-regulatory subunit', 'set:PLC beta1,2,3', 'set:PLCbz', 'set:PLC-beta', 'set:G(q) alpha 11,14,15,Q', 'set:PLCdegh', 'set:PL(C)D4:3xCa2+', 'set:G-protein gamma subunit', 'set:G-protein alpha (q/11/14/15)', 'set:PLC-beta 1/2/3']	http://model.geneontology.org/R-HSA-1489509
2	PLC beta mediated events - Reactome	0.000000	9	15	['set:PLC beta1,2,3', 'set:PLCbz', 'set:PLC-beta', 'set:G(q) alpha 11,14,15,Q', 'set:PLCdegh', 'set:PL(C)D4:3xCa2+', 'set:G-protein gamma subunit', 'set:G-protein alpha (q/11/14/15)', 'set:PLC-beta 1/2/3']	http://model.geneontology.org/R-HSA-112043
3	Antigen activates B Cell Receptor (BCR) leading to generation of second messengers - Reactome	0.000001	11	25	['PIK3R1', 'set:LYN, p-SYK', 'set:PLC beta1,2,3', 'set:PLCbz', 'set:PLC-beta', 'set:G(q) alpha 11,14,15,Q', 'set:PLCdegh', 'set:PL(C)D4:3xCa2+', 'set:G-protein gamma subunit', 'set:G-protein alpha (q/11/14/15)', 'set:PLC-beta 1/2/3']	http://model.geneontology.org/R-HSA-983695
4	G alpha (12/13) signalling events - Reactome	0.000001	8	13	['GNA13', 'set:G-protein alpha (12/13):GTP', 'set:Ligand:GPCR complexes that activate Gi', 'set:ARHGEF11,ARHGEF12', 'set:G-protein G12/G13 (inactive)', 'set:G alpha (i)', 'set:GEFs', 'set:G-protein gamma subunit']	http://model.geneontology.org/R-HSA-416482
5	VEGFR2 mediated cell proliferation - Reactome	0.000002	11	27	['KDR', 'set:PI3K-regulatory subunit', 'set:PLC beta1,2,3', 'set:PLCbz', 'set:PLC-beta', 'set:G(q) alpha 11,14,15,Q', 'set:PLCdegh', 'set:PL(C)D4:3xCa2+', 'set:G-protein gamma subunit', 'set:G-protein alpha (q/11/14/15)', 'set:PLC-beta 1/2/3']	http://model.geneontology.org/R-HSA-5218921
6	Regulation of insulin secretion - Reactome	0.000018	9	22	['set:PLC beta1,2,3', 'set:PLCbz', 'set:PLC-beta', 'set:G(q) alpha 11,14,15,Q', 'set:PLCdegh', 'set:PL(C)D4:3xCa2+', 'set:G-protein gamma subunit', 'set:G-protein alpha (q/11/14/15)', 'set:PLC-beta 1/2/3']	http://model.geneontology.org/R-HSA-422356
7	CLEC7A (Dectin-1) induces NFAT activation - Reactome	0.000018	9	22	['set:PLC beta1,2,3', 'set:PLCbz', 'set:PLC-beta', 'set:G(q) alpha 11,14,15,Q', 'set:PLCdegh', 'set:PL(C)D4:3xCa2+', 'set:G-protein gamma subunit', 'set:G-protein alpha (q/11/14/15)', 'set:PLC-beta 1/2/3']	http://model.geneontology.org/R-HSA-5607763
8	VEGFR2 mediated vascular permeability - Reactome	0.000066	8	20	['PIK3R1', 'PIK3CG', 'set:PI3K-regulatory subunit', 'set:p-S,T-PAK1,2,3', 'set:PAK1,2,3 dimer', 'set:Activator:PI3K', 'set:G-protein gamma subunit', 'set:PI3K alpha, beta, gamma']	http://model.geneontology.org/R-HSA-5218920
9	FCERI mediated Ca+2 mobilization - Reactome	0.000087	9	26	['set:PLC beta1,2,3', 'set:PLCbz', 'set:PLC-beta', 'set:G(q) alpha 11,14,15,Q', 'set:PLCdegh', 'set:PL(C)D4:3xCa2+', 'set:G-protein gamma subunit', 'set:G-protein alpha (q/11/14/15)', 'set:PLC-beta 1/2/3']	http://model.geneontology.org/R-HSA-2871809
10	Ca2+ pathway - Reactome	0.000121	9	27	['set:PLC beta1,2,3', 'set:PLCbz', 'set:PLC-beta', 'set:G(q) alpha 11,14,15,Q', 'set:PLCdegh', 'set:PL(C)D4:3xCa2+', 'set:G-protein gamma subunit', 'set:G-protein alpha (q/11/14/15)', 'set:PLC-beta 1/2/3']	http://model.geneontology.org/R-HSA-4086398
11	Synthesis of very long-chain fatty acyl-CoAs - Reactome	0.000129	6	12	['ELOVL7', 'set:ACSL3,4', 'set:ELOVL3,6,7', 'set:PTPLs', 'set:HSD17B3,12', 'set:ACSBG1,2']	http://model.geneontology.org/R-HSA-75876
12	Synthesis of IP2, IP, and Ins in the cytosol - Reactome	0.000156	7	17	['set:IMPA1/2', 'set:INPP5(3)/ITPK1', 'set:PLCbz', 'set:PLCdegh', 'set:INPP5(4)', 'set:PL(C)D4:3xCa2+', 'set:Inositol polyphosphate 5-phosphatase']	http://model.geneontology.org/R-HSA-1855183
13	G beta:gamma signalling through PI3Kgamma - Reactome	0.000495	5	10	['PIK3R1', 'PIK3CG', 'set:PI3K-regulatory subunit', 'set:G-protein gamma subunit', 'set:PI3K alpha, beta, gamma']	http://model.geneontology.org/R-HSA-392451
14	PI3K/AKT Signaling - Reactome	0.000570	6	15	['PIK3R1', 'PIK3CG', 'set:PI3K-regulatory subunit', 'set:Activator:PI3K', 'set:G-protein gamma subunit', 'set:PI3K alpha, beta, gamma']	http://model.geneontology.org/R-HSA-1257604
15	CD28 dependent PI3K/Akt signaling - Reactome	0.000853	6	16	['PIK3R1', 'PIK3CG', 'set:PI3K-regulatory subunit', 'set:Activator:PI3K', 'set:G-protein gamma subunit', 'set:PI3K alpha, beta, gamma']	http://model.geneontology.org/R-HSA-389357
16	Synthesis of IP3 and IP4 in the cytosol - Reactome	0.001366	5	12	['set:INPP5(3)/ITPK1', 'set:PLCbz', 'set:PLCdegh', 'set:PL(C)D4:3xCa2+', 'set:Inositol polyphosphate 5-phosphatase']	http://model.geneontology.org/R-HSA-1855204

In [54]:
```
platelets_down_standard = enrich.enrich_wrapper('platelets_down.csv','Gene Symbol',method='standard',FDR = 0.1,fpath = '../test_data/processed/',table_number = 53)
platelets_down_standard
```

```
100%|████████████████████████████████████████| 436/436 [00:00<00:00, 18187.14it/s]
```

Out[54]: **Table SCA: platelets down, standard method, FDR = 0.1.**

	title	pval (uncorrected)	# entities in list	#entities in model	shared entities in gocam	url
0	RHOA GTPase cycle - Reactome	0.000007	16	87	['PIK3R1', 'ARHGAP5', 'ARHGAP21', 'OPHN1', 'ARHGEF12', 'ARHGEF3', 'ARHGAP29', 'ARHGAP31', 'STARD8', 'ABR', 'TIAM1', 'PREX2', 'FAM13A', 'ARHGEF15', 'ARHGEF10L', 'ARHGAP24']	http://model.geneontology.org/R-HSA-8980692
1	CDC42 GTPase cycle - Reactome	0.000011	16	90	['PIK3R1', 'ARHGAP5', 'ARHGAP21', 'GNA13', 'OPHN1', 'ARHGEF12', 'ARHGAP29', 'DOCK9', 'ARHGAP31', 'STARD8', 'ABR', 'TIAM1', 'PREX2', 'ARHGEF15', 'PLEKHG1', 'ARHGAP24']	http://model.geneontology.org/R-HSA-9013148
2	RAC1 GTPase cycle - Reactome	0.000044	16	100	['PIK3R1', 'ARHGAP5', 'ARHGAP21', 'GNA13', 'OPHN1', 'ARHGAP29', 'FGD5', 'DOCK9', 'ARHGAP31', 'ABR', 'TIAM1', 'PREX2', 'FAM13A', 'ARHGEF15', 'PLEKHG1', 'ARHGAP24']	http://model.geneontology.org/R-HSA-9013149
3	RHOB GTPase cycle - Reactome	0.000079	9	37	['PIK3R1', 'ARHGAP5', 'ARHGAP21', 'OPHN1', 'ARHGEF12', 'ARHGEF3', 'STARD8', 'ABR', 'ARHGEF10L']	http://model.geneontology.org/R-HSA-9013026

In [55]:
```
platelets_down_weighted = enrich.enrich_wrapper('platelets_down.csv','Gene Symbol',method='ncHGT',FDR = 0.1,fpath = '../test_data/processed/',table_number = 54)
platelets_down_weighted
```

```
100%|████████████████████████████████████████| 436/436 [02:14<00:00,  3.25it/s]
Analysis run on 309 entities from 267 out of 1088 input genes
```

Out[55]: **Table SCB: platelets down, weighted set method, FDR = 0.1.**

	title	pval (uncorrected)	# entities in list	#entities in model	shared entities in gocam	url
0	Role of phospholipids in phagocytosis - Reactome	0.000010	11	22	['set:PI3K-regulatory subunit', 'set:PLC beta1,2,3', 'set:PLCbz', 'set:PLC-beta', 'set:G(q) alpha 11,14,15,Q', 'set:PLCdegh', 'set:PL(C)D4:3xCa2+', 'set:PLD', 'set:G-protein gamma subunit', 'set:G-protein alpha (q/11/14/15)', 'set:PLC-beta 1/2/3']	http://model.geneontology.org/R-HSA-2029485
1	DAG and IP3 signaling - Reactome	0.000019	10	18	['set:PI3K-regulatory subunit', 'set:PLC beta1,2,3', 'set:PLCbz', 'set:PLC-beta', 'set:G(q) alpha 11,14,15,Q', 'set:PLCdegh', 'set:PL(C)D4:3xCa2+', 'set:G-protein gamma subunit', 'set:G-protein alpha (q/11/14/15)', 'set:PLC-beta 1/2/3']	http://model.geneontology.org/R-HSA-1489509
2	VEGFR2 mediated cell proliferation - Reactome	0.000046	11	27	['KDR', 'set:PI3K-regulatory subunit', 'set:PLC beta1,2,3', 'set:PLCbz', 'set:PLC-beta', 'set:G(q) alpha 11,14,15,Q', 'set:PLCdegh', 'set:PL(C)D4:3xCa2+', 'set:G-protein gamma subunit', 'set:G-protein alpha (q/11/14/15)', 'set:PLC-beta 1/2/3']	http://model.geneontology.org/R-HSA-5218921
3	PLC beta mediated events - Reactome	0.000050	9	15	['set:PLC beta1,2,3', 'set:PLCbz', 'set:PLC-beta', 'set:G(q) alpha 11,14,15,Q', 'set:PLCdegh', 'set:PL(C)D4:3xCa2+', 'set:G-protein gamma subunit', 'set:G-protein alpha (q/11/14/15)', 'set:PLC-beta 1/2/3']	http://model.geneontology.org/R-HSA-112043
4	Antigen activates B Cell Receptor (BCR) leading to generation of second messengers - Reactome	0.000071	11	25	['PIK3R1', 'set:LYN, p-SYK', 'set:PLC beta1,2,3', 'set:PLCbz', 'set:PLC-beta', 'set:G(q) alpha 11,14,15,Q', 'set:PLCdegh', 'set:PL(C)D4:3xCa2+', 'set:G-protein gamma subunit', 'set:G-protein alpha (q/11/14/15)', 'set:PLC-beta 1/2/3']	http://model.geneontology.org/R-HSA-983695
5	CLEC7A (Dectin-1) induces NFAT activation - Reactome	0.000403	9	22	['set:PLC beta1,2,3', 'set:PLCbz', 'set:PLC-beta', 'set:G(q) alpha 11,14,15,Q', 'set:PLCdegh', 'set:PL(C)D4:3xCa2+', 'set:G-protein gamma subunit', 'set:G-protein alpha (q/11/14/15)', 'set:PLC-beta 1/2/3']	http://model.geneontology.org/R-HSA-5607763
6	Regulation of insulin secretion - Reactome	0.000444	9	22	['set:PLC beta1,2,3', 'set:PLCbz', 'set:PLC-beta', 'set:G(q) alpha 11,14,15,Q', 'set:PLCdegh', 'set:PL(C)D4:3xCa2+', 'set:G-protein gamma subunit', 'set:G-protein alpha (q/11/14/15)', 'set:PLC-beta 1/2/3']	http://model.geneontology.org/R-HSA-422356

Non Alcoholic Fatty Liver Disease

Govaere et al., 2023

https://www.nature.com/articles/s42255-023-00775-1

NAFLD. Paired transcriptomics and proteomics in 306 patients to find signatures of active steatohepatitis and severe fibrosis.

In [56]:
```
GS2_step= enrich.enrich_wrapper('Goavere_S2.csv','Gene Symbol',method='set',FDR = 0.1,fpath = '../test_data/processed/', table_number = 55)
GS2_step
```

```
100%|████████████████████████████████████████| 98/98 [00:00<00:00, 17799.41it/s]
Analysis run on 68 entities from 48 out of 118 input genes
```

Out[56]: **Table SCC: NAFLD combined up/downregulated genes, unweighted set method, FDR = 0.1.**

	title	pval (uncorrected)	# entities in list	#entities in model	shared entities in gocam	url
0	Activation of Matrix Metalloproteinases - Reactome	0.000000	7	18	['set:Trypsin, plasmin', 'set:proMMP9 activating proteases', 'set:proMMP8 initial activators', 'set:proMMP3 initial activators', 'set:proMMP1 initial activators', 'set:proMMP10 activators', 'set:MMP7 initial activators']	http://model.geneontology.org/R-HSA-1592389
1	Cytosolic sulfonation of small molecules - Reactome	0.000012	5	19	['SULT2A1', 'set:SULT1E1,2A1', 'set:SULT dimers (T3)', 'set:SULTs active on DHEA', 'set:SULT dimers (T2)']	http://model.geneontology.org/R-HSA-156584

In [57]:
```
GS2_standard = enrich.enrich_wrapper('Goavere_S2.csv','Gene Symbol',method='standard',FDR = 0.1,fpath = '../test_data/processed/', table_number = 56)
GS2_standard
```

```
100%|████████████████████████████████████████| 98/98 [00:00<00:00, 17303.38it/s]
```

Out[57]: **Table SCD: NAFLD combined up/downregulated genes, standard method, FDR = 0.1.**

title	pval (uncorrected)	# entities in list	#entities in model	shared entities in gocam	url

In [58]:
```
GS2_weighted = enrich.enrich_wrapper('Goavere_S2.csv','Gene Symbol',method='ncHGT',FDR = 0.1,fpath = '../test_data/processed/', table_number = 57)
GS2_weighted
```

```
100%|████████████████████████████████████████| 98/98 [00:01<00:00, 84.44it/s]
Analysis run on 68 entities from 48 out of 118 input genes
```

Out[58]: **Table SCE: NAFLD combined up/downregulated genes, weighted set method, FDR = 0.1.**

	title	pval (uncorrected)	# entities in list	#entities in model	shared entities in gocam	url
0	Activation of Matrix Metalloproteinases - Reactome	0.000000	7	18	['set:Trypsin, plasmin', 'set:proMMP9 activating proteases', 'set:proMMP8 initial activators', 'set:proMMP3 initial activators', 'set:proMMP1 initial activators', 'set:proMMP10 activators', 'set:MMP7 initial activators']	http://model.geneontology.org/R-HSA-1592389
1	Cytosolic sulfonation of small molecules - Reactome	0.000130	5	19	['SULT2A1', 'set:SULT1E1,2A1', 'set:SULT dimers (T3)', 'set:SULTs active on DHEA', 'set:SULT dimers (T2)']	http://model.geneontology.org/R-HSA-156584

Astrocytes in Aging Mouse Brains across 4 brain regions

Boisvert et al., 2018

https://www.sciencedirect.com/science/article/pii/S221112471731848X?via%3Dihub#app3

RNA seq on astrocytes isolated from different brain regions in 4 month and 2 year old mice. Most brain regions had ~200 up and ~200 downregulated genes, but most of those were also differentially expressed between brain regions.

In fig 2B, it appears that cerebellum and hypothalamus are the best candidates, as the 3 biological replicates (3 mice) cluster together by region and age. The hypothalamus looks a little cleaner than cerebellum.

The paper says they filtered based on "FPKM > 1, Adj P value < 0.05, and pulldown/input > 0.75"

Hypothalamus

In [59]:
```
astro_HTH_set = enrich.enrich_wrapper('astro_HTH_comb.csv','Gene Symbol',method='set',FDR = 0.1,fpath = '../test_data/processed/',table_number = 58)
astro_HTH_set
```

```
100%|████████████████████████████████████████| 246/246 [00:00<00:00, 18296.58it/s]
Analysis run on 139 entities from 115 out of 416 input genes
```

Out[59]: **Table SCF: astroctyes hypothalamus combined up/downregulated genes, unweighted set method, FDR = 0.1.**

	title	pval (uncorrected)	# entities in list	#entities in model	shared entities in gocam	url
0	Cholesterol biosynthesis - Reactome	0.000000	10	22	['HMGCS1', 'HSD17B7', 'SQLE', 'MSMO1', 'DHCR24', 'MVD', 'ACLY', 'FDFT1', 'LSS', 'CYP51A1']	http://model.geneontology.org/R-HSA-191273
1	Cytosolic sulfonation of small molecules - Reactome	0.000377	5	19	['SULT1A1', 'set:PAPSS1,2', 'set:SULT dimers (T3)', 'set:SULT1A1,A2,C2,4A1', 'set:SULT dimers (T2)']	http://model.geneontology.org/R-HSA-156584
2	Regulation of Complement cascade - Reactome	0.000387	4	11	['C3', 'set:C5 convertases', 'set:CD46, CR1:C4b:C3b complexes', 'set:C3 convertases']	http://model.geneontology.org/R-HSA-977606
3	Bloch pathway - Reactome	0.000395	3	5	['HSD17B7', 'DHCR7', 'DHCR24']	http://model.geneontology.org/R-HSA-6807047
4	Kandutsch-Russell pathway - Reactome	0.000395	3	5	['HSD17B7', 'DHCR7', 'DHCR24']	http://model.geneontology.org/R-HSA-6807062

In [60]:
```
astro_HTH_standard = enrich.enrich_wrapper('astro_HTH_comb.csv','Gene Symbol',method='standard',FDR = 0.1,fpath = '../test_data/processed/',table_number = 59)
astro_HTH_standard
```

```
100%|████████████████████████████████████████| 246/246 [00:00<00:00, 17699.00it/s]
```

Out[60]: **Table SCG: astroctyes hypothalamus combined up/downregulated genes, standard method, FDR = 0.1.**

	title	pval (uncorrected)	# entities in list	#entities in model	shared entities in gocam	url
0	Cholesterol biosynthesis - Reactome	0.000000	10	24	['HMGCS1', 'HSD17B7', 'SQLE', 'MSMO1', 'DHCR24', 'MVD', 'ACLY', 'FDFT1', 'LSS', 'CYP51A1']	http://model.geneontology.org/R-HSA-191273
1	Terminal pathway of complement - Reactome	0.000001	4	5	['C3', 'C4B', 'C4B', 'C2']	http://model.geneontology.org/R-HSA-166665
2	Peptide ligand-binding receptors - Reactome	0.000071	4	11	['C3', 'C4B', 'C4B', 'C2']	http://model.geneontology.org/R-HSA-375276
3	Kandutsch-Russell pathway - Reactome	0.000107	3	5	['HSD17B7', 'DHCR7', 'DHCR24']	http://model.geneontology.org/R-HSA-6807062
4	Bloch pathway - Reactome	0.000107	3	5	['HSD17B7', 'DHCR7', 'DHCR24']	http://model.geneontology.org/R-HSA-6807047

Note on C4B:

C4B is listed twice, but that is due to P0C0L4 and P0C0L5 both being officially listed as uniprot accession IDs for C4B, which affected our results when we used SimpleMine.

P0C0L4 seems to actually correspond to C4A (C4A and C4B are paralogs). However, C4b can be derived from either the C4A gene product or the C4B gene product, and so both genes are listed as members of a set, and both are annotated to the pathway. GO-CAMs use uniprot accession IDs, so P0C0L4 and P0C0L5 are listed in the model for enrichment. When the input list of genes was mapped from gene symbols to uniprot accession IDs, both P0C0L4 and P0C0L5 were hits for the GOCAM.

Why both accession IDs are officially associated with C4B is unclear, since they correspond to C4A and C4B on UniProt's website. We believe the issue is that the genes are cross listed as aliases for one another, which led to both uniprot accession ID's being associated with C4B.

In [61]:
```
astro_HTH_weighted = enrich.enrich_wrapper('astro_HTH_comb.csv','Gene Symbol',method='ncHGT',FDR = 0.1,fpath = '../test_data/processed/',table_number = 60)
astro_HTH_weighted
```

```
100%|████████████████████████████████████████| 246/246 [00:30<00:00,  7.97it/s]
Analysis run on 139 entities from 115 out of 416 input genes
```

Out[61]: **Table SCH: astroctyes hypothalamus combined up/downregulated genes, weighted set method, FDR = 0.1.**

	title	pval (uncorrected)	# entities in list	#entities in model	shared entities in gocam	url
0	Cholesterol biosynthesis - Reactome	0.000000	10	22	['HMGCS1', 'HSD17B7', 'SQLE', 'MSMO1', 'DHCR24', 'MVD', 'ACLY', 'FDFT1', 'LSS', 'CYP51A1']	http://model.geneontology.org/R-HSA-191273

Cerebellum

In [62]:
```
astro_CB_set = enrich.enrich_wrapper('astro_CB_comb.csv','Gene Symbol',method='set',FDR = 0.1,fpath = '../test_data/processed/',table_number = 61)
astro_CB_set
```

```
100%|████████████████████████████████████████| 310/310 [00:00<00:00, 18129.06it/s]
Analysis run on 200 entities from 172 out of 605 input genes
```

Out[62]: **Table SCI: astroctyes cerebellum combined up/downregulated genes, unweighted set method, FDR = 0.1.**

title	pval (uncorrected)	# entities in list	#entities in model	shared entities in gocam	url

In [63]:
```
astro_CB_standard = enrich.enrich_wrapper('astro_CB_comb.csv','Gene Symbol',method='standard',FDR = 0.1,fpath = '../test_data/processed/',table_number = 62)
astro_CB_standard
```

```
100%|████████████████████████████████████████| 310/310 [00:00<00:00, 18286.12it/s]
```

Out[63]: **Table SCJ: astroctyes cerebellum combined up/downregulated genes, standard method, FDR = 0.1.**

title	pval (uncorrected)	# entities in list	#entities in model	shared entities in gocam	url

In [64]:
```
astro_CB_weighted = enrich.enrich_wrapper('astro_CB_comb.csv','Gene Symbol',method='ncHGT',FDR = 0.1,fpath = '../test_data/processed/',table_number = 63)
astro_CB_weighted
```

```
100%|████████████████████████████████████████| 310/310 [00:28<00:00, 10.79it/s]
Analysis run on 200 entities from 172 out of 605 input genes
```

Out[64]: **Table SCK: astroctyes cerebellum combined up/downregulated genes, weighted set method, FDR = 0.1.**

title	pval (uncorrected)	# entities in list	#entities in model	shared entities in gocam	url

Macrophage activation

Orrechioni et al., 2019

https://www.ncbi.nlm.nih.gov/pmc/articles/PMC6543837/pdf/fimmu-10-01084.pdf

In vitro classically activated vs alternatively activated macrophpages

In [65]:
```
mac_set = enrich.enrich_wrapper('mac_comb.csv','Gene Symbol',method='set',FDR = 0.1,fpath = '../test_data/processed/',table_number = 64)
mac_set
```

```
100%|████████████████████████████████████████| 667/667 [00:00<00:00, 18667.85it/s]
Analysis run on 619 entities from 511 out of 1519 input genes
```

Out[65]: **Table SCL: macrophages combined up/downregulated genes, unweighted set method, FDR = 0.1.**

	title	pval (uncorrected)	# entities in list	#entities in model	shared entities in gocam	url
0	Resolution of Sister Chromatid Cohesion - Reactome	0.000057	30	95	['BIRC5', 'CDC20', 'KIF2C', 'CENPF', 'CENPA', 'NUF2', 'ZWILCH', 'AURKB', 'CENPN', 'SPC24', 'KNTC1', 'CENPK', 'PLK1', 'SKA1', 'CDCA5', 'CENPI', 'BUB1', 'BUB1B', 'CENPP', 'CENPH', 'CDCA8', 'CENPT', 'CDK1', 'NDC80', 'ERCC6L', 'SPC25', 'CENPE', 'CENPM', 'MAD2L1', 'set:CCNB1,CCNB2:p-T161-CDK1']	http://model.geneontology.org/R-HSA-2500257
1	Interleukin-35 Signalling - Reactome	0.000057	7	9	['IL6ST', 'JAK2', 'STAT4', 'EBI3', 'STAT1', 'IL12A', 'set:JAK1, JAK2, (TYK2)']	http://model.geneontology.org/R-HSA-8984722
2	Unwinding of DNA - Reactome	0.000084	6	7	['MCM5', 'MCM7', 'MCM2', 'MCM3', 'MCM4', 'MCM6']	http://model.geneontology.org/R-HSA-176974

In [66]:
```
mac_standard = enrich.enrich_wrapper('mac_comb.csv','Gene Symbol',method='standard',FDR = 0.1,fpath = '../test_data/processed/',table_number = 65)
mac_standard
```

```
100%|████████████████████████████████████████| 667/667 [00:00<00:00, 18326.66it/s]
```

Out[66]: **Table SCM: macrophages combined up/downregulated genes, standard method, FDR = 0.1.**

	title	pval (uncorrected)	# entities in list	#entities in model	shared entities in gocam	url
0	Resolution of Sister Chromatid Cohesion - Reactome	0.000000	30	106	['CCNB2', 'BIRC5', 'CDC20', 'KIF2C', 'CENPF', 'CENPA', 'NUF2', 'ZWILCH', 'AURKB', 'CENPN', 'SPC24', 'KNTC1', 'CENPK', 'PLK1', 'SKA1', 'CDCA5', 'CENPI', 'BUB1', 'BUB1B', 'CENPP', 'CENPH', 'CDCA8', 'CENPT', 'CDK1', 'NDC80', 'ERCC6L', 'SPC25', 'CENPE', 'CENPM', 'MAD2L1']	http://model.geneontology.org/R-HSA-2500257
1	Unwinding of DNA - Reactome	0.000005	6	7	['MCM5', 'MCM7', 'MCM2', 'MCM3', 'MCM4', 'MCM6']	http://model.geneontology.org/R-HSA-176974
2	Interleukin-35 Signalling - Reactome	0.000055	6	9	['IL6ST', 'JAK2', 'STAT4', 'EBI3', 'STAT1', 'IL12A']	http://model.geneontology.org/R-HSA-8984722

In [67]:
```
mac_weighted = enrich.enrich_wrapper('mac_comb.csv','Gene Symbol',method='ncHGT',FDR = 0.1,fpath = '../test_data/processed/',table_number = 66)
mac_weighted
```

```
100%|████████████████████████████████████████| 667/667 [03:13<00:00,  3.44it/s]
Analysis run on 619 entities from 511 out of 1519 input genes
```

Out[67]: **Table SCN: macrophages combined up/downregulated genes, weighted set method, FDR = 0.1.**

	title	pval (uncorrected)	# entities in list	#entities in model	shared entities in gocam	url
0	Resolution of Sister Chromatid Cohesion - Reactome	0.000000	30	95	['BIRC5', 'CDC20', 'KIF2C', 'CENPF', 'CENPA', 'NUF2', 'ZWILCH', 'AURKB', 'CENPN', 'SPC24', 'KNTC1', 'CENPK', 'PLK1', 'SKA1', 'CDCA5', 'CENPI', 'BUB1', 'BUB1B', 'CENPP', 'CENPH', 'CDCA8', 'CENPT', 'CDK1', 'NDC80', 'ERCC6L', 'SPC25', 'CENPE', 'CENPM', 'MAD2L1', 'set:CCNB1,CCNB2:p-T161-CDK1']	http://model.geneontology.org/R-HSA-2500257
1	Interleukin-35 Signalling - Reactome	0.000016	7	9	['IL6ST', 'JAK2', 'STAT4', 'EBI3', 'STAT1', 'IL12A', 'set:JAK1, JAK2, (TYK2)']	http://model.geneontology.org/R-HSA-8984722
2	Unwinding of DNA - Reactome	0.000029	6	7	['MCM5', 'MCM7', 'MCM2', 'MCM3', 'MCM4', 'MCM6']	http://model.geneontology.org/R-HSA-176974
3	Cyclin A/B1/B2 associated events during G2/M transition - Reactome	0.000334	8	17	['PLK1', 'WEE1', 'CDK1', 'CHEK1', 'PKMYT1', 'CDC25B', 'set:CDC25', 'set:CRS kinase']	http://model.geneontology.org/R-HSA-69273
4	RHO GTPases activate PAKs - Reactome	0.000373	9	20	['PAK1', 'CIT', 'VAV1', 'PPP1R12B', 'set:ELMO1,ELMO2', 'set:CDC42:GTP,RAC1:GTP:PAK1,PAK2,PAK3', 'set:p-S,T-PAK1,2,3', 'set:DOCK1,TRIO', 'set:p-S,T-PAK1,2']	http://model.geneontology.org/R-HSA-5627123
